# The complete sequence of the silkworm W chromosome uncovers its rapid evolution by large-scale duplications/deletions and translocation of W-linked genes

**DOI:** 10.1093/g3journal/jkag170

**Published:** 2026-06-30

**Authors:** Wanshun Li, Hiroaki Abe, Yue Chen, Jianqiu Liu, Yanjue Wang, Masahiro Ajimura, Shenglong Li, Yingdan Xiao, Huizhen Guo, Jiaqi Wu, Yajing Xu, Xue Yang, XiaoXue Yang, Shaopeng Hao, Qihue He, Tsuguru Fujii, Ken Sahara, Atsuo Yoshido, Yusuke Takehana, Kiyoshi Naruse, Kallare P Arunkumar, Marian R Goldsmith, Guy Smagghe, František Marec, Qingyou Xia, Kazuei Mita

**Affiliations:** Integrative Science Center of Germplasm Creation in Western China (Chongqing) Science City, Biological Science Research Center, Southwest University, Chongqing 400715, China; Department of Biological Production, Faculty of Agriculture, Tokyo University of Agriculture and Technology Fuchu, Tokyo 183-8509, Japan; Integrative Science Center of Germplasm Creation in Western China (Chongqing) Science City, Biological Science Research Center, Southwest University, Chongqing 400715, China; Integrative Science Center of Germplasm Creation in Western China (Chongqing) Science City, Biological Science Research Center, Southwest University, Chongqing 400715, China; Integrative Science Center of Germplasm Creation in Western China (Chongqing) Science City, Biological Science Research Center, Southwest University, Chongqing 400715, China; Integrative Science Center of Germplasm Creation in Western China (Chongqing) Science City, Biological Science Research Center, Southwest University, Chongqing 400715, China; Integrative Science Center of Germplasm Creation in Western China (Chongqing) Science City, Biological Science Research Center, Southwest University, Chongqing 400715, China; Integrative Science Center of Germplasm Creation in Western China (Chongqing) Science City, Biological Science Research Center, Southwest University, Chongqing 400715, China; Integrative Science Center of Germplasm Creation in Western China (Chongqing) Science City, Biological Science Research Center, Southwest University, Chongqing 400715, China; Graduate School of Integrated Sciences for Life, Hiroshima University, Higashi-Hiroshima, Hiroshima 739-8528, Japan; Integrative Science Center of Germplasm Creation in Western China (Chongqing) Science City, Biological Science Research Center, Southwest University, Chongqing 400715, China; Integrative Science Center of Germplasm Creation in Western China (Chongqing) Science City, Biological Science Research Center, Southwest University, Chongqing 400715, China; Integrative Science Center of Germplasm Creation in Western China (Chongqing) Science City, Biological Science Research Center, Southwest University, Chongqing 400715, China; Integrative Science Center of Germplasm Creation in Western China (Chongqing) Science City, Biological Science Research Center, Southwest University, Chongqing 400715, China; Integrative Science Center of Germplasm Creation in Western China (Chongqing) Science City, Biological Science Research Center, Southwest University, Chongqing 400715, China; Laboratory of Silkworm Genetic Resources, Institute of Genetic Resources, Kyushu University Graduate School of Bioresources and Environmental Science, Motooka 744, Fukuoka 819-0395, Japan; Faculty of Agriculture, Iwate University, Morioka, Iwate 020-8550, Japan; Biology Centre CAS, Institute of Entomology, České Budějovice 370 05, Czech Republic; Department of Animal Bio-Science, Faculty of Bio-Science, Nagahama Institute of Bio-Science and Technology, 1266 Tamura, Nagahama, Shiga 526-0829, Japan; Laboratory of Bioresources, National Institute for Basic Biology, Okazaki, Aichi 444-8585, Japan; Institute for Seri-Biotechnological Research, Central Silk Board, Bangalore, Karnataka 560035, India; Woodward Hall, University of Rhode Island, Kingston, RI 02881, United States; Institute of Entomology, Guizhou University, Guiyang, Guizhou 550025, China; Department of Biology, Vrije Universiteit Brussel (VUB), Brussels 1050, Belgium; Biology Centre CAS, Institute of Entomology, České Budějovice 370 05, Czech Republic; Integrative Science Center of Germplasm Creation in Western China (Chongqing) Science City, Biological Science Research Center, Southwest University, Chongqing 400715, China; Integrative Science Center of Germplasm Creation in Western China (Chongqing) Science City, Biological Science Research Center, Southwest University, Chongqing 400715, China

**Keywords:** T2T_W chromosome sequence, W chromosome evolution, silkworm, full LTR and LINE retrotransposons, large-scale amplifications and deletions

## Abstract

The complete sequence of the W chromosome, which carries feminization activity in the silkworm, is crucial for understanding the sex-determination system in Lepidoptera. However, extensive accumulation of transposons due to lack of recombination, the very rare protein-coding genes and almost no information about molecular markers has hindered full W sequencing. We report the first complete silkworm W sequence (T2T_W, 11683305 bp) obtained by combining sequencing-assembly technologies and newly developed error detection methods, evaluated with genetically mapped W-RAPD markers, W-mutants, and W-derived BAC clones. The T2T_W sequence showed that the W is composed of a massive 92% accumulation of transposons and repeat sequences, among which the main constituents are intact LTR/LINE retrotransposons indicating recent expansions. In addition to *Fem* clusters producing *Fem* piRNA (*Feminizer*-derived PIWI-interacting RNA), we found 26 protein-coding genes in the W sequence. These include four gene pairs encoding zinc-finger motifs designated *z1:z20* and a gene encoding serine/arginine repetitive matrix protein 1-like (SRRM1-like). To identify candidate genes for female sex-determination and differentiation we also sequenced the shortest W (3.8 Mb) from a translocation mutant with feminizing activity, which harbored four conventional genes: a *Fem* cluster, a pair of *z1:z20* isoforms, *z20-S*, and a *SRRM1-like* gene. Phylogenetic analysis revealed that *z1:z20* originated from a copy of an autosomal zinc-finger gene pair, *z2:z21*, translocated onto the W around 2.43 Mya and subsequently amplified to yield 4 W-linked zinc-finger gene pairs. The complete W sequence revealed that large-scale deletions and amplifications played a significant role in W chromosome evolution.

## Introduction

*Bombyx mori* is an organism with female heterogamety (Tanaka 1916), wherein female sex is determined by the presence of a single W chromosome irrespective of the number of autosomes or its paired Z chromosome (Hashimoto 1933). Silkworm geneticists used W chromosome-deletion mutants produced by X-ray irradiation (Tazima et al. 1951) to develop a genetic map for this chromosome containing 11 W-linked RAPD markers (Abe et al. 2005) and found a primary feminizing factor in a 10% portion of the W (Abe et al. 2008). Complete sequencing of the W has been hindered by extensive accumulation of transposons and repetitive motifs, the absence of conventional genes, and almost no information about its molecular markers. Although three recent papers reported complete sequencing of the silkworm W chromosome (Han et al. 2024; Lee et al. 2024; Wan et al. 2025), the W sequence from Lee et al. (2024) was found to include a *Bombyx mandarina*-specific W-RAPD marker (Yamato) (Abe et al. 2002) and lacked most of the previously mapped *B. mori* W-linked RAPD markers (Abe et al. 2005), indicating it could not be a complete *B. mori* W sequence. The W sequence from Han et al. (2024) was also incomplete, reporting a highly fragmented structure containing 151 gaps and no read coverage data, which should be reported for a high-quality sequence. The third W sequence is more complete than the other two, but the assembly is characterized by a fragmented and disorganized structure (see Results). Thus, to date no complete and accurate W chromosome sequence is available for the silkworm or any lepidopteran species.

Identification of the primary determiner of sex in the silkworm has long been a focus of study. This would pave the way not only for understanding the mechanism of female sex determination in silkworm and other lepidopterans, but also for devising new genetically based control measures for lepidopteran pests. *Fem* piRNA (*Feminizer*-derived PIWI-interacting RNA) is well established as an early acting component of the silkworm sex-determination cascade derived from the W chromosome (Kiuchi et al. 2014); further, its role in silkworm development and sexual differentiation was defined using CRISPR/Cas9 knockout mutations (Li et al. 2018b; Kiuchi et al. 2023). Several additional protein-coding genes are known to be involved in male-specific splicing of the *Bmdsx* gene, which encodes a transcription factor acting as a well-conserved molecular switch at the bottom of the sex-determination cascade. These include the Z chromosome-linked CCCH-type zinc-finger protein *BmMasc* (Kiuchi et al. 2014; Katsuma et al. 2018; Zhao et al. 2019), the P-element somatic inhibitor (*BmPSI*) (Suzuki et al. 2008; Xu et al. 2017; Kaneda et al. 2025), the IGF-II mRNA binding protein *BmImp* (Suzuki et al. 2010), and *znf-2*, a CCCH-type zinc-finger protein gene located on chromosome 25 (Gopinath et al. 2016; Yang et al. 2021). Recently, it has been reported that znf-2 interacts with BmMasc and enhances BmMasc-dependent masculinization (Fukui et al. 2026). However, too little is known about W-linked genes to describe the complete female sex-determination pathway. This necessitates obtaining a complete and accurate sequence of the silkworm W chromosome.

Here, we report a more complete silkworm W sequence (T2T_W, 11683305 bp) than has been published. To overcome the difficulties in identifying and sequencing its highly repetitious W DNA, we used current common methods for genome sequencing and contig construction to which we developed and added new methods to overcome technical difficulties inherent in working with such a complex system. We also took advantage of certain traditional silkworm-specific genetic resources, namely, strains carrying irradiation-induced mutations not investigated in the previous studies. New technical approaches applied here include development of a simple “*k*-mer Quotient” (KQ) method to obtain W-linked reads (Li et al. 2018a), and two methods for detecting and correcting errors in assembly, a newly named “Perfect-mapped reads Based assembly error Detection” (PBD) method and a “Local assembly Based error Correction” (LABC) method (Li et al. 2024). We followed up de novo sequencing and assembly of the W chromosome by long-range structural evaluation using genetically mapped silkworm W-RAPD markers and W-chromosome deletion mutants, and short-range evaluation using full-length read coverage and mapping of W-derived BAC clones (W-BACs). As noted, in parallel we sequenced the shortest available W-translocation mutant with feminization activity, “sex-limited yellow cocoon” (yCs) (Abe et al. 2008). Our complete T2T_W sequence, which is now more complete and accurate than the 3 previously published W sequences, provides new information on W-linked genes and reveals that the W is evolving rapidly at the molecular level.

## Materials and methods

### Silkworm strains and W-deleted mutants used in this study

Inbred strains Dazao and p50T were used for preparation of genomic DNA for W chromosome sequencing. The two strains are historically related and essentially the same. Originally the Dazao strain was imported from China to Japan, renamed p50T, and reared continuously at the University of Tokyo since then. Larvae were reared on fresh mulberry leaves at 25 °C (day/night, 12/12 h). A nondiapausing strain, D9L, which was derived from the Dazao strain, was used for experiments of transcription in early embryogenesis.

Hashimoto (1948) was able to fuse the W chromosome with a fragment of chromosome 3 bearing the *Ze* (zebra) marker using X-ray irradiation. In the resulting strain, sex-limited zebra (Zebra-W chromosome) female larvae have striped “zebra” markings, whereas male larvae have normal markings (whitish skin).

The sex-limited yellow cocoon W (yCsW) strain was generated by translocating an irradiation-induced fragment of the W to chromosome 2 (T(W;2)Y-Chu), which carries a yellow cocoon marker (*Y*), which allows tracking it in the common white skinned wild type genetic background. Further deletion in the W region of this strain created the mutant (T(W;2)Y-Abe), which has only 10% of the original W but still gives rise to fully fertile females (Fig. 2) (Abe et al. 2008).

The DfZ-DfW chromosome has been maintained as a female-killing chromosome in the Z101 strain (Tazima 1952). The DfZ-DfW chromosome is composed of the *Fem* locus-defective Df(+*^od^ p^Sa^* +*^p^* W) chromosome and a Z chromosome fragment. See Fujii et al. (2006) for construction of this chromosome which contains RAPD markers W-Bonsai, W-Samurai, and W-Mikan (Fig. 2a).

### Sequencing and assembly of the W chromosome

#### Sample preparation and genome sequencing

*B*. *mori* strains Dazao and p50T were reared and maintained at SWU. For each strain, single female–male pairs were mated followed by separately rearing the larvae from eggs laid by each individual female. The pupae were sampled for DNA.

Library construction and DNA sequencing of the samples were performed by the Beijing Genomics Institute (BGI)-Wuhan and Nextomics Biosciences (Wuhan, China) (collectively BGI-tech). A single female pupa was used to extract genomic DNA named “femaleHIFI” and “femaleNGS,” which was sequenced by HIFI (Sequel II) and NGS (MGISEQ-2000) at BGI-tech. HIFI data were produced by SMRT Link (PacBio, Pacific Biosciences, California) using a CCS model with the parameter “–minPasses 3 –min-rq 0.99.” Mixed male pupal DNA termed “maleNGS” was also used to produce NGS data by BGI-tech. DNA was extracted from a second single female pupa for Ultra-long Oxford Nanopore Technologies (ONT) using the Nanopore PromethION system (Nextomics Biosciences). The sample termed “femaleONT” was self-corrected by CANU (V2.0) after removal of raw ONT reads with average base quality ˂7.

#### Screening of female-specific-17-mers

A pipeline for W-17-mers was constructed to extract the W-reads separately for assembly (Supplementary Fig. 1a).

1. Random 17-mer sets were obtained with Jellyfish (Marçais and Kingsford 2011) using all type data, which included femaleNGS, maleNGS, femaleHIFI, and femaleONT. The 17-mers from sequence errors, which were identified by the *k*-mer frequency (Li et al. 2018a), were deleted before the next step.
2. A core female 17-mer set (femaleNGSHIFIONT) was generated by screening for 17-mers which were commonly present in all 3 *k*-mer data sets.
3. A core male 17-mer set (maleNGSHIFIONT) was generated by screening for 17-mers common to all 3 male *k*-mer sets, maleNGS, femaleHIFI and femaleONT data.
4. Female-specific-17-mers which only existed in femaleNGSHIFIONT, were screened by comparison between femaleNGSHIFIONT and maleNGSHIFIONT.

#### Screening of female-specific HIFI reads and contig assembly

The female-specific-17-mer set was mapped onto the HIFI reads using Bowtie2 (v2.3.4.3) with the following parameters: end-to-end -k 500 –quiet -p 2 -L 17. Only perfect matches were used in the next step. Then, the HIFI reads containing female-specific-17-mers of at least 3 kb or 100 kb per read were retained. The female-specific HIFI reads were assembled by CANU (v2.0) using the following parameters: -minOverlapLength=1000, -minReadLength=5000, -corOutCoverage=80 “batOptions=-dg 3 -db 3 -dr 1 -ca 500 -cp 50.” This assembly was named a “primary contig.”

#### Contig extension to cover the whole chromosome using HIFI and ONT reads

First, the contigs were extended by HIFI. The HIFI reads were mapped onto the “primary contig” using Minimap2 (v2.17) (Li 2018). The parameters of Minimap2 were as follows: -k17 -w11 -g8000 –max-chain-skip 5 -r500 -m8000 -A1 -C9 -O6,26 -B4 -E2,1 -s200 -z200 -N5 –min-occ-floor=500. Then, HIFI reads mapped within the 50-kb region at each end of a primary contig were extracted for assembly using criteria of overlap length ≥8 kb and identity ≥80%. The HIFI reads extracted from each end were assembled separately by CANU v2.0. The “primary contig” was extended if the homologous region between it and the new assembly reached 80 kb and ≥94% identity. The extended contig was used as a reference contig in the next round extension until the contig could not be extended.

The ONT reads were then used for extension of contigs in the same way. The ONT reads were mapped onto the reference contig using Minimap2 (v2.17) with the following parameters: -k17 -w9 -g20000 –max-chain-skip 6 -r500 -m10000 -A1 -C9 -O6,26 -B4 -E2,1 -s200 -z200 -N5 –min-occ-floor=500. The ONT reads mapped onto the 200-kb region near the end of a contig with the criteria of the overlap length ≥25 kb and identity ≥80% were extracted for assembly. The ONT reads extracted from each end were assembled separately by CANU v2.0. The “primary contig” was extended if the overlapped region between the new assembly and the “primary contig” and newly assembled contig met the following criteria: overlap ≥100 kb and identity ≥92%. The newly extended contig was used as a reference in the next round until the assembly reached a complete chromosome which included telomere repeats [(TTAGG)*_n_*] at both ends.

#### Correction of assembly errors by HIFI and ONT reads

In general, assembly errors occur mainly by the incorrect connection of repeated regions. Native sequence reads of a genome can be used to judge the assembly quality if the reads can be correctly matched with the genome (or “full-length alignment” reads). The current alignment software always uses not only fully matched full-length reads, but also partially matched reads. This often causes alignment of reads onto another homologous region, which leads to misalignment. Therefore, a reasonable and effective method is to evaluate the assembly quality by the coverage of “full-length alignment” (perfect-mapped) reads. In the present study, we developed a method of PBD for evaluation of the assembly quality (Li et al. 2024). “Perfect-mapped” reads in this study are such that reads are only allowed to have a small number of nonmatching regions at both ends; that is, HiFi reads are only allowed up to 200 bp of nonmatching regions and ONT reads no more than 1 kb.

1. Ultra-long ONT reads (≥50 kb) were mapped onto the reference contig using Minimap2 (v2.28) with the following parameters: -k19 -w9 –max-chain-skip 8 -r 500 -g20000 -m18000 -A1 -C5 -O6,24 -B3 -E2,1 -s200 -z200 -N5 –min-occ-floor=500. If the coverage of “full-length alignment” reads ranged from 0.5–2.5× of the average depth of the whole chromosome, the assembly was considered correct; otherwise, it was judged to be an incorrect linkage which was corrected.
2. LABC: all the alignable reads close to 100 kb of the assembly error breakpoint were extracted and assembled de novo using CANU (v2.0); then, the assembled contig was compared with the original contig (Li et al. 2024). The assembly error region was replaced when the overlap of both ends reached 100 kb (consistency ≥0.92), or the overlap region reached 70 kb (consistency ≥0.95).
3. Finally, correction of small errors was accomplished using HIFI reads. The HIFI reads were mapped to the whole genome (autosomes, Z and W chromosomes) using Minimap2 (v2.17), with the following parameters: -a –secondary= no -k19 -w11 –max-chain-skip4 -r 500 -g20000 -m8000 -A1 -C5 -O6,24 -B3 -E2,1 -s200 -z200 -N5 –min-occ-floor=500. The output was then saved in BAM format. In this step, the autosomes and Z chromosomes were added as reference sequences mainly to reduce mapping errors by reads from homologous regions. Correction of structural errors used Inspector with default parameters (Chen et al. 2021); small errors used Pilon (v1.23) (Walker et al. 2014) as follows: –diploid –duplicates –minmq 40 –mindepth 7 –minqual 0 –threads 3 –fix snps,indels,gaps.

#### Evaluation

**Fig. 1.**
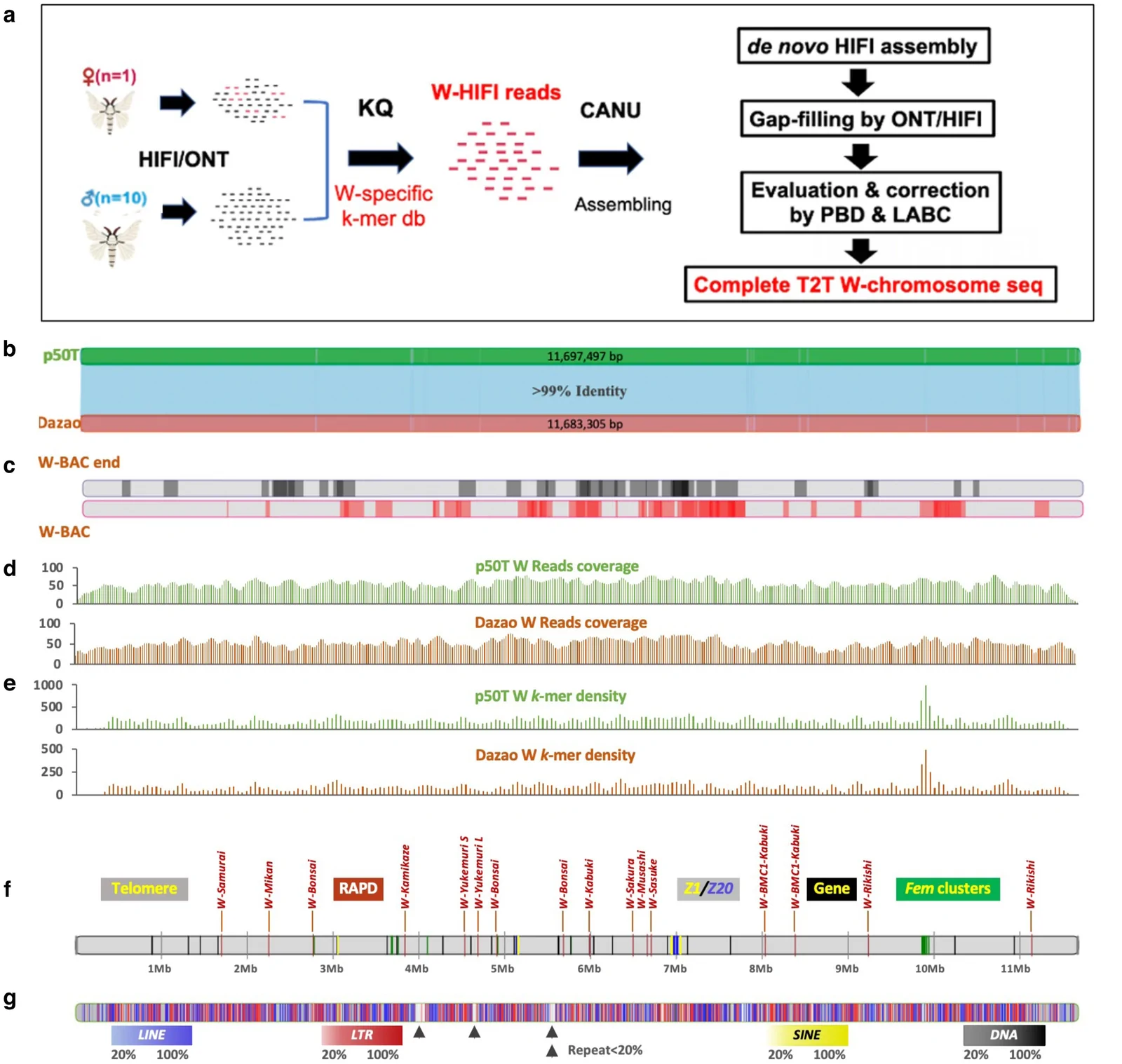
Sequencing and characteristics of the silkworm T2T_W chromosome. a) Schematic of assembling a complete T2T_W sequence. Left: The flow chart of procedures used to obtain W-HiFi reads from reads using a strict “*K*-mer Quotient” (KQ) method. Right: Pipeline of T2T W assembly. b) The identity between p50T_W and Dazao W. Regions with identity of more than 99% and less than 99% are painted with deep or light colors, respectively. The blue line in the middle links the corresponding regions of the 2 W sequences with identity ≥99%. Counts were made in a non-overlapping 1000 bp sliding window. c) Mapping of W-BACs on T2T_W. Red colored bars (lower set) represent mapping of sequenced W-BACs; black bars (upper set) show W-BACs for which both BAC-end sequences were mapped within 100–220 kb with consistent direction. The mapped W-BACs are listed in Supplementary Table 3. d) Coverage of p50T_W and DazaoW by mapped full-length ONT reads. Counts were made in a 50 kb sliding window with 25 kb steps. e) Distribution of W-linked 17-mers. The highest peak is located at the main *Fem* cluster domain. Counts were made in a 100 kb sliding window with 50 kb steps. f) Mapping of W-RAPD markers, *z1:z20* gene pairs and *Fem* clusters on Dazao W. brown, 12 W-linked RAPD markers; green, *Fem* clusters; yellow and blue, 4 pairs of *z1:z20*; black, other predicted protein genes; gray, telomere regions. g) Distribution of transposons and repetitive sequences on the W. For each continuous 5-kb window, only a dominant repeat-type is illuminated on the bar. The color scheme for each window is as follows: 1) When the rate of a certain repeat-type is higher than the other three by >5%, this window displays only the dominant repeat-type with the intensity representing the rate of this repeat (from light to dark representing 20%–100%). Blue, LINE; red, LTR; yellow, SINE; black, DNA transposons. 2) When the total repeat rate in a 5-kb window is <20%, the window is white (arrowhead). 3) The remaining windows are displayed in gray.

1. Female-17-mer distribution: female-17-mers obtained in the previous step were used to map onto the reference sequence using Bowtie2 (v2.3.4.3) (Langdon 2015) with the following parameters: –end-to-end -k 500 –quiet -p 2 -L 17; only identical *k*-mers were used. The average number of *k*-mers per 50 kb sliding window (step-size 25 kb) on the W chromosome was recorded (Fig. 1d).
2. Read coverage: ONT/PacBio HIFI reads were aligned to the assembled W sequence using Minimap2 (v2.17), and the coverage of “Perfect-mapped” reads on the reference sequence was calculated. HIFI read mapping parameters: –secondary= no -k19 -w9 –max-chain-skip 8 -r 500 -g20000 -m5000 -A1 -C5 -O6,24 -B4 -E2,1 -s200 -z200 -N5 –min-occ-floor=500. ONT reads mapping parameters: –secondary= no -k19 -w9 –max-chain -skip 8 -r 500 -G20000 -M10,000 -A1 -C5 -O6,24 -B3 -E2,1 -s200 -z200 -N5 –min-occ-floor=500. Read coverage on the W chromosome was counted per 50 kb sliding window (step-size 25 kb) (Fig. 1d).
3. Dazao and P50T W homology assessment: The 2 sequences were aligned using MAFFT (Katoh and Standley 2013) and then the identity ratio in consecutive 1000 bp windows was counted. Consecutive 1000 bp windows with identity more than 99% were merged.
4. W-specific BAC matching rate: Minimap2 (v2.17) was used to realign the BAC sequence to the reference sequence with the following mapping parameters: -k19 -w11 -g8000 –max-chain-skip 8 -r500 -m5000 -A1 -C9 -O6,26 -B4 -E2,1 -s200 -z200 -N5 –min-occ-floor=500. A BAC was considered to be fully supported when the matched BAC fragment distribution on the reference was between 80 and 250 kb (Fig. 1c).

### Silkworm BAC libraries and screening of W-BACs

Three BAC libraries derived from a mixed sex p50T strain constructed by partial digestion with *Eco*RI, *Hin*dIII, or *Bam*H1 are available as follows: *Eco*RI-BAC (Koike et al. 2003), *Hin*dIII-BAC (Wu et al. 1999), and *Bam*HI-BAC (purchased from the laboratory of Plant Genomics and GENEfinder Genomic Resource of Texas A&M University, and now a copy of *Bam*HI-BAC is stored in Southwest University). From these 3 BAC libraries, 81,024 × 2 BAC-end sequences were downloaded from KAIKObase [https://sgp.dna.affrc.go.jp/KAIKObase/] (Suetsugu et al. 2007). To identify W-BACs, we adopted the following 3 strategies:

1. KQ method + BAC-end sequences: BAC-end sequences produced by the Japanese National Institute of Agrobiological Sciences were downloaded from KAIKObase (https://sgp.dna.affrc.go.jp/KAIKObase/). We identified W-derived BACs based on these 137,219 BAC-end sequences followed by the KQ method (Li et al. 2018a). W-specific *k*-mers (*k* = 15) identified by the KQ method were used as markers to screen candidate W-derived BAC-end sequences. The corresponding BACs were considered W-derived candidates and used for analysis by PacBio sequencing.
2. KQ method + 3D-Pool: Based on de novo assembly of female genome NGS data, 60 W derived contigs were identified by the KQ method (Li et al. 2018a). Specific primers of these W-derived contigs were designed and used to screen the W-derived BACs in a *B. mori* 3D-Pool DNA library using a systematic PCR-based procedure (Wu et al. 1999).
3. W-RAPD markers + 3D-Pool: The 11 W-derived RAPD markers which were identified by Abe et al. (2005) were used for screening W BACs in the 3D-Pool DNA library.

### Construction of a 3D-pool for the silkworm *Eco*RI-BAC library

As the *Eco*RI-BAC library contained a large number of clones (149,456 BACs in 384 384-well plates), we constructed a 3D-Pool of a duplicate copy of the library in new 384 well plates for easy screening by PCR (Wu et al. 1999). Overnight cultures were then transferred to 96-well plates in a column and row-wise manner to obtain individual pools followed by transfer of an entire plate to a plate pool and DNA isolation using a PI-1200A Nucleic Acid Extractor (KURABO Industries Ltd, Japan). Plasmids from column and row pools were then isolated by mini prep and aliquoted to 96-well plates. Both high concentration stock plates and 1/20-diluted working plates were prepared.

### Annotation of the W assembly

The W chromosome is dominated by transposons, but a large number of coding genes can still be annotated by homologous annotation (data not shown). In order to obtain a more accurate set of W-linked genes, this study adopted the following strategy to predict coding genes based on RNA-seq data from Silkbase.

1. Construct transcripts using RNA-seq data. We used HISAT2 (v2.1.0) (Kim et al. 2019) to map embryonic RNA-seq data to the T2T genome, using the following parameters: –dta -p 2 –phred 33 –sensitive –no-discordant—no-mixed -I 1 -X 1000. RNA-seq data were obtained from Silkbase (Sequence Read Archive: DRR013821, DRR013822, DRR013823, DRR013824, DRR013825, DRR013826, DRR013827, DRR013828, DRR015667, and DRR015668).
2. Merge transcripts. Transcripts with identical exons in different samples were merged. Because RNA-seq data were prone to lose the ends, transcripts with differences within 50 bp of the end positions were merged and the longest transcripts were preserved.
3. Coding ability prediction. The encoding power of all transcripts was predicted using TransDecoder (v5.6.0). Transcripts with coding energy were classified as gene coding, and the rest were classified as noncoding RNAs. The longest one was retained when more than 1 transcript was identified in the same location.

### 5′ and 3′ RACE experiments of *z20* genes

Total RNA was extracted with TRIzol reagent (Tiangen, China) from pooled samples of fifth instar day 1 ovaries or testes performed according to the manufacturer's instructions. 5′ and 3′RACE ready cDNA was generated using a SMARTer RACE 5′/3′ Kit (TAKARA, Japan), according to the manufacturer's instructions. RACE PCR was performed with SeqAmp DNA Polymerase (TAKARA, Japan). The thermal cycler program was set as recommended by the manufacturer (Program 1 with the annealing temperature of the last 20 cycles set at 65 °C). After separation of samples by standard gel electrophoresis, visible bands were cloned using a pEASY®-T&B Zero Cloning Kit (TransGen Biotech, China) and analyzed via Sanger sequencing. The primers used in this experiment are listed in Supplementary Table 6.

### Fluorescence in situ hybridization mapping on chromosomes of yCsW females

Spread pachytene chromosome bivalents were prepared from the ovaries of last instar larvae of yCsW mutant females as described in Yoshido et al. (2015). Two DNA probes were mapped by fluorescence in situ hybridization (FISH) on the chromosome preparations: (i) the fluorescein-labeled W-BAC 19L6H probe was used to visualize the ancestral part of the mutant W chromosome (Sahara et al. 2003), and (ii) a Cy3-labeled 2-kb fragment of the *z1* gene was used to localize the gene on the W chromosome. The W-BAC 19L6H clone was cultured in LB medium containing 20 µg/ml chloramphenicol at 37 °C for 16 h, and BAC DNA was extracted with a Plasmid Midi kit (QIAGEN, Tokyo, Japan). The 2-kb *z1* fragment was obtained by PCR amplification of the *z1* sequence using W-BAC 039L08 (Supplementary Table 3) and *z1* primers listed in Supplementary Table 6. The PCR product was purified using a NucleoSpin Gel and PCR clean-up kit (TAKARA, Japan), cloned into a TA vector (TAKARA, Japan), and verified by ABI 3,700 Sanger sequencing. Probe labeling and FISH were performed according to the methods described in Yoshido et al. (2015). After FISH, preparations were counterstained and mounted with Vectashield Antifade Mounting Medium containing DAPI (Vector Laboratories, Burlingame, California). The FISH preparations were examined under a Leica DM6000B fluorescence microscope, and images were captured using a DFC350FX black and white charge-coupled device camera (both Leica Microsystems). The images were processed with Adobe Photoshop, Version 7.0 (Adobe Systems, San Jose, CA, USA).

### Phylogenetic analysis

We used *B. mori z1* and *z20* sequences to conduct BLAST homology searches in the NCBI BLAST database. Protein-coding sequences of homologous genes (minimum e-value 1.0e-5) were collected from GenBank and used for further analysis. A first step alignment was conducted with MAFFT software (v7.487) (Katoh et al. 2019). After obtaining alignments, we removed the sites in each alignment shared with <50% of the sequences. Then, we constructed a phylogenetic tree using IQTree2 (Ly-Trong et al. 2023). The second step was alignment by PRANK (v.170427) software (Löytynoja 2021) at a codon level, using a tree obtained in the first step as a guide. To obtain the final alignment, we removed the sites in each alignment that were shared by fewer than 50% of the sequences. Estimation of coalescent time was conducted with BLAST (v1.10.4) software (Camacho et al. 2009). We used a coalescent constant model to conduct the analysis assuming a strict molecular clock. We used HKY + Gamma + I mode (Hasegawa et al. 1985; Shoemaker and Fitch 1989; Yang 1994) for nucleotide substitution; first + second codon and third codon positions were distinguished during the calculation (Baele et al. 2011). We confirmed all parameters in the calculation had effective sample size larger than 200. The time tree figures were made using FigTree software (v1.4.4, http://tree.bio.ed.ac.uk/software/Figtree/).

## Results

### W chromosome sequencing and evaluation

We first used the KQ method (Li et al. 2018a) to obtain W-linked reads. By intersecting NGS, PacBio high-fidelity (HiFi) reads, and self-corrected ONT reads (Supplementary Table 1), we obtained 831,332 female-specific 17-mers for the W chromosome sequence of Dazao, the first silkworm strain used to construct a full-length genome sequence (Fig. 1a) (The International Silkworm Genome Consortium 2008). Alignment of female-specific *k*-mers to HIFI reads resulted in selection of 31,673 female-specific HIFI reads (Supplementary Fig. 1a). We followed this by generating a primary assembly. We generated an interaction map using Hi-C data that integrated the autosomes and the Z chromosome (Li et al. 2018a), revealing that only 9 contigs originated from the W chromosome; the remaining non-W-contigs were homologous to autosomes and the Z chromosome and exhibited weak interactions with the 9 W-specific contigs (Supplementary Fig. 1b). We used HIFI and self-corrected ONT reads to extend the W-contigs to fill gaps into a single chromosome, including a complete telomere sequence (TTAGG)*_n_* in both ends. Next, we developed the PBD method for a quality check and the LABC method to correct errors in assembly (Fig. 1a and see Materials and methods) (Li et al. 2024). Finally, we obtained a complete Telomere-to-Telomere (T2T) silkworm W chromosome sequence of 11683305 bp (T2T_W) (Fig. 1b; accession number SAMN60634153 in NCBI).

**Fig. 2.**
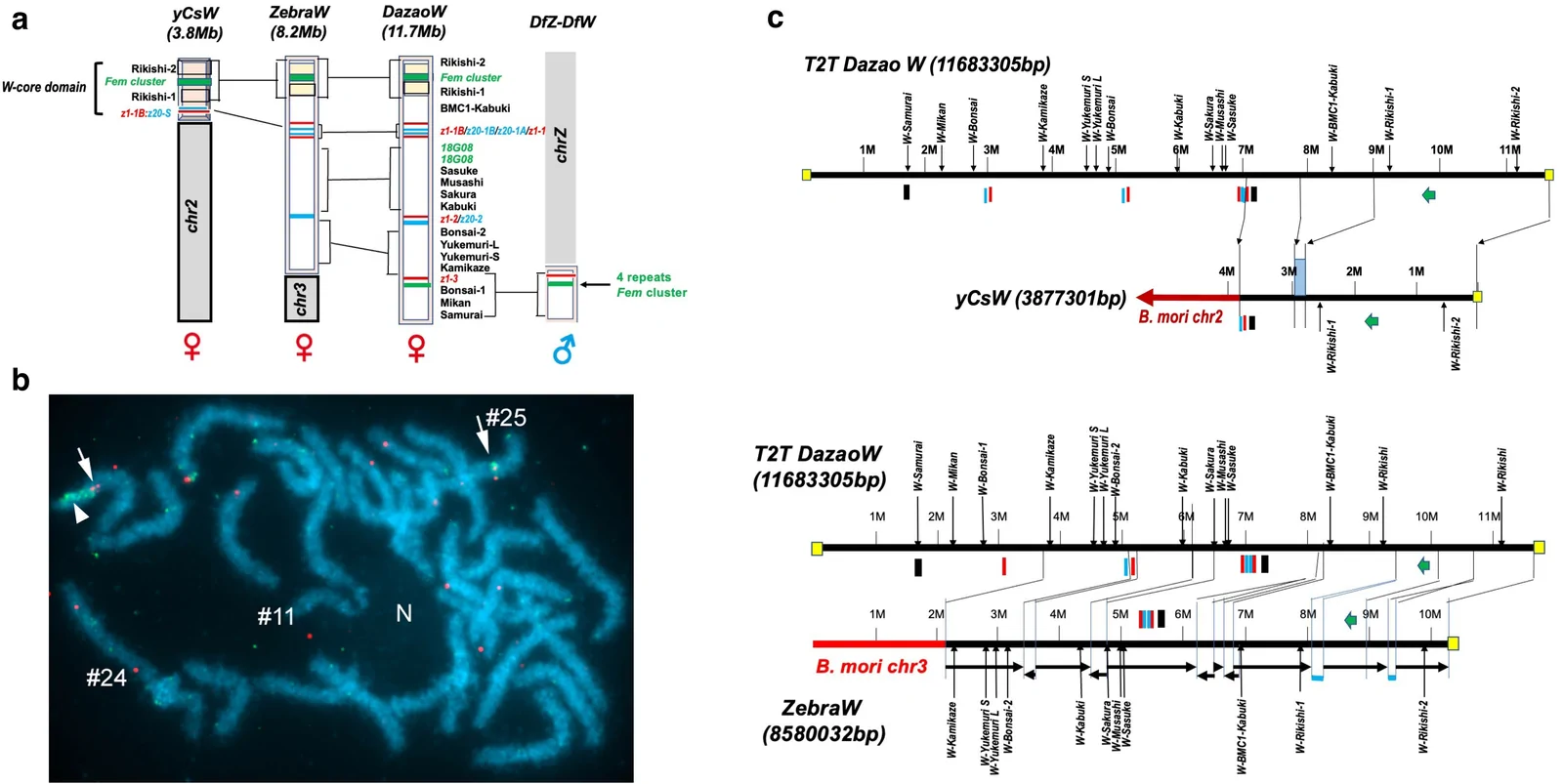
Evaluation and gene annotation of the T2T_W sequence. a) Distribution and correspondence of W-markers and genes on DazaoW and W-mutants. b) FISH image of pachytene chromosomes in the yCs mutant female. Green, the transposon-rich W portion of the W-chr2 translocation painted by the fluorescein-labeled W-BAC 19L6H probe; red, *z1* gene mapped with a 2 kb *z1* Cy3-labeled fragment as probe. A pair of red signals (arrow) observed at the boundary of the W and chr2 perfectly matches with the yCsW sequence. The Cy3-labeled *z1*-probe also mapped the *z2* gene (arrow) located on chromosome 25 (Supplementary Table 5; ID >97% with z1-CDS). To map the *znf-2* (=*z2*) gene located on chromosome 25, we also used a fluorescein-labeled BAC_1P06 probe, which covers the *znf-2* gene on chromosome 25, providing a strong green signal on #25 (arrow). #11, 24, 25, chromosome numbers; N, nucleolus; arrowhead, sex chromosome bivalent formed by pairing of yCsW and Z chromosomes, where the green part is the W-core domain (3.8 Mb) fused with a part of chromosome 2 (see Fig. 2c). c) Correspondence of T2T_DazaoW with yCsW (upper panel) and Zebra-W (lower panel). Yellow boxes at both ends denote telomeres. Arrows, main *Fem* cluster; vertical bars (red and blue), *z1:z20* gene pair; filled vertical bars, *SRRM1-like* gene *Bmor18276*; filled arrows, W-linked molecular markers. The positions are approximate.

To evaluate the assembly of T2T_DazaoW, we performed the following procedures: (i) we independently sequenced the native W chromosome of the p50T strain as described for Dazao (see Materials and methods). A comparison between the two W assemblies (Fig. 1b) showed an identity of more than 99% in 99.3% of the region with no long-range mismatch. (ii) For evaluation of the long-range assembly, we compared the T2T_Dazao W assembly with the eleven previously genetically mapped W-RAPD markers (Abe et al. 2005) and several W-deletion mutants produced by X-ray irradiation (Abe et al. 2008) (Fig. 2a). We sequenced the mutated W chromosomes of the sex-limited yellow cocoon (yCsW) and sex-limited Zebra-W (Zebra-W) mutants in the same way as the Dazao W (Fig. 2c; Materials and methods). Both sequencing and PCR methods provided an exact correspondence for W-RAPD markers, indicating that T2T_DazaoW had no long-range misassembly (Fig. 2a; Supplementary Table 2). (iii) We mapped 76 W-derived BAC clones (W-BACs) onto T2T_DazaoW, confirming the short-range accuracy (up to around 200 kb) of the W sequence (Fig. 1c; Supplementary Table 3). Finally, (iv) we mapped full-length ONT reads onto the T2T_W sequence (Fig. 1d) to confirm no misassembly and high-quality at >95% nucleotide level of the T2T_DazaoW. The W-specific 17-mer distribution indicated that the entire T2T_DazaoW sequence was linked to the W chromosome (Fig. 1e).

We compared the 3 previously reported complete sequences of the silkworm W, termed W-Lee (Lee et al. 2024), W-Han (Han et al. 2024), and W-KA (Wan et al. 2025) with the T2T_W reported here. For W-Lee, we found 2 *B. mandarina*-specific W-Yamato RAPD markers in the sequence (Abe et al. 2002), but almost no *B. mori* W-RAPD markers except for 5 W-Samurai and 1 W-Yukemuri-S among 11 W-RAPDs previously reported as having been mapped (Abe et al. 2005) (Supplementary Fig. 2a). Thus, we confirmed that the W-Lee sequence was derived from the W chromosome of Japanese *B. mandarina*, and the published W-Lee sequence originated from the *B. mori* p50ma strain contaminated with Japanese *B. mandarina* (Sakado strain) (Lee et al. 2024; see Supplementary Discussion 1). Although each unit of the *Fem* clusters was highly homologous to the others throughout the 2 W sequences, there was also an obvious difference in the *Fem* distribution between them as shown in Supplementary Fig. 2a. Thus, we infer that if the W-Lee sequence were derived from *B. mandarina*, it would have undergone massive sequence accumulations and deletions to erase most traces of such events.

Next, we compared our new T2T_DazaoW assembly by identity with W-Han (Han et al. 2024) (Supplementary Fig. 2b). The W-Han assembly is highly fragmented and its quality is considerably low. As evidence, in comparing the positions of W-RAPD markers (Abe et al. 2005) between the 2 sequences (Supplementary Fig. 2b) we found that 2 RAPDs, W-Musashi and W-Sasuke, were missing from the W-Han assembly. Alignment of other W-RAPD markers was also different from the new T2T_DazaoW assembly and from the reported genetic map (Abe et al. 2008), indicating that fragments in W-Han were incorrectly assembled.

Comparison with W-KA, W-L, and W-M from 3 previously assembled domestic strains (Wan et al. 2025) (Supplementary Fig. 2c) shows that the W-KA assembly was more complete than W-Han with a total length very close to Dazao_T2T, but the assembly is characterized by a fragmented and disorganized structure. This is clearly shown by the map of 11 W-RAPD markers, which is significantly different in terms of alignment from that of Dazao_T2T (Supplementary Fig. 2c). Thus, T2T_DazaoW presented here is not only the first complete W-chromosome sequence but also more accurately assembled.

### W features: high repeats, complete transposons, and high GC content

Combining homology and de novo prediction to annotate the whole genome of *B. mori* revealed a total of 61.6% repeats, wherein the W had the highest proportion of repeats (92.0%) at the chromosome level. In terms of superfamily abundance across the whole genome, LINE elements accounted for the highest proportion (39.6%), followed by DNA (15.0%), LTR (12.9%) and SINE (12.8%) elements (Supplementary Fig. 1d). The GC content of chromosomes (36.7%–40.4%) was positively correlated with the proportion of repeat sequences, whereas the GC content of the W showed a much higher value (47.5%) (Supplementary Fig. 1c).

The number of TEs on the W chromosome was much lower than on other chromosomes, although it had the highest proportion of repeats, indicating that it was enriched in full-length TEs as previously reported (Supplementary Fig. 1d) (Abe et al. 2005). LINEs accounted for the highest proportion (63.2%), which was nearly 1.5× higher than that of the whole genome (39.6%). LTR repeats followed (45.2%) with a proportion 3–4 times higher than the whole genome content (12.9%). In contrast to LINE and LTR elements, the proportions of DNA (5.5%) and SINE (1.6%) elements were significantly lower than the whole genome (15.0% and 12.8%). LTRs were significantly enriched on the W chromosome, which is consistent with the results reported by Abe et al. (2005). A similar tendency was reported for the W chromosome of the willow beauty, *Peribatodes rhomboidaria* (Geometridae), indicating that the 2 most abundant mobile elements, PRW LINE-like and PRW Bel-Pao, are also enriched in the W of that species (Hejníčková et al. 2023).

Furthermore, we found a W-specific 260 bp repeat sequence termed “Direct repeat 260,” which is derived from LTR-retrotransposon BmRT3 (Supplementary Fig. 3) (Jin-Shan et al. 2005). The copy number of the Direct repeat 260 amounted to over 1,300 in the W. Searching in Silkbase https://silkbase.ab.a.u-tokyo.ac.jp showed that the Direct repeat 260 encodes piRNAs, which would silence the activity of retrotransposons in germ lines (Supplementary Fig. 3a) (Siomi et al. 2011).

### Large-scale amplification and deletion in W-Rikishi RAPD marker sites

**Fig. 3.**
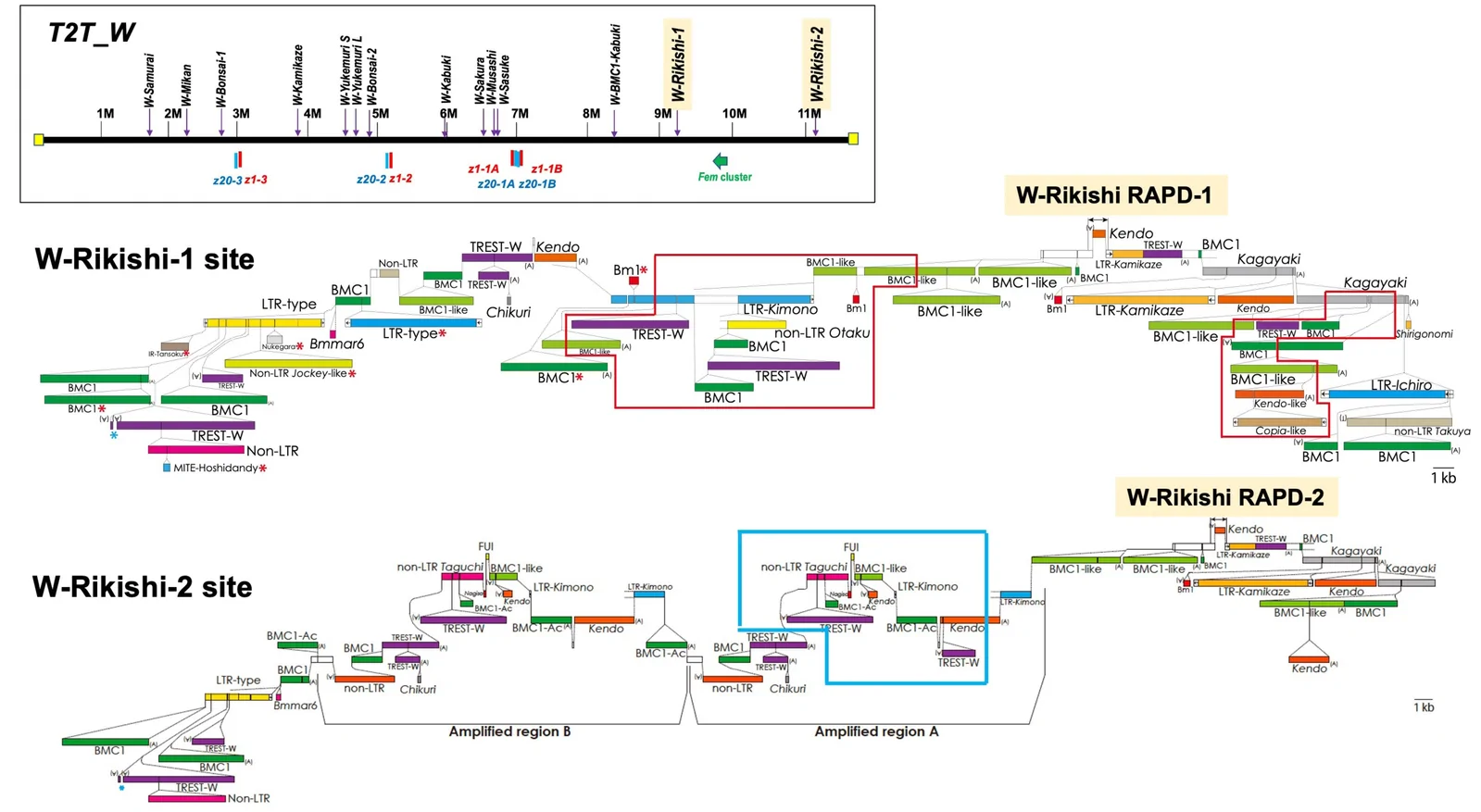
Model for large-scale amplifications and deletions found in 2 W-Rikishi RAPD sites. Originally a large region containing W-Rikishi RAPD-1 was amplified at a different site to become W-Rikishi RAPD-2. Subsequently, the boxed regions in the W-Rikishi-RAPD-1 site (red) were deleted from W-Rikishi RAPD-2 by intrachromosomal recombination between BMC1s. Then, an insertion of a complex nested structure composed of several TEs (enclosed by a box [blue]) around the 5′ LTR of Kimono in the W-Rikishi-2 site occurred to make a highly nested structure termed “Amplified region A.” Thereafter, gene duplication of “Amplified region A” occurred to make a tandem repeat labeled “Amplified regions A and B” in the W-Rikishi RAPD-2 site (see Supplementary Discussion 4). The TEs marked with an asterisk (red) located to the left of the W-Rikishi-1 site strongly suggests that this element inserted into W-Rikishi-1 after the W-Rikishi-2 region was duplicated using W-Rikishi-1 as a template. Furthermore, a series of tandem repeats containing poly(A) sequences (indicated by light blue asterisks) was identified at the left end of both regions.

The T2T_W sequence revealed 2 W-Rikishi RAPD marker sites, named W-Rikishi-1 and W-Rikishi-2, at different locations (Fig. 1f; Supplementary Table 2). Since the W-Rikishi RAPD marker is a complex nested structure composed of 3 TEs (Abe et al. 2005), it is improbable for insertions of identical structures to have occurred simultaneously at different loci. Comparing TE-nested structures of both W-Rikishi sites we can trace the evolutionary history of each site (Fig. 3; see Supplementary Discussion 4). First, a large domain containing W-Rikishi-1 was amplified to form W-Rikishi-2 at a different locus of the W, followed by deletion of the domains of W-Rikishi-1 from W-Rikishi-2 (enclosed by red lines in Fig. 3). Then, the amplified domain A (enclosed by blue line in Fig. 3) was inserted in the W-Rikishi-2 site, followed by a tandem duplication to form the amplified domain B in W-Rikishi-2. Thereafter, several TE insertions may have occurred in each W-Rikishi RAPD site (Fig. 3; see Supplementary Discussion 4).

### Annotation of W-linked genes

**Fig. 4.**
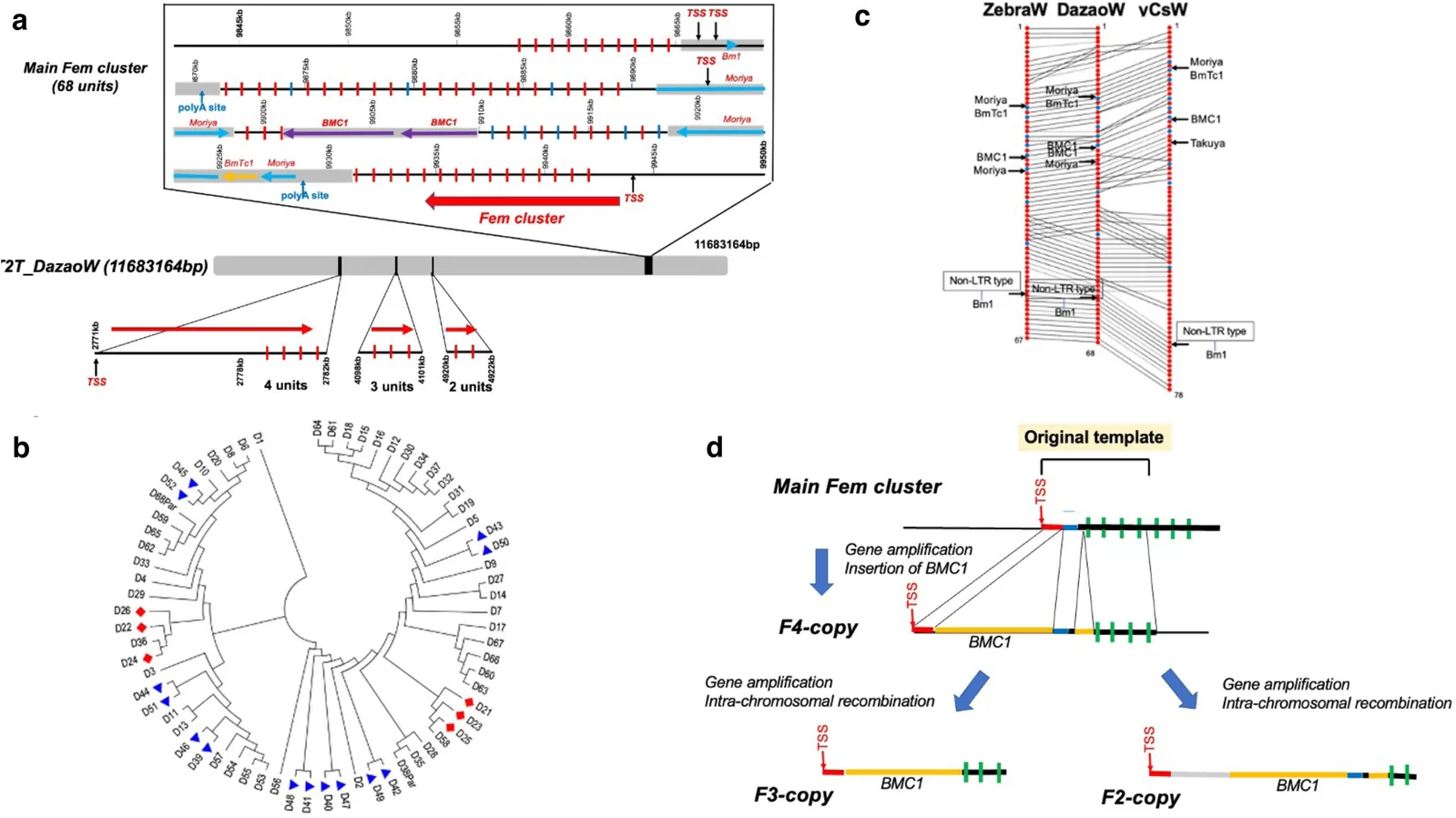
Distribution and structure of *Fem* clusters on the W. a) *Fem* clusters on the W. Light blue bars are noncanonical forms of 29 nt *Fem* piRNA containing a 1 nt difference from the canonical form (red bar). b) Phylogenetic tree analysis of 68 *Fem* units in the main *Fem* cluster in Dazao W. Among units 21–26 (red diamonds), unit numbers 21, 23, and 25 are canonical *Fem* piRNAs, whereas units 22, 24, and 26 are noncanonical, indicating unit pair 21–22 amplified to form the current alignment of units 21–26. Similarly, the domain of units 39 to 45 (blue triangles) was amplified to form unit 46 to 52 (blue triangles). c) Correspondence between *Fem* units of the *Fem* clusters of Zebra-W, Dazao-W, and yCs-W. Units showing >97% identity between the 2 main clusters are connected by straight lines. Arrows, insertions of TEs. d) Small *Fem* clusters formed from the 5′ portion of the main *Fem* cluster produced after gene amplification events by intrachromosomal recombination. The green bar denotes a *Fem* unit.

Annotation of T2T_W revealed 4 *Fem* clusters on the W. The largest of these clusters consisted of 68 units of ∼750 bp including the 29 nt *Fem* piRNA sequence, together with 3 other small clusters composed of 2–4 repeat units (Fig. 4a). Phylogenetic analysis of the largest *Fem* cluster revealed a composition of 68 units formed by domain amplifications (Fig. 4b; Supplementary Discussion 2). We compared the main *Fem* clusters of 2 other W-mutants, Zebra and yCs, with that of Dazao (Fig. 4c), and found differences among the 3 strains in the number of units in the main cluster, which would have been caused by repeated cycles of amplifications and deletions. Since a minimum number of *Fem* units must be essential for female determination by the piRNA pathway (Kiuchi et al. 2014), *Fem* piRNAs damaged by mutation and transposon attack would be removed and new *Fem* units would be amplified to keep a functional number of *Fem* units in the cluster, perhaps similar to mechanisms attributed to preservation of telomere repeats and rDNA units (Kobayashi 2011). Sequence analysis showed that the transcriptional start site (TSS) and 5′ regulatory domain sequences of these 3 small cluster genes were shared with those of the main *Fem* cluster, suggesting these 3 small clusters were formed from the main cluster by amplification and intrachromosomal recombination (Fig. 4d; Supplementary Discussion 2).

**Fig. 5.**
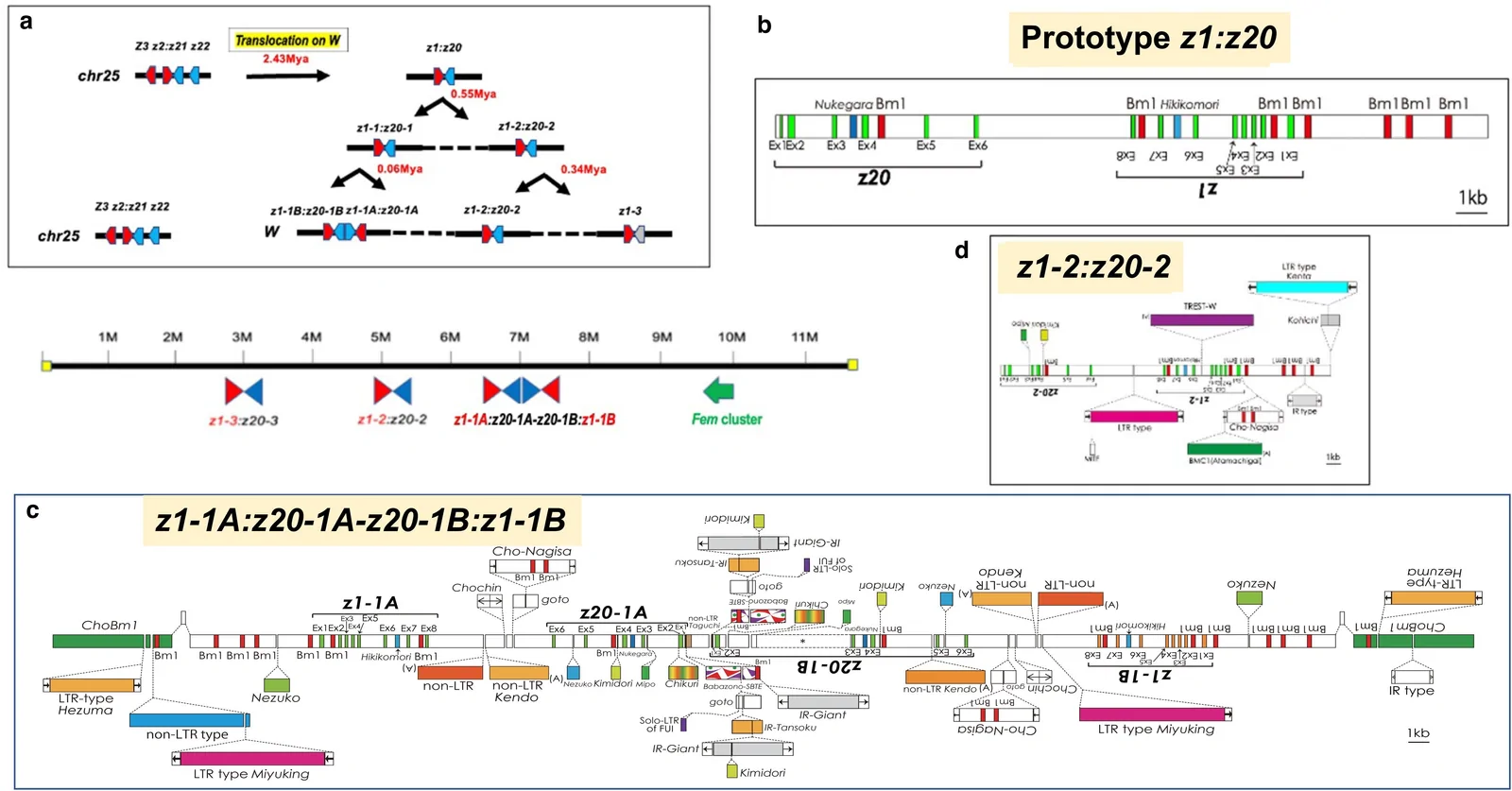
Evolution and structures of 4 *z1:z20* gene pairs. a) A schematic evolutionary map showing the distribution of *z1:z20* pairs on the W and chr25. Phylogenetic analysis of zinc-finger motif genes (Supplementary Fig. 4) describes the evolutionary process. *z1-1A:z20-1A-z20-1B:z1-1B* are symmetrically arranged and transcribed; the other 2 pairs are pseudogenes. b) Prototype of the ancient *z1:z20* structure. After removing differential TEs among 4 *z1:z20* gene pairs, the ancient *z1:z20* gene structure is considered as a prototype, which contains 7 Bm1s. c) Structure of symmetrically aligned *z1-1A:z20-1A-z20-1B:z1-1B*. d) Structure of a *z1-2:z20-2* gene pair.

To identify protein-coding genes on the W, we used RNA-seq data obtained from Silkbase (https://silkbase.ab.a.u-tokyo.ac.jp) to construct a transcriptome database, then used TransDecoder (v5.6.0) to predict genes with coding ability (See Materials and methods). Finally, we obtained 26 protein-coding genes termed *Bmor18254-18279* (Supplementary Table 4; Supplementary Datasets 1 and 2). These included 4 encoding zinc fingers, among which 16 genes were identified as TEs by searching in a *B. mori* transposon database BmTEdb (Xu et al. 2013) (Fig. 1g; Supplementary Table 4; Supplementary Datasets 1 and 2). We also found 4 *z1* and *z20* zinc-finger motif genes on the W termed *z1-1a:z20-1a, z1-1b:z20-1b, z1-2:z20-2*, and *z1-3:z20-3*, which were paired in a tail-to-tail orientation (Figs. 1f, 2c, and 5; Supplementary Table 5). *z1* contained 2 CCCH-type zinc-finger motifs, whereas *z20* contained 6 C2H2-type motifs. Among the 4 pairs, 2 pairs, *z1-1a:z20-1a* and *z1-1b:z20-1b,* were aligned symmetrically in the domain [T2T_W:6946455..7046150] (Fig. 5). *z20-3* had lost exon 1 and 2 of *z20* and was a pseudogene.

**Fig. 6.**
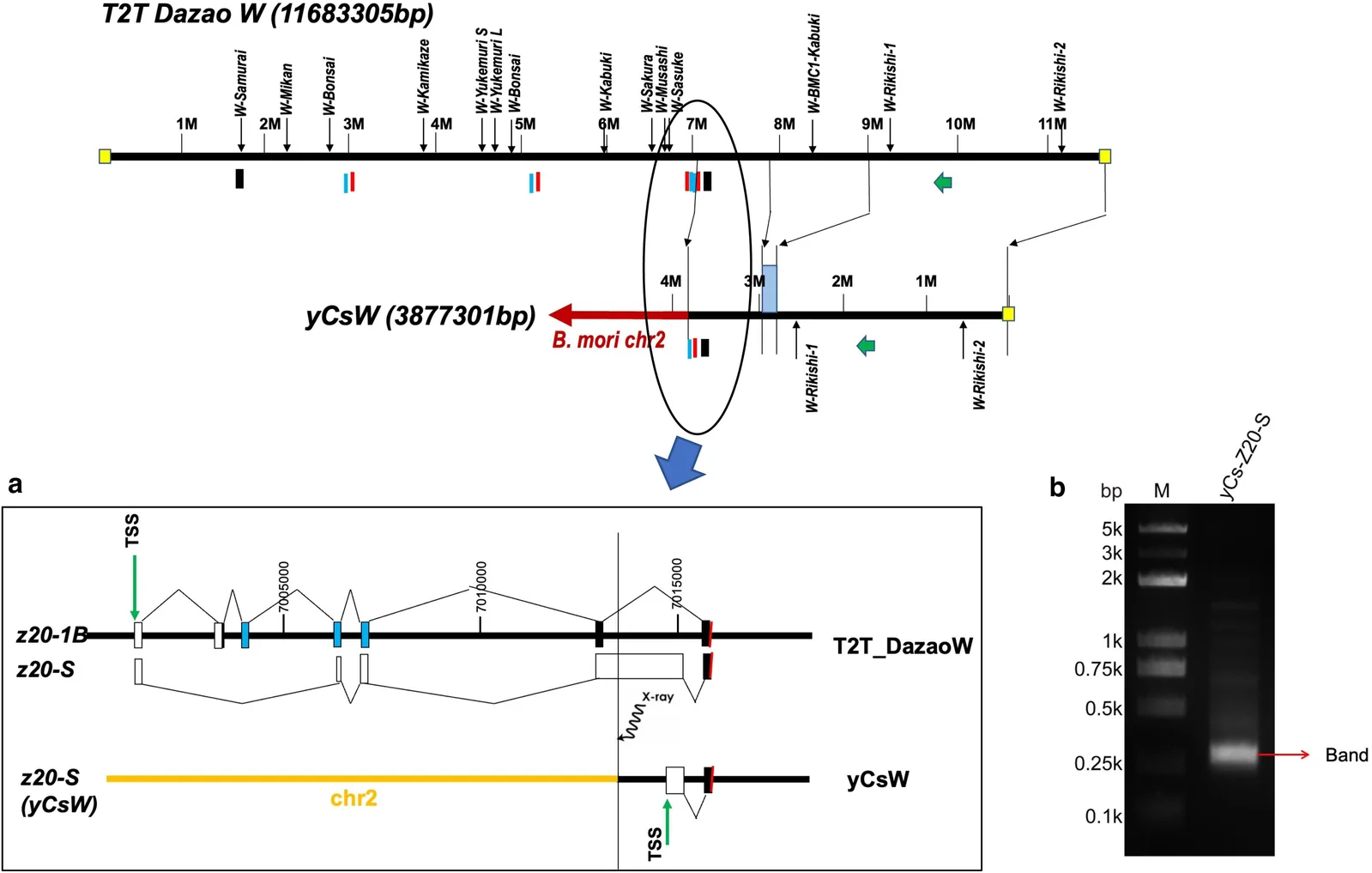
Structure and transcription of the *z20* and *z20-S* genes in DazaoW (D9L) and yCsW. Top: Correspondence between Dazao W and translocated yCsW chromosomes showing sites of zinc-finger genes. Translocation between chrW and chr2 in yCsW occurred at the *z20* gene of a *z1-1B:z20-1B* gene pair, resulting in the removal of the domain from the TSS site to exon 5 of *z20* in yCsW. The positions are only approximate. a) Magnified structure circled in the top panel indicating alternative splicing of the *z20* gene in DazaoW and the *z20-S* gene in yCsW. b) 5′ RACE of *z20-S* in the yCs mutant to determine the TSS of *z20-S*. RNAs were extracted from yCs female eggs at 16-48 hpo.

Among the annotated protein-coding genes, we found *Bmor18258* (Supplementary Table 4) was shared with exon 6 of the *z20* gene, and was derived from an alternative splicing form of the *z20* gene, termed *z20-S* (Fig. 6a). Two *Bmor18276* genes were found on T2T_W: [1687583..1689708] and [7129979..7131698]; however, the CDS of the former gene was mutated to produce a stop codon in its exon/intron consensus sequence, producing a pseudogene (Fig. 2c). The transcribed copy of *Bmor18276* was located 110 kb apart from *z1-1B*. The encoded protein has homology with serine/arginine repetitive matrix protein 1-like (SRRM1-like) of *Ostrinia nublialis* (XP_063835249.1) (265aa, Ser 12.8%, Arg 12.4%).

### *z1-z20* gene pair originated from a translocated copy of the homologous pair on chromosome 25

A single site of chr25 also contained 2 pairs of zinc-finger motif genes homologous to *z1:z20* in the W chromosome, termed *z2:z21* and *z3:z22* (Supplementary Table 5). Phylogenetic analysis of these 6 gene pairs suggested that the ancient prototype W-linked motif gene pair originated from a translocated copy of the autosomal *z2:z21* pair to the W about 2.43 Mya. This was followed by 2 rounds of gene duplication to generate the 4 currently existing W-linked gene pairs (Fig. 5a; Supplementary Fig. 4).

Figure 5 shows that the domains surrounding the *z1:z20* gene pairs on the W are composed of highly nested TEs. Since many TEs have accumulated on the W due to lack of meiotic recombination, by comparing only TEs shown to be common to the 4 W-linked *z1:z20* gene pairs against those associated with the chr25-linked *z2:z21* pair, we hypothesize an ancient *z1:z20* prototype originated from a copy translocated onto the W about 2.43 Mya (Fig. 5b; see Supplementary Discussion 3). The *z1:z20* prototype containing 7 Bm1s (a SINE of *B. mori*) is considered a long retrotransposable element SBTE (Secondary Bm1 Transposable Element) (Fig. 5b) (Abe et al. 2010). This suggests the *z2:z21* unit linked to chr25 was transcribed into RNA and then inserted into the ancient W with reverse transcriptase to form *z1:z20*.

### W-core domain

To identify candidates for female sex-determination and differentiation-related genes on the W, we sequenced the shortest W (3877301 bp) of the yCs-female-inducing mutant (Figs. 2a, 2c, and 6). We found only 4 genes: a *Fem* cluster composed of 78 repeat units, a pair of *z1:*exon 6 of *z20* and an SRRM1-like *Bmor18276* gene. Sequencing of yCsW revealed that the X-ray irradiation originally used to produce this chromosome aberration had cleaved *z20-1B* to remove a segment which covered from the 5′ regulatory domain through exon 1-5 of *z20-1B*, retaining only *z1*:exon 6 of the *z20* pair (Fig. 6a). This hypothesis was corroborated with a FISH image of yCsW and confirmed by its sequence (Figs. 2b and 6). Therefore, we carried out 5′ RACE using RNA extracted from early embryos of the yCs mutant to determine whether or not it is transcribed as an isoform of *z20-1B*. We found a very similar transcript of *Bmor18258* which contained exon 6 of *z20-1B*. The TSS site was located on a sequence [yCsW:3807356] within intron 5 of the *z20-1B* of T2T_W, which we named *z20-S*. In addition, the yCsW sequence revealed that the gene *Bmor18276* was located 110 kb apart from *z1* (Figs. 2c and 6). Altogether the W-core domain of the yCs mutant contains 4 conventional genes: *Fem* cluster, *z1:z20-S* and *SRRM1-like* genes.

## Discussion

We have succeeded in generating a more complete and accurate sequence of the T2T_W chromosome of the silkworm *B. mori* than is currently available in recent publications. We verified the accuracy of the sequence by using unique silkworm bioresources including W-deletion and translocation mutants, genetically mapped W-molecular markers and W-BAC clones. The T2T_W sequence revealed that the W is composed of a massive 92% accumulation of transposons and repeat sequences, among which the main constituents are full LTR and LINE retrotransposons. As seen in the previously identified *Fem* cluster and the newly discovered zinc-finger motif gene pairs as well as 2 W-RAPD-Rikishi sites, large-scale deletions and amplifications have clearly played a central role in shaping the evolutionary trajectory of the W chromosome.

**Fig. 7.**
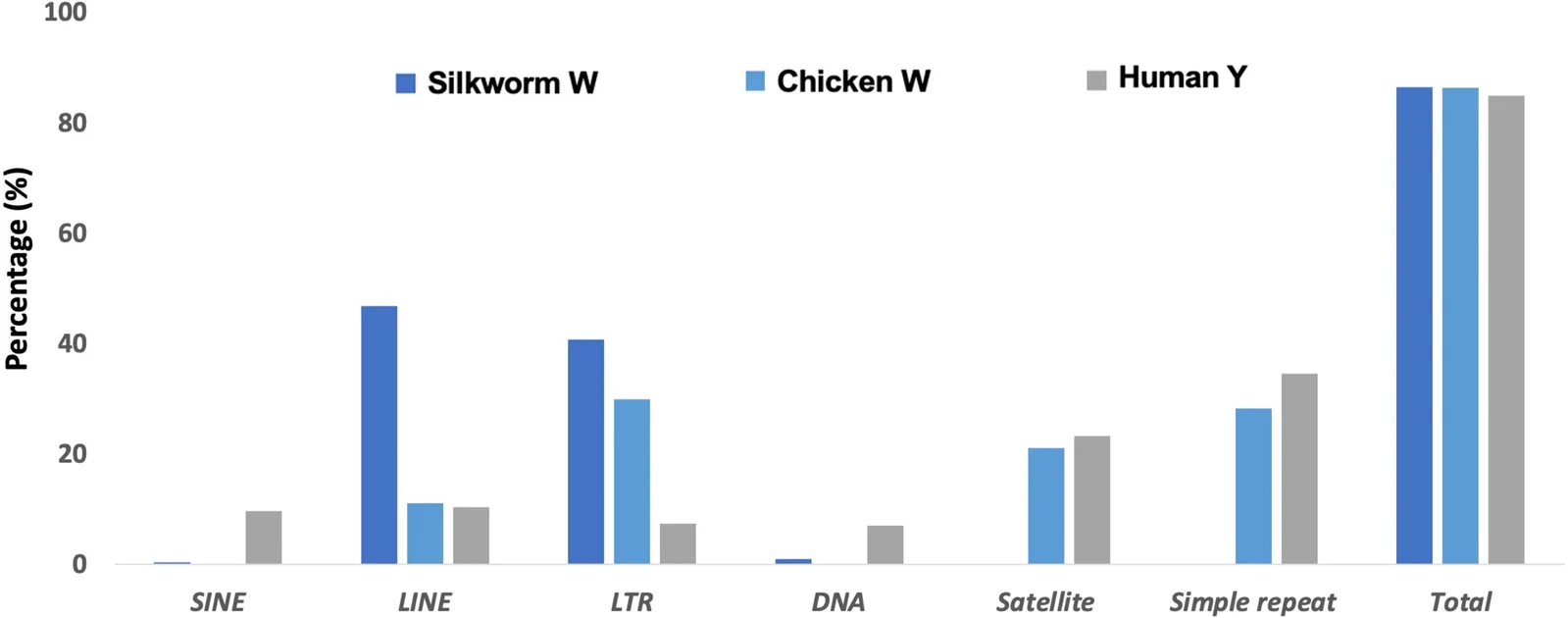
Comparison of LTR, LINE and simple repeat contents in the silkworm W, chicken W, and human Y.

Comparing the classification and proportion of repeat sequences on the silkworm W chromosome with those of the chicken W and human Y chromosomes, we found great differences in the types of repeat sequences (Fig. 7). The main repeating sequences on the silkworm W chromosome were LINE and LTR elements, whereas LTR and simple repeats are the main repeating sequences on the chicken W chromosome (Huang et al. 2023), and simple and satellite DNA repeats predominate on the human Y chromosome (Rhie et al. 2023). Significantly different from these species, the W chromosome of *B. mori* was also characterized by a large number of intact LINE and LTR retrotransposons. The high quantity of intact LINE and LTR repeat sequences imply that these W-linked transposable elements proliferated recently (Hejníčková et al. 2023; Lee et al. 2024). These results are also consistent with the inference that the Y chromosome in mammals (Cortez et al. 2014) and the W of birds (Xu and Zhou 2020) degenerated heavily in the distant past, and the W chromosome of the silkworm has undergone repeated long-range amplifications and deletions which significantly altered its DNA sequence even recently.

The present study also uncovered evidence which allowed us to determine whether the W has evolved from an autosome or the Z chromosome, a persistent research question about animal sex chromosome evolution. The heterochromatic avian Z/W chromosome pair was reported to have evolved from an autosomal pair (Zhou et al. 2014; Xu and Zhou 2020). Berner et al. (2023) reported collinearity between the W and Z chromosomes of *Pieris* butterflies, suggesting that the W has evolved from the Z chromosome in that species. Moreover, the largely or completely nonhomologous W and Z chromosomes of Lepidoptera pair during meiosis and form bivalents, which could imply a shared ancestry (Marec and Traut 1994; Sahara et al. 2003). However, if the W were derived from ancestral autosome pairs or from the Z, we would have found some sequence similarity between the genes on that specific chromosome and transcripts on the W (Furman et al. 2020; Lewis et al. 2021).

To study this aspect of the evolution of the silkworm W, we checked the alignable fragments of T2T_W, gene models and repeat units across autosomes and the Z chromosome (Supplementary Fig. 5a). The results showed the W had no collinearity to other chromosomes including the Z, and no biased homology to a specific chromosome. This observation is supported by the significant difference in the repeat contents of the W from autosomes and Z chromosome (Supplementary Fig. 1d). In addition, we found 4 of 26 T2T_W annotated protein-coding genes were shared with the Z chromosome; however, most of the 4 genes are transposons (Supplementary Table 4). We then mapped those 4 genes on the W and Z chromosomes (Supplementary Fig. 5b). These data showed no collinearity between the 2 chromosomes. Nor did we find any other evidence for synteny between the W and Z chromosomes, nor trace of a pseudoautosomal region as observed in the human Y (Rhie et al. 2023) and the avian W (Zhou et al. 2014; Xu and Zhou 2020). Although, again, frequent large-scale deletions and amplifications during evolution would have erased most traces of this kind of history from the current W sequence. These observations conform well to the report of almost no sharing of W-RAPD markers between the W chromosomes of *B. mori* and its ancestral species Japanese *B. mandarina* (Abe et al. 2005). Furthermore, unlike the human Y and chicken W, we found that the repeat sequences on the silkworm W comprise a large number of intact LINE and LTR retrotransposons (Fig. 7; Supplementary Fig. 1d). This suggests the silkworm W underwent massive proliferations of these retrotransposons recently.

Analysis of the evolution of 210 lepidopteran chromosomes by Wright et al. (2024) revealed that smaller chromosomes have a higher repeat density with higher GC content, and chromosome fusion events are biased by production of small, repeat-rich chromosomes and sex-linked elements. These characteristics significantly fit the patterns we observed on the silkworm W chromosome (Supplementary Figs. 1c and 1d). Accordingly, the silkworm W may have originated from the fusion of small chromosomes with high repeat and GC content along with sex-linked elements and massive proliferation of retrotransposons. However, there are no Merian elements or syntenic gene blocks corresponding to, for example, small autosomes missing from the silkworm genome compared with the inferred ancestral karyotype of Lepidoptera (Wright et al. 2024). There is also no evidence that the Z chromosome originated from a fusion with a small autosome whose homolog would have given rise to the W chromosome (Li et al. 2024; Wan et al. 2025).

If fusion between small chromosomes produced the ancestral silkworm W, traces of this process might be found in the current W chromosome. Wright et al. (2024) reported that GC content is higher toward the ends of chromosomes compared with their centers. Hence, we surveyed the T2T_DazaoW sequence for GC content and for the expected distal chromosome telomeric repeat TTAGG and telomere-specific non-LTR TRAS1 and SART1 elements (Okazaki et al. 1995; Takahashi et al. 1997). Wright's prediction was true for the silkworm W at both ends, in addition to which we found another high GC content domain of around 8.5–9.5 Mb (Supplementary Fig. 6). This domain contained SART1 [8839554..8863679; 9300314..9301227] and vestiges of telomeric repeats at [8585882..8586053; 8804517..8804572], although another telomere-specific LTR-retrotransposon TRAS1 was located only at the 2 ends. These observations support the alternative hypothesis that the silkworm W was formed by fusing small chromosomes with high GC and high repeat content with sex-related genes (Wright et al. 2024), although the possibility cannot be ruled out that the remnants of telomeric repeats and telomeric-specific transposons could have been transferred from the telomeres during proliferations by transposons. We also cannot exclude the possibility that, in addition to the GC-rich telomeric retrotransposons SART1 and TRAS1, other GC-rich repeats such as LINE and LTR elements (Fig. 7) accumulated randomly at the ends of the silkworm W chromosome. Since we did not find any significant traces of an origin from the Z chromosome or autosomes in the W chromosome sequence (Supplementary Fig. 5), it is worth considering the hypothesis that it arose from a supernumerary B chromosome, which has been favored by several studies (eg Dalíková et al. 2017; Fraïsse et al. 2017). Such a B chromosome could arise from a repetitive region of the genome during chromosomal rearrangements, begin to pair with the Z chromosome, and enlarge by accumulating additional repeats (Johnson Pokorná and Reifová 2021). In any case, it is quite difficult to trace the evolutionary trajectory of the silkworm W, as frequent large-scale amplifications and deletions on the W chromosome have erased most relics of evolutionary events.

Sequencing of the shortest W mutant, yCsW, revealed that among the large number of repetitive elements, the only conventional gene elements are the *Fem* cluster, a single pair of *z1:z20-S* protein-coding sequences and the SRRM1-like protein gene *Bmor18276* (Figs. 2c and 6). The z20-S and Bmor18258 proteins have very high sequence similarity with “protein Suppressor of hairy wing-like” (*Su(Hw)-like*) of several lepidopterans including *B. mandarina* (XP_028033922.1) (Supplementary Table 4), *Manduca sexta* (XP_037294017.1, XP_037294018.1), and *Pieris napi* (XP_047504424.1). In *Drosophila*, Su(Hw) is reported to play an essential role in development of the female germline and ovary and function in oogenesis (Baxley et al. 2011; Soshnev et al. 2013). The phylogenetic evidence indicates that the *z1:z20* gene pair originated from a copy translocated onto the W from a *z2:z21* gene pair on chr25 around 2.43 Mya (Fig. 5a; Supplementary Fig. 4). *z2* homologs containing 2 CCCH-motifs are found throughout Lepidoptera. Recently, Herran et al. (2022) reported that 2 newly isolated CCCH-type zinc-finger protein genes, which are homologs of *BmMasc* and *Bmznf-2* (=*z2*), are involved in male sex determination in *Ostrinia scapulalis*. Furthermore, Fukui et al. (2026) reported that Bmznf-2 interacts with BmMasc and enhances BmMasc-dependent masculinization. We also found an SRRM1-like protein gene, *Bmor18276,* in DazaoW and yCsW. The SRRM1 family is involved in pre-mRNA splicing and other RNA processes (Blencowe et al. 1998). Homologs of *Bmor18276* are widely distributed in Lepidoptera including *B. mandarina* (XP_028043394.1), *Helicoverpa zea* (XP_047026293.1), and *O. nubilalis* (XP_063835249.1).

Gene duplication, mutation, and translocation of sex-determination genes are reported to increase the variety of sex-determination systems in many species (Foster and Graves 1994; Yoshimoto et al. 2008; Sutton et al. 2011; Moalem et al. 2012; Takehana et al. 2014). While conducting this study in *B. mori*, we found translocation and duplication of the *z2:z21*pair onto the W chromosome. It was recently reported that the Bmznf-2 (= z2) protein is involved in the masculinization pathway in cooperation with BmMasc (Fukui et al. 2026). We hypothesize that the translocation of *z2:z21*onto the W chromosome would confer additional feminizing traits in this moth, which remains a challenge for future research.

## Supplementary Material

jkag170_Supplementary_Data

## Data Availability

All sequencing and assembly data for this study have been deposited in the China National GeneBank (CNGB) Sequence Archive (CNSA) database under accession number CNP0005783. The sequences for Dazao T2T_W (11,683,305 bp), p50T T2T_W (11,697,497 bp), Zebra-W (850,032 bp), and yCs W (3,877,301 bp) are available under assembly accession numbers CNA0140293, CNA0140294, CNA0140295, and CNA0140296, respectively. Sequencing data of W-BACs obtained in this work are available under accession number CNA0144055 within project CNP0005783. In addition, we deposited the sequence data of the silkworm W chromosome in the National Center for Biotechnology Information (NCBI) database. The sequences for Dazao T2T_W (11,683,305 bp), p50T T2T_W (11,697,497 bp), Zebra-W (850,032 bp), and yCs W (3,877,301 bp) are available under accession numbers SAMN60634153, SAMN60634809, SAMN60635291, and SAMN60634824. Sequencing data of W-BACs obtained in this work appear under accession number SAMN60635380. Requests for materials should be addressed to Hiroaki Abe (1313ywyc@jcom.home.ne.jp) and Kazuei Mita (mitakazuei@gmail.com). Supplemental material available at G3 online.
