# Supplementary material for: The complete sequence of the silkworm W chromosome uncovers its rapid evolution by large-scale duplications/deletions and translocation of W-linked genes
Source: G3 (Bethesda). 2026 Jun 30;16(9):jkag170. doi: 10.1093/g3journal/jkag170 (PMC13535481; doi:10.1093/g3journal/jkag170)
Supplement: Supplementary file 1 [file jkag170_supplementary_data.docx]

**Supplementary materials for**

**The complete sequence of the silkworm W chromosome uncovers its rapid evolution** **by large-scale duplications / deletions and translocation of W-linked genes**

Wanshun Li, Hiroaki Abe, Yue Chen, Jianqiu Liu, Yanjue Wang, Masahiro Ajimura, Shenglong Li, Yingdan Xiao, Huizhen Guo, Jiaqi Wu, Yajing Xu, Xue Yang, Xiaoxue Yang, Shaopeng Hao, Qihue He, Tsuguru Fujii, Ken Sahara, Atsuo Yoshido, Yusuke Takehan, Kiyoshi Naruse, Kallare P. Arunkumar, Marian R. Goldsmith, Guy Smagghe, František Marec, Qingyou Xia, Kazuei Mita

Corresponding authors: Correspondence should be addressed to HA, KPA, MRG, QX and KM (1313ywyc@jcom.home.ne.jp, arunkumar.kallare@gmail.com, mki101@etal.uri.edu, xiaqy@swu.edu.cn & mitakazuei@gmail.com).

**This file includes**:

Supplementary Figures 1–6

Supplementary Tables 1–6

Supplementary Discussion 1–4

Supplementary References

Supplementary Datasets 1 and 2

**Supplementary Figures 1–6**:

**Fig. S1.** Characteristics of the T2T_W structure. a) Pipeline for screening female-specific *k*-mers. b) Hi-C interaction map of all chromosomes. Numbers 1-28 correspond to the Z chromosome and autosomes. Number 29 is a mixture of the W chromosome and some fragments of autosomes and Z. A distinct Hi-C interaction which encompasses nine W-contigs is located in #29. The remaining fragments are homologous to autosomes and do not exhibit any interaction with W-contigs. c) Chromosome GC content. The GC content of the autosomes and Z chromosome is positively correlated with the percentage of repetitive sequences, whereas the W chromosome shows a much higher value. d) Distribution of transposable elements (TEs) in chromosomes. Upper panel: the number of TEs in each chromosome. Lower panel: the proportion of TEs.

**Fig. S2.** Comparison between the T2T_DazaoW assembly and three published W sequences.

a) Comparison with W-Lee (Lee *et al*. 2024). Brown bars show the positions of W-RAPD markers. W-Yamato denotes a *B. mandarina*-specific W-RAPD marker. Green bars indicate *Fem* clusters marked by the number of repeats at each site. Red arrows denote the direction of transcription. Eleven W-RAPD markers colored black were missing from the W-Lee assembly. b) Comparison with the W-Han assembly (Han *et al*. 2024). The same parameters were used for calculating identity as used between Dazao_W and p50T_W (Fig. 1e). In the W-Han assembly regions with higher identity are painted with darker color; those with lower identity with lighter color. The colored lines in the middle link the corresponding regions of both W sequences. The thick black bars in T2T_W denote the regions where no corresponding sequences were found in the W-Han assembly. In the homologous region at least 20 kb is illuminated. W-RAPD markers are labeled. Two W-RAPD markers colored black were missing from the W-Han assembly. c-e) Comparison with the W-KA, W-L and W-M assemblies (Wan *et al*. 2025). These three W assemblies are from three domestic silkworm strains with long-term pupal characteristics published by Wan *et al*. (2025). The same parameters were used as for W-Han. W-KA and W-L contain 11 W-RAPD markers, but their alignment is significantly disordered )compared to the current assembly. W-M contains 10 W-RAPD markers, lacking the Rikishi marker and this whole domain where two Rikishi markers (black colored) are located.

**Fig. S3**. Structure of W-specific Direct repeat 260. a**)** Sequence of Direct repeat 260. Searching in Silkbase <silkbase.ab.a.u-tokyo.ac.jp> indicates that the element encodes piRNAs (red and blue lines). b) Distribution of Direct repeat 260 in the W.

**Fig. S4.** Phylogenetic tree analysis of *z1:z20* and their homologs. The time tree figures were made using FigTree software (v1.4.4, http://tree.bio.ed.ac.uk/software/Figtree/) (see Materials and methods).

**Fig. S5.** Collinearity check between W and other chromosomes. a) Chromosomal distribution of homologous alignments of W gene models (blue line) and repeat units (orange line). Chr1 is the sex chromosome Z. Gene models were predicted by homology-based prediction using GeMoMa input with all genes of *B. mori* (Li *et al*. 2024). Repeat units were classified and quantified using a *de novo* annotation method (Li *et al*. 2024). b) Mapping of eight common genes onto the T2T_W and Z chromosomes. Four genes out of 26 T2T_W annotated protein-coding genes were shared in both chromosomes with >95% identity using full CDS (Supplementary Table 4). Genes shown on the figure by numbers 1–4 are 1: *Bmor18260*, 2: *Bmor18266*, 3: *Bmor18268*, 4: *Bmor18270* (Supplementary Table 4).

**Fig. S6.** GC content on the W chromosome. GC contents (orange curve) were determined in a non-overlapping 1 Mb window. The broken line denotes the total GC content of the W (47.5%). The black bar marks a domain of 8.5–9.5 Mb which shows higher GC content than the average. It also contains vestiges of telomeric repeats (TTAGG)*_n_* and the telomere-specific retrotransposon SART1, suggesting that (i) a fusion between small chromosomes may have occurred at this domain, or (ii) remnants of telomeric repeats and SART1 were transferred to this domain by transposons.

**Supplementary Tables**:

**Table S1.** Sources of sequence data from Dazao/P50T.

|  | PacBio HIFI (>5kb) | NGS | | Hi-C | Utra-long ONT (>50kb) |
| --- | --- | --- | --- | --- | --- |
| Number of moths^a^ | 1 female | 10 males | 1 female | 1 female | 1 female |
| Dazao (Gb) | 39.5 | 65.9 | 70.8 | 61.5 | 79.4 |
| P50T (Gb) | 60.7 | 63.0 | 60.5 | NA | 78.3 |

^a^ Genomic DNA extracted for sequencing

**Table S2.** Comparison of W-RAPD markers found in W-assemblies with PCR mapping.

| O: W-RAPD marker band successfully detected by PCR.  X: W-RAPD marker band could not be detected by PCR.  ^a^ PCR did not distinguish two differing W-marker sites found for W-Rikishi and for W-Bonsai. |  |
| --- | --- |

**Table S3.** W-linked BAC clones mapped on T2T_W.

| W-BAC | Sequenced by PacBio HIFI | BAC-end BES acc # | BAC-end BET acc # | Mapped positions in T2T DazaoW | length (bp) | Accession # |
| --- | --- | --- | --- | --- | --- | --- |
| 031A14 | T2TW:4224453..4392008 | DE292528 | DE339433 | 4224453..4392008 | 167556bp | CNA0144055 |
| 041K20 | T2TW:10108741..10313045 | DE295953 | DE342914 | 10108741..10313045 | 204305bp | CNA0144055 |
| 045J23 | T2TW:5891373..6064142 | DE297313 | DE344295 | 5891373..6064142 | 172770bp | CNA0144055 |
| 046E13 | T2TW:9947846..10118623 | DE297540 | DE344525 | 9947846..10118623 | 170718bp | CNA0144055 |
| 060O19 | T2TW:3420868..3618721 | DE302407 | DE349409 | 3420868..3618721 | 197854bp | CNA0144055 |
| 091N05 | T2TW:5195424..5365829 | DE312046 | DE359574 | 5195424..5365829 | 170406bp | CNA0144055 |
| 504I09 | T2TW:7578732..7741415 | DE379322 | DE401044 | 7578732..7741415 | 162684bp | CNA0144055 |
| 510H14 | T2TW:6748057..6914422 | DE381917 | DE403241 | 6748057..6914422 | 166366bp | CNA0144055 |
| 617I13 | T2TW:305950..3110885 | DE318984 | DE366582 | 3005125..3110885 | 105161bp | CNA0144055 |
| 063P01 | T2TW:6227745..6250180 | DE303326 | DE350302 | 6028843..6250180 | 221338bp | CNA0144055 |
| 065E18 | T2TW:7201510..7362211 | DE303778 | DE350768 | 7201510…7362211 | 160702bp | CNA0144055 |
| 082J01 | T2TW:11117769..11288874 | DE309410 | DE356494 | 11117625..11288874 | 171250bp | CNA0144055 |
| 083K06 | T2TW:7392924..7620753 | DE309683 | DE356850 | 7392924..7620753 | 227829bp | CNA0144055 |
| 089L03 | T2TW:5083633..5307286 | NA | DE358910 | 5083633..5307286 | 223653bp | CNA0144055 |
| 094K19 | T2TW:9937182..10084230 | NA | NA | 9937182..10084230 | 147094bp | CNA0144055 |
| 101J20 | T2TW:3060105..3287930 | NA | NA | 3060105..3287930 | 227876bp | CNA0144055 |
| 131N18 | T2TW:7415534..7620753 | NA | NA | 7415534..7620753 | 205220bp | CNA0144055 |
| 182J02 | T2TW:4402218..4534150 | NA | NA | 4402218..4534150 | 131933bp | CNA0144055 |
| 186I21 | T2TW:6998599..7176955 | NA | NA | 6998599..7176955 | 178357bp | CNA0144055 |
| 204B24 | T2TW:6608193..6760597 | NA | NA | 6608193..6760597 | 152405bp | CNA0144055 |
| 235G05 | T2TW:5680495..5872616 | NA | NA | 5680495..5872616 | 192122bp | CNA0144055 |
| 255D14 | T2TW:9777804..9994613 | NA | NA | 9777804..9994613 | 216810bp | CNA0144055 |
| 278H04 | T2TW:5339452..5488107 | NA | NA | 5339452..5488107 | 148656bp | CNA0144055 |
| 309N16-tig1 | T2TW:4090935..4168314 | NA | NA | 4090935..4168314 | 77380bp | CNA0144055 |
| 309N16-tig2 | T2TW:4361727..4422836 | NA | NA | 4361727..4422836 | 61110bp | CNA0144055 |
| 313C01 | T2TW:5762139..5935508 | NA | NA | 5762139..5935508 | 175370bp | CNA0144055 |
| 332A20 | T2TW:10076905..10260163 | NA | NA | 10076905..10260163 | 183259bp | CNA0144055 |
| 341N05 | T2TW:7157335..7337507 | NA | NA | 7157335..7337507 | 182173bp | CNA0144055 |
| 345I20 |  | NA | NA | 2995591..3121884 | 116071bp | AB901471.1 |
| 353P15 | T2TW:10010424..10020545 | NA | NA | 10010424..1020545 | 10122bp | CNA0144055 |
| 501K19 | T2TW:2137016..2187641 | DE378805 | DE400010* | 2095899..2187641 | 91743bp | CNA0144055 |
| 503H13 | T2TW:6492340..6590457 | DE379433 | DE400559 | 6492340..6590457 | 98117bp | CNA0144055 |
| 506N13 | T2TW:5935503..6026081 | DE380633 | DE401909 | 5845199..6053699 | 207901bp | CNA0144055 |
| 515I04 | T2TW:7537450..7739462 | DE383750 | DE405068 | 7537450..7739462 | 202013bp | CNA0144055 |
| 523B16 | T2TW:4097884..5123119 | DE386478 | DE407832 | 4097884…4140847 | 42963bp | CNA0144055 |
| 526A03 | T2TW:6532455..6558417 | DE387389 | DE408826 | 6531155..6557747 | 26593bp | CNA0144055 |
| 528F18 | T2TW:5339934..5420210 | DE388253 | DE409617 | 5304504..5438741 | 134238bp | CNA0144055 |
| 534I17 | T2TW:7321328..7423794 | DE390383 | DE411744 | 7311885..7459840 | 147956bp | CNA0144055 |
| 539P05 | T2TW:5243229..5269774 | DE392287 | DE413567 | 5108217..5277024 | 168808bp | CNA0144055 |
| 540C16 | T2TW:7321328..7441880 | DE392360 | DE413627 | 7304917..7466036 | 161120bp | CNA0144055 |
| 551B14 | T2TW:8506670..8574356 | DE396198 | DE417458 | 8458201..8574356 | 116156bp | CNA0144055 |
| 626P18 | T2TW:9014680..9097204 | NA | DE369509 | 9014680..9145271 | 130592bp | CNA0144055 |
| 633H12 | T2TW:8182724..8248852 | DE323367 | DE371574 | 8169705..8277842 | 108138bp | CNA0144055 |
| 636C03 | T2TW:3054605..3063377 | DE324127 | DE372354 | 3017016..3149029 | 132014bp | CNA0144055 |
| 645E04 | T2TW:6671389..6769415 | DE326470** | DE375174 | 6671389..6776080 | 104692bp | CNA0144055 |
| 653C19 | T2TW:6945689..7148025 | DE328890 | DE377729 | 6945689..7148025 | 202337bp | CNA0144055 |
| 002I18 |  | DE284138 | DE330190 | 5896569..6072824 | 176256bp |  |
| 020D20 |  | DE289461 | DE335973 | 458602..559794 | 101193bp |  |
| 025F11 |  | DE290996 | DE337652 | 5761214..5934202 | 172989bp |  |
| 025J12 |  | DE291088 | DE337728 | 7170825..7346872 | 176047bp |  |
| 025N02 |  | DE291170 | DE337800 | 9127431..9231830 | 104400bp |  |
| 027E07 |  | DE291573 | DE338288 | 4949996..5123122 | 173131BP |  |
| 028N09 |  | DE291960 | DE338746 | 6392672..6589159 | 196488bp |  |
| 034A11 |  | DE292495 | DE340454 | 6901425..7078285 | 176861bp |  |
| 038H07 |  | DE294902 | DE341902 | 4393539..4594176 | 200638bp |  |
| 047K10 |  | DE298013 | DE344987 | 10175348..10260098 | 84751bp |  |
| 063P01 |  | DE303326 | DE350302 | 6028843..6248642 | 219800bp |  |
| 083K06 |  | DE309683 | DE356850 | 7391654..7654875 | 263222bp |  |
| 088H16 |  | DE311046 | DE368500 | 6869145..7065261 | 196117bp |  |
| 512L05 |  | DE382738 | DE404054 | 5408111..5523893 | 115783bp |  |
| 528F18 |  | DE388253 | DE409617 | 5304504..5438741 | 134238bp |  |
| 540F15 |  | DE392427 | DE413694 | 2927359..3043408 | 116049bp |  |
| 542J24 |  | DE343210 | DE414513 | 6765530..6913130 | 147601bp |  |
| 557P23 |  | DE398670 | DE419881 | 10400209..10472702 | 72494bp |  |
| 066F22 |  | DE318984 | DE366190 | 2401392..2578907 | 177516bp |  |
| 621C11 |  | DE319998 | DE367738 | 6211354..6342811 | 131458bp |  |
| 631H13 |  | DE322709 | DE370894 | 6995293..7143359 | 148067bp |  |
| 654C22 |  | DE329176 | DE378014 | 2089356..2176460 | 89105bp |  |
| 039L08 |  | DE295294 | DE342315 | 6893007..7081307 | 188301bp | AB900928.1 |
| 501F13 (1C7C) | | DE378686 | DE399894 | 9167617..9297112 | 129496bp |  |
| 513M03 (5A2G) | | DE383132 | DE404446 | 6578873..6724239 | 145367bp |  |
| 514F08 (5H4C) | | DE383334 | DE404651 | 8239324..8458674 | 219351bp |  |
| 529G07 (10E4D) | | DE388586 | DE409958 | 2212323..2388072 | 175750bp |  |
| 544H03 |  | DE393854 | DE415124 | 946561..1114051 | 167491bp |  |
| 557L15 (19K8F) | | DE398568 | DE419806 | 2764690..2870113 | 105424bp |  |
| S-1/6_AB055391_lambda | |  |  | 1887324..1701997 | 14683bp | AB055391.1 |
| *BET may be chimeric. | | | | | | |
| **No consistent hit in T2T_W | | | | | | |

**Table S4.** Annotation of protein-coding genes on T2T_DazaoW.

**Table S5.** gff files of W-linked and chr25-linked zinc finger motif genes.

| Gene name | Chr | mRNA/Exon | CDS/Ex location in chr25 | Length (nt) | aa length | Dir | Ex # | Accession # |
| --- | --- | --- | --- | --- | --- | --- | --- | --- |
| *z21* | 25 | mRNA | 5617131..5632217 | 1800 | 599 | F | 11 | XM_012688988 |
|  |  | Ex1 | 5617131..5617155 | 25 |  |  |  |  |
|  |  | Ex2 | 5619957..5620135 | 179 |  |  |  |  |
|  |  | Ex3 | 5620207..5620494 | 288 |  |  |  |  |
|  |  | Ex4 | 5621801..5621957 | 157 |  |  |  |  |
|  |  | Ex5 | 5622363..5622472 | 110 |  |  |  |  |
|  |  | Ex6 | 5623952..5624142 | 191 |  |  |  |  |
|  |  | Ex7 | 5626112..5626248 | 137 |  |  |  |  |
|  |  | Ex8 | 5626802..5627010 | 209 |  |  |  |  |
|  |  | Ex9 | 5630512..5630682 | 171 |  |  |  |  |
|  |  | Ex10 | 5631327..5631455 | 129 |  |  |  |  |
|  |  | Ex11 | 5632014..5632217 | 204 |  |  |  |  |
| *z22* | 25 | mRNA | 5581687..5594957 | 1800 | 599 | F | 10 | KM_004924492 |
|  |  | Ex1 | 5581687..5581890 | 204 |  |  |  |  |
|  |  | Ex2 | 5581970..5582257 | 288 |  |  |  |  |
|  |  | Ex3 | 5583318..5583474 | 157 |  |  |  |  |
|  |  | Ex4 | 5583860..5583969 | 110 |  |  |  |  |
|  |  | Ex5 | 5584560..5584750 | 191 |  |  |  |  |
|  |  | Ex6 | 5589336..5589472 | 137 |  |  |  |  |
|  |  | Ex7 | 5590129..5590337 | 209 |  |  |  |  |
|  |  | Ex8 | 5590672..5590842 | 171 |  |  |  |  |
|  |  | Ex9 | 5593585..5593713 | 129 |  |  |  |  |
|  |  | Ex10 | 5594754..5594957 | 204 |  |  |  |  |
| *z2* | 25 | mRNA | 5643871..5636599 | 1173 | 389 | R | 9 | XM_004924492 |
|  |  | Ex1 | 5643871..5643328 | 544 |  |  |  |  |
|  |  | Ex2 | 5642228..5642177 | 52 |  |  |  |  |
|  |  | Ex3 | 5641832..5641744 | 89 |  |  |  |  |
|  |  | Ex4 | 5641642..5641591 | 52 |  |  |  |  |
|  |  | Ex5 | 5641132..5641023 | 110 |  |  |  |  |
|  |  | Ex6 | 5639699..5639615 | 85 |  |  |  |  |
|  |  | Ex7 | 5638591..5638462 | 130 |  |  |  |  |
|  |  | Ex9 | 5636865..5636830 | 36 |  |  |  |  |
|  |  | Ex10 | 5636623..5636599 | 75 |  |  |  |  |
| *z1L* | 25 | mRNA | 5643871..5636599 | 1083 | 360 | R | 8 | Alternative splicing isoform of z2 |
|  |  | Ex1 | 5643871..5643328 | 544 |  |  |  |  |
|  |  | Ex2 | 5642228..5642177 | 52 |  |  |  |  |
|  |  | Ex3 | 5641832..5641744 | 89 |  |  |  |  |
|  |  | Ex4 | 5641642..5641591 | 52 |  |  |  |  |
|  |  | Ex5 | 5641132..5641023 | 110 |  |  |  |  |
|  |  | Ex6 | 5639699..5639615 | 85 |  |  |  |  |
|  |  | Ex7 | 5638591..5638462 | 130 |  |  |  |  |
|  |  | Ex8 | 5687708..5637688 | 21 |  |  |  |  |
| *z3* | 25 | mRNA | 5652396..5671969 | 1371 | 456 | F | 11 | XM_004924490 |
|  |  | Ex1 | 5652396..5652414 | 19 |  |  |  |  |
|  |  | Ex2 | 5654247..5654867 | 621 |  |  |  |  |
|  |  | Ex3 | 5657438..5657492 | 55 |  |  |  |  |
|  |  | Ex4 | 5657920..5658029 | 110 |  |  |  |  |
|  |  | Ex5 | 5659260..5659308 | 49 |  |  |  |  |
|  |  | Ex6 | 5660313..5660401 | 89 |  |  |  |  |
|  |  | Ex7 | 5667145..5667225 | 81 |  |  |  |  |
|  |  | Ex8 | 5669248..5669332 | 85 |  |  |  |  |
|  |  | Ex9 | 5669746..5669875 | 130 |  |  |  |  |
|  |  | Ex10 | 5670369..5670404 | 36 |  |  |  |  |
|  |  | Ex11 | 5671874..5671969 | 96 |  |  |  |  |
| *z20-1A* | T2T_DazaoW | mRNA | 6977900..6969261 | 915 | 304 | R | 6 | AB901470.1 |
|  |  | Ex1 | 6977900..6977898 | 3 |  |  |  |  |
|  |  | Ex2 | 6977531..6977341 | 191 |  |  |  |  |
|  |  | Ex3 | 6975158..6975022 | 137 |  |  |  |  |
|  |  | Ex4 | 6974461..6974253 | 209 |  |  |  |  |
|  |  | Ex5 | 6972168..6971997 | 171 |  |  |  |  |
|  |  | Ex6 | 6969404..6969261 | 204 |  |  |  |  |
| *z20-1B* | T2T_DazaoW | mRNA | 7003537..7015878 | 915 |  |  |  |  |
|  |  | Ex1 | 7003537..7003539 | 3 | 304 | F | 6 | AB919126.1 |
|  |  | Ex2 | 7003906..7004096 | 191 |  |  |  |  |
|  |  | Ex3 | 7006279..7006415 | 137 |  |  |  |  |
|  |  | Ex4 | 7006976..7007184 | 209 |  |  |  |  |
|  |  | Ex5 | 7012971..7013141 | 171 |  |  |  |  |
|  |  | Ex6 | 7015675..7015878 | 204 |  |  |  |  |
| *z20-2* | T2T_DazaoW | mRNA | 5134384…5140286 | 894 | 297 | F | 6 | AB919127.1 |
|  |  | Ex1 | 5134384..5134386 | 3 |  |  |  |  |
|  |  | Ex2 | 5134818..5135008 | 191 |  |  |  |  |
|  |  | Ex3 | 5135932..5136068 | 137 |  |  |  |  |
|  |  | Ex4 | 5136293..5136480 | 188 |  |  |  |  |
|  |  | Ex5 | 56138565..5138735 | 171 |  |  |  |  |
|  |  | Ex6 | 5140083..5140286 | 204 |  |  |  |  |
| *z20-3* (pseudogene) | T2T_DazaoW | mRNA | 3042946..3047663 |  |  | F |  | AB901471.1 |
|  |  | Ex1 | missing |  |  |  |  |  |
|  |  | Ex2 | missing |  |  |  |  |  |
|  |  | Ex3 | 3042946..3043082 | 137 |  |  |  |  |
|  |  | Ex4 | 3043677..3043859 | 183 |  |  |  |  |
|  |  | Ex5 | 3045945..3046115 | 171 |  |  |  |  |
|  |  | Ex6 | 3047460..3047663 | 204 |  |  |  |  |
| *z20-S* in yCs | T2T_yCsW | mRNA | 3806370..3806218 | 153 | 50 | R |  |  |
|  |  | Ex1-Ex5 | missing |  |  |  |  |  |
|  |  | Ex6 | 3806370..3806218 | 153 |  |  |  |  |
| *z1-1A* | T2T_DazaoW | mRNA | 6946455..6951524 | 1083 | 360 | F | 8 | AB900928.1 |
|  |  | Ex1 | 6946455..6946998 | 544 |  |  |  |  |
|  |  | Ex2 | 6947697..6947748 | 52 |  |  |  |  |
|  |  | Ex3 | 6948123..6948215 | 89 |  |  |  |  |
|  |  | Ex4 | 6938306..6948357 | 52 |  |  |  |  |
|  |  | Ex5 | 6948813..6948922 | 110 |  |  |  |  |
|  |  | Ex6 | 6949847..6949931 | 85 |  |  |  |  |
|  |  | Ex7 | 6950608..6950737 | 130 |  |  |  |  |
|  |  | Ex8 | 6951504..6951524 | 21 |  |  |  |  |
| *z2L-1A* | T2T_DazaoW | mRNA | 6946455..6952598 | 1161 | 386 | F | 9 | AB900928.1 |
|  |  | Ex1 | 6946455..6946998 | 544 |  |  |  |  |
|  |  | Ex2 | 6947697..6947748 | 52 |  |  |  |  |
|  |  | Ex3 | 6948123..6948215 | 89 |  |  |  |  |
|  |  | Ex4 | 6938306..6948357 | 52 |  |  |  |  |
|  |  | Ex5 | 6948813..6948922 | 110 |  |  |  |  |
|  |  | Ex6 | 6949847..6949931 | 85 |  |  |  |  |
|  |  | Ex7 | 6950608..6950737 | 130 |  |  |  |  |
|  |  | Ex9 | 6952349..6952383 | 35 |  |  |  |  |
|  |  | Ex10 | 6952535..6952598 | 64 |  |  |  |  |
| *z1-1B* | T2T_DazaoW | mRNA | 7046150..7041081 | 1083 | 360 | R | 8 | AB919124.1 |
|  |  | Ex1 | 7046150..7045607 | 544 |  |  |  |  |
|  |  | Ex2 | 7044908..7044857 | 52 |  |  |  |  |
|  |  | Ex3 | 7044482..7044394 | 89 |  |  |  |  |
|  |  | Ex4 | 7044299..7044248 | 52 |  |  |  |  |
|  |  | Ex5 | 7043792..7043683 | 110 |  |  |  |  |
|  |  | Ex6 | 7042758..7042674 | 85 |  |  |  |  |
|  |  | Ex7 | 7041997..7041868 | 130 |  |  |  |  |
|  |  | Ex8 | 7041101..7041081 | 21 |  |  |  |  |
| *z2L-1B* | T2T_DazaoW | mRNA | 7046150..7040007 | 1161 | 386 | R | 9 | AB919124.1 |
|  |  | Ex1 | 7046150..7045607 | 544 |  |  |  |  |
|  |  | Ex2 | 7044908..7044857 | 52 |  |  |  |  |
|  |  | Ex3 | 7044482..7044394 | 89 |  |  |  |  |
|  |  | Ex4 | 7044299..7044248 | 52 |  |  |  |  |
|  |  | Ex5 | 7043792..7043683 | 110 |  |  |  |  |
|  |  | Ex6 | 7042758..7042674 | 85 |  |  |  |  |
|  |  | Ex7 | 7041997..7041868 | 130 |  |  |  |  |
|  |  | Ex9 | 7040256..7040222 | 35 |  |  |  |  |
|  |  | Ex10 | 7040070..7040007 | 64 |  |  |  |  |
| *z1-2* | T2T_DazaoW | mRNA | 5164025..5151891 | 1083 |  | R | 8 | AB919125.1 |
|  |  | Ex1 | 5164025..5163482 | 544 |  |  |  |  |
|  |  | Ex2 | 5162784..5162733 | 52 |  |  |  |  |
|  |  | Ex3 | 5162358..5162270 | 89 |  |  |  |  |
|  |  | Ex4 | 5162175..5162124 | 52 |  |  |  |  |
|  |  | Ex5 | 5161667..5161558 | 110 |  |  |  |  |
|  |  | Ex6 | 5153585..5153501 | 85 |  |  |  |  |
|  |  | Ex7 | 5152839..5152710 | 130 |  |  |  |  |
|  |  | Ex8 | 5151911..5151891 | 21 |  |  |  |  |
| *z1-3* | T2T_DazaoW | mRNA | 3057304..3052247 | 1083 |  | R | 8 | AB901471.1 |
|  |  | Ex1 | 3057304..3056761 | 544 |  |  |  |  |
|  |  | Ex2 | 3056063..3056012 | 52 |  |  |  |  |
|  |  | Ex3 | 3055637..3055549 | 89 |  |  |  |  |
|  |  | Ex4 | 3055454..3055403 | 52 |  |  |  |  |
|  |  | Ex5 | 3054946..3054837 | 110 |  |  |  |  |
|  |  | Ex6 | 3053916..3053832 | 85 |  |  |  |  |
|  |  | Ex7 | 3053164..3053035 | 130 |  |  |  |  |
|  |  | Ex8 | 3052267..3052247 | 21 |  |  |  |  |
| *Bmor18276* | T2T_DazaoW | mRNA | 7129979..7131698 | 798 | 265 | F | 4 |  |
|  |  | Ex1 | 7129979..7130134 | 156 |  |  |  |  |
|  |  | Ex2 | 7130336..7130507 | 192 |  |  |  |  |
|  |  | Ex3 | 7131054..7131185 | 132 |  |  |  |  |
|  |  | Ex4 | 7131361..7131698 | 338 |  |  |  |  |

**Table S6.** Primer list.

| **Primer name** | **Sequence(5’->3’)** |  |
| --- | --- | --- |
| *z20*-5’RACE primer | GCTCGATCACTTTCTCTAAAACAGAGATGT |  |
| *z20*-3’RACE primer | CATCTCTGTTTTAGAGAAAGTG | |
| *z20*-S-5’RACE primer | GAAGACATGCGTCTTATGTTGTACCCCTCG | |
| *z1* FISH_a | CATAACATTTCATATTTCATTCAACACATTC | |
| *z1* FISH_b | ACGCTGGGTGAGAATCACCTCAC | |

**Supplementary Discussion 1: Contamination of *B. mori* p50ma strain**

The National Bio-Resource Project “Silkworm” (NBRP Silkworm) News Letter, February 27, 2024, <https://nbrp.jp/public/news/news/news-240227/> reported “Genetic contamination has been confirmed in the p50 strain managed by NBRP Silkworm. Specially, a survey in January 2024 confirmed the contamination of the W chromosome from the Sakado Japanese *B. mandarina* strain in the p50 strain.” In the paper of Lee *et al.* (2024), this contaminated *B. mori* p50 strain termed p50ma strain was used for W sequencing, confirming that the W-Lee sequence is that of Japanese *B. mandarina*.

**Supplementary Discussion** **2: Variability of *Fem* clusters**

**Structure of the *Fem* region**

The main cluster of the *Fem* gene of *B. mori* Dazao strain consists of 68 units, each composed of approximately 750 bp including the 29 nt *Fem* piRNA sequence (Fig. 4a). The majority of each 29 nt *Fem* piRNA sequence consists of the canonical sequence (5’-TACCTCTTTTTGTCAATTCATAAAGTCAT-3’), but occasional noncanonical sequences are present in various locations. For example, a noncanonical sequence (5’-TACCTCTTTTTGTCAATTCATAAA**A**TCAT-3’) appeared in units 22, 24, and 26. Similarly, another noncanonical sequence (5’TACCTCTTT**~~TT~~**GTCAATTCATAAAGTCAT-3’) appeared in units 45 and 52. It is unlikely that the same mutations occurred coincidentally in different units, like units 22, 24 and 26, or 45 and 52. Phylogenetic analysis (Fig. 4b) showed that from units 21 to 26 the canonical-noncanonical pattern of pair 21-22 aligned three times, indicating pair 21-22 amplified to form unit alignment 21-26. Similarly, it is conceivable that the sequence from units 39–45 acted as a template which amplified, resulting in the sequence from units 46-52. Alternatively, it is also possible that the sequence for units 46-52 acted as a template and amplified, resulting in the sequence for units 39-45. In the region with repetitive sequences spanning 750 bp, there are also sequences thought to be the result of amplification at other sites like telomere repeats and rDNA repeats (Kobayashi 2011).

Moreover, two copies of the non-LTR retrotransposon BMC1 were inserted between units 26-27 in the *Fem* cluster of the Dazao strain. One of these BMC1 retrotransposons was full-length with target site duplication sequences (GTAGTAGATA) at both ends and a poly(A) tail at the 3’ end. However, the other BMC1 was truncated by approximately 1600 bp at the 3’end. Interestingly, the sequence corresponding to the target site duplication on the 5’ end was identical to that of the other full-length BMC1 (GTAGTAGATA).

We also compared the composition of the *Fem* clusters of the other two W mutant strains used in this study, Sex-limited Zebra (Zebra) and Sex-limited Yellow cocoon (yCs), with those of the Dazao strain (Fig. 4c). First, there were differences in the number of units of the main *Fem* cluster in each strain. The Dazao strain had 68 units, the Zebra strain had 67 units, and the yCs strain had 78 units. Further, the other TE insertions in the Dazao W *Fem* cluster were the same as in the Zebra W and yCs W strains. Second, two BMC1s, a full-length and a 3’ truncated-type, were inserted between *Fem* cluster units 26 and 27 in Dazao W, whereas only one full-length BMC1 was inserted between units 28 and 29 in Zebra W. In contrast, the yCs W had only one full-length BMC1 inserted between units 20 and 21 of the *Fem* cluster. Moreover, the target site duplication sequence of the full-length BMC1 was identical among the three strains. This clearly indicated that the 3’ truncated type BMC1 in Dazao strain units 26 and 27 was amplified using the adjacent full-length BMC1 as a template. In the yCs strain, it appeared that an approximately 6 kbp sequence corresponding to units 1-8 of the *Fem* cluster gene of the DazaoW underwent deletion. Thus, in the cluster region of *Fem* on the *B. mori* W chromosome, it appears that repeated cycles of amplification and deletion from short to long sizes occurred, leading to the current state.

**Characteristics of small *Fem* clusters outside of the large cluster**

Small clusters of the 750 bp *Fem* unit were found in three locations on the W chromosome, separate from the main cluster (Fig. 4a). The first contained four units of the *Fem* sequences (designated as F4-copy) at ChrW:2779183..2781523. The second contained three units (F3-copy) at ChrW:4098615..4100167, and the remaining one contained two units (F2-copy) at ChrW:4920350..4921140. Since these three small cluster genes have the same TSS and 5’ domain sequences as those of the main *Fem* cluster gene, we propose that these three small clusters were formed by amplification of the 5’ segment of the main *Fem* cluster. Sequence analysis clearly showed the history of the process as follows (see Fig. 4d): Initially, the sequence containing the 1211 bp TSS domain and the unique 450 bp 5’ portion of the main *Fem* cluster served as a template and was amplified at the position of the F4-copy on the W. Subsequently, a 3’ portion of BMC1 was inserted into the first unit of the F4-copy. Additionally, a full BMC1 was inserted between the TSS domain and a 450 bp 5’ unique sequence (Fig. 4d). Then, this F4-copy served as a template and was amplified at the positions of the F3- and F2-copies. Subsequently, intra-chromosomal recombination occurred between units 1 and 4 of the F2-copy, resulting in the deletion of the sequence containing *Fem* units 2 and 3 in the original F2-copy. Subsequently, an unknown sequence was inserted after the TSS domain. Additional complex mutations occurred to generate the F3-copy. First, intra-chromosomal recombination occurred between the BMC1 inserted at the unique 450 bp 5’ sequence and a BMC1 inserted into the first *Fem* unit, resulting in deletion of the 450 bp sequence. Then, intra-chromosomal recombination occurred between *Fem* units 1 and 3, resulting in deletion of *Fem* units 2 and 3. Finally, *Fem* unit 4 was duplicated.

**Supplementary Discussion 3: *z1:z20* pair is a long retrotransposon**

**Structure of *z1-1:z20-1* gene pairs**

Detailed analysis of the region of approximately 130 kb containing *z1-1A: z20-1A* and *z1-1B: z20-1B* of the W chromosome showed this region to be a so-called giant palindrome centered between *z20-1A* and *z20-1B*, exhibiting a mirror image as shown in Figure 5c. We confirmed this mirror-image structure by independent sequencing of the W chromosome of the p50 strain. Furthermore, sequencing and assembly of the W of the sex-limited Zebra strain also showed a similar giant mirror image (Fig. 2c). From these findings, we became convinced of the accuracy of the sequencing and assembly of this region.

This mirror image region is thought to have been formed by amplification as observed in other regions on the W chromosome (W-Rikishi RAPD and *Fem* cluster). Slight differences in the insertion status of transposable elements were observed on the right and left sides of this structure. The history of the formation of the *z1-1A: z20-1A* and *z1-1B: z20-1B* regions can be traced by analyzing these differences in detail. *ChoBm1* is present at both the left and right ends of this configuration. Whereas the *ChoBm1* on the right is of the complete type, the one on the left is of a truncated type. Therefore, a long amplification is suggested to have occurred in the region from the right *ChoBm1* to near the center of the figure and acted as a template, leading to extensive amplification, whereas the left *ChoBm1* likely remained incomplete.

**Ancient *z1:z20* structure originated from a translocated copy of the autosomal homologous pair on the W**

Phylogenetic analysis of the *z1:z20* gene pair (Fig. 5a; Supplementary Fig. 4) showed that its copy was originally translocated from the *z2:z21* gene pair on chromosome 25. Then, two gene duplication events occurred on the W, resulting in four gene pairs on the W named *z1-1A:z20-1A*, *z1-1B:z20-1B*, *z1-2:z20-2* and *z1-3: z20-3*. Among them, detailed analysis of the *z1-2:z20-2* sequence revealed a deletion between exon 2 and exon 3 of *z20-2*, suggesting that the transposon *‘Nukegara’* was originally present in *z20-1B* and *z20-1A* and then deleted in *z20-2* (Fig. 5c, d). As shown in Figure 5c and d, the insertion pattern of the TEs in *z1-2:z20-2* differed from that of *z1-1B:z20-1B* and *z1-1A:z20-1A*. However, the insertion sites of *Kimidori, Mipo, Hikikomori*, and seven *Bm1* were conserved, indicating that these elements were present in the ancient *z1:z20* before it split into *z1-1:z20-1* and *z1-2:z20-2* (Fig. 5b). Furthermore, by removing the sequences of uncommon transposable elements from the DNA sequence data of *z1-1B:z20-1B* and *z1-1A:z20-1A* and comparing the results with the current *z2:z21* on chromosome 25 (*z2*:LOC101738733, *z21*:LOC101739116), it is possible to predict the state of the ancient *z1:z20* prototype, a copy of which was translocated from chromosome 25, as illustrated in Figure 5a and b. The most distinctive feature of this ancient *z1:z20* sequence is that it contains seven *Bm1s*. The presence of *Bm1* strongly suggests that this *z1:z20* is one of the long *SBTEs* (Secondary *Bm1* Transposable Elements) (Abe *et al*. 2010) which are retrotransposable elements containing *Bm1*, considered to be a *SINE* of *B. mori*. Upon discovery they were completely misunderstood due to the lack of precedents in genomes of other organisms. It is now known that various types of *SBTEs* exist, and *SBTE* sequences containing *Bm1* elements are transcribed and scattered throughout the genome, similar to retrotransposable elements. This evidence strongly suggests that the translocation of this *z1:z20* pair to the W chromosome was likely the result of its movement as an *SBTE*. Hence, it is thought that *z2:z21* was transcribed into RNA from chromosome 25 in an ancient female genome and the resulting RNA was inserted into the ancient W chromosome as *z1:z20* through reverse transcription. It is assumed that after translocating to the W chromosome, this *z1:z20* was at risk of being deleted due to constant interaction with transposable elements and intrachromosomal recombination. Nevertheless, it is thought that it has persisted and remained functional to this day because it was able to increase its copy number through amplification.

**Supplementary Discussion 4: Comparison of two W-Rikishi RAPD marker sites**

The completed W chromosome sequence data revealed the presence of the W-Rikishi RAPD marker at two distinct positions (Fig. 3). Initially, we considered this to be an assembly error in the sequencing data. This assumption was based on the fact that the W-Rikishi RAPD marker is a complex nested sequence composed of three types of transposable elements. It seemed highly improbable that identical insertions of these transposable elements could have occurred independently at different positions. However, upon conducting a detailed analysis of the flanking sequences around these two positions (designated as W-Rikishi RAPD-1 and W-Rikishi RAPD-2), we found no evidence of assembly errors in our data. Instead, the data proved to be highly accurate, confirming that the W-Rikishi marker is indeed present at two distinct positions.

The detailed analysis of the sequences in each region is shown in Figure 3. By comparing the nested structure of the transposable elements at these two regions we can trace the evolutionary history of each region. Initially, a large region containing W-Rikishi RAPD-1 was amplified at a distant location, which became the W-Rikishi RAPD-2 region. It is likely that the sequence of W-Rikishi RAPD-2 region was identical to W-Rikishi RAPD -1 immediately after amplification, but two regions have since undergone independent changes. In the right side of the W-Rikishi RAPD-1site, a region enclosed by a red line (Fig. 3) was deleted by intrachromosomal recombination between BMC1-like and BMC1 which occurred in the W-Rikishi RAPD-2 site. Additionally, on the left side of the W-Rikishi RAPD-1 site region, there is another area enclosed by a red line. This region has also been deleted in the W-Rikishi RAPD-2 site. Furthermore, within the area enclosed by the red line in the W-Rikishi RAPD-1 site, the 3’ LTR of the LTR-type retrotransposon *Kimono* is present, but the 5’ LTR is missing. However, the missing 5’LTR of the retrotransposon *Kimono* was found within the W-Rikishi RAPD-2 site. Based on these observations, we propose the following sequence of mutations in the left side of the W-Rikishi RAPD-1 region. First, the LTR-type retrotransposon *Kimono* inserted into BMC1. This was followed by the insertion of the non-LTR retrotransposon *Otaku* into *Kimono*, after which another BMC1 inserted into *Otaku*. Subsequently, the non-LTR TREST-W inserted into BMC1, and another BMC1 inserted into TREST-W. Amplification of this region occurred in the W-Rikishi RAPD-2 site while the 5’LTR of *Kimono* was still intact. In contrast, in the W-Rikishi RAPD-1 site, the segment containing the 5’LTR of *Kimono* was deleted, resulting in the current configuration. In the W-Rikishi RAPD-2 site, the area enclosed by the red dotted line in the W-Rikishi RAPD-1 site was deleted, leaving the 5’LTR of the LTR-type retrotransposon *Kimono* intact. In this case, it is also likely that intrachromosomal recombination occurred between BMC1 and BMC1-like. Although it is not possible to reconstruct fully the intermediate stages by analyzing only the remaining sequences in W-Rikishi RAPD-1 and Rikishi RAPD-2 sites on the current W chromosome, the chromosomal changes can still be traced with considerable accuracy. Furthermore, tandem amplifications occurred in the adjacent region on the left side of W-Rikishi RAPD-2, which we designate as amplified region A and amplified region B. Additionally, on the left side of the figure, there is a common nested structure of transposable elements shared between W-Rikishi RAPD-1 and -2. After the W-Rikishi RAPD-2 site was amplified using W-Rikishi RAPD-1 site as a template, additional transposable elements (indicated by red asterisks in Fig. 3) inserted into W-Rikishi RAPD-1 site, leading to the current configuration. Thus, the comparative analysis between the W-Rikishi RAPD-1and W-Rikishi RAPD-2 sites we found illustrates all the characteristic changes observed in the W chromosome in this study, including multiple large amplifications, significant deletions, and the formation of tandem repeats due to the amplification of short sequences.

**References cited in Supplementary discussion**

Abe H, Fujii T, Shimada T, Mita K. 2010. Novel non-autonomous transposable elements on W chromosome of the silkworm, *Bombyx mori.* J Genet. 89:275-287. <https://doi.org/10.1007/s12041-010-0049-3>

Kobayashi T. 2011. Regulation of ribosomal RNA gene copy number and its role in modulating genome integrity and evolutionary adaptability in yeast. Cell Mol Life Sci*.* 68:1395-1403. <https://doi.org/10.1007/s00018-010-0613-2>

Lee J, Fujimoto T, Yamaguchi K, Shigenobu S, Sahara K, Toyoda A, Shimada T, 2024. W chromosome sequences of the two bombycid moths provide an insight into the origin of fem. Mol Ecol. 33:e17434. <https://doi.org/10.1111/mec.17434>

**Supplementary Datasets**:

**Dataset 1.** Multi-fasta CDS sequences of annotated genes on T2T_Dazao W.

>*Bmor18254*

ATGAGTGAGCCTTGTGATGGAGTTGGTGGCGTCAGTTTATCTGACGTCACCTTTTCCAAAAATTCTCCGGAGGTCGGTGCGCCGTCATCAGAAGTGGGATCACCTCCCACTTCTGCTCGCCCTTCTGGCTCGGTTAACAAAAATATGAACACTTCTGTTGATGGGGTGCCGGCTGATTTTTTCACTCAGATGTTGCAAATGATGAAACACGTGTCGGATCGTATGACGTCGCAATCAGATATTTCAAAAGTGAGAATAAATGACGTGTTTTTGCCGTCATACGATCCAGACGCTAATGTTGGGGTTAGAGAATGGTGTCAACATGTGACCATAGCTATGGAAACTTACAACCTGAGCGATTATGAAGTCCGGATGAAGGCTGGTAGTTTGCTTAAGGGTCGCGCAAGGTTGTGGGTTGACAATTGGCTGGTGTCCACCACCACGTGGCAAGAATTGCGTGATGTTTTAATTTCGACTTTTGAACCGGAAAACCGATATTCACGCGATGTTGTGCGTTTTAGAGAACATACTTACGATAATTCTAAAGACATCGCTCAGTTCTTATCACAAGCTTGGGTTTTATGGAGACGTATTACTAAAGATAAGTTGTCGAATGATGACGCGGTTGAGGCTGTTATCGGTTGCATTGGGGATGAGCGCCTGAGAATCGAATTGTTAAATGCGCGAGCTACATCTGTCCCAGAACTTATTTCAGTGGCCTCATCTATAAAAAGTGTTAAACGTCCTCATCCCAATGCATCGATACCGCATCAGAGTGTCGCAAAGCGCCCACGTTTTGATAAGACACCGGGCTTTTGCCAAATCTGTAAAAGGTCGGGTCACGATACGCGTGATTGCCGATACCGCGACAAACCTGAAGGCCCTCAGCCGCGACAACAGGATGGTAAAACTACTCCTCCAGGTGTTGGTAGACCGACATGTACGTTTTGTTCAAGGTTGGGTCATACTTATCAAACGTGTTACAAGCGAGAACGTGCAGTTGTTTCTAATGTCAATTGTGTGGGGACGTCAAAATTAAATGCGATGCCAGTTGTTGTAGGAGGTTTCAAAATTAATGCAATTTTTGATAGTGGAGCCGAATGTTCAGTCATTCGTGAATCTGTAGCTGCAAAGTTGCCGGGAAAACGCGTTGATAGGGTTAGCTATTTGAAAGCTCTCGGTCAATTTACGGTTGTCTCGCTTTCAACATTAACAACAGTTTGCATAATCGACAACTTGCGCGTAGAACTAGAGTTTCACGTCGTTTCCGATATTGAAGTGAGTTCTGATATTTTGATAGGCATTAATTTGATAGAAAATACGAACCTAAGCGTTGTTGTAAATTCACGAGGTGCAATGCTGGTTCATCGACCGATCATTTACCACATGCGATCTCAAAGCCCAAAATTTGGCAAATTAGATTGTGACTTAACCAATGACAATCAGATTAGCGAGTTAAAAATATTGCTGAACAAGTTCGAACACTTGTTTATTCATGGCTACCCGCATACGCGAGTTAATACGGGTGAGCTAGAGATTCGATTAAAGGACGAAAGTAAGACAGTGGAGCGTAGGCCCTATCGGCTTAGTCCAGTTGAACGGGAAAAAGTTCGCGATATCGTTAACGAATTGCTTGAGCATAAAATAATTCGGGAAAGTAAATCTCCTTTTGCAAGCCCAATAATTATAGTTAAAAAGAAAAACGGCAAAGATCGTATGTGTGTAGACTACCGAGAGTTAAATAGGAATACCGTCAGGGACCATTATCCCTTACCGATAATCTCAGACCAAATAGACCAACTGGCCGGGGGTATTTATTTCACCACAATTGACATGGCAGCTGGGTTTCATCAGATACCTATTTCGGAGGGATCAATTGAAAAGACTGCTTTTGTAACTCCCGATGGTTTATATGAATACCTAACTATGCCATTTGGACTTTGCAACGCGGTGTCAGTCTACCAGCGCTGCATTAATAGGGCTCTCGCTCATCTCACGAGCTCACCAGACCAAGTGTGCCAGGTCTATGTCGATGATGTGCTCTCAAAGTGTAAAGATTTTGATCAGGGCTTATCCCATATTGAACGCATATTGATTGCTCTACAAGAAGCTGGCTTTTCTATTAACGTTGATAAAACCGCCTTCTTTAAACGCTCCATCGAGTACTTGGGCAATATAATTGCCGACGGGCAGGTCAGCCCTAGCCCAAGAAAGGTGGAGGCTTTAACGAAGGCACCAATCCCGAAAACTGTCAAACAAGTTAGACAATTCAATGGCCTGGCTGGATATTTCAGACGTTTCATTCCCAACTTCTCGCGAGTTATGGCGCCTTTATATGAACTCACTAAGAACGATACGACATGGGAATGGAACGAAAGGCATGACGAAGCTAGGGATTATATTGTCAAACATCTGTCTACTGCGCCTACCCTTACGCTATTCCAGGAAGACGCACCCATCGAACTCTACACGGACGCCAGCAGCATCGGCTACGGGGCAGTGTTAGTGCAGACATTCGCGGGTCGCCAACATCCAGTCGCGTACATGAGCCAGCGCACCACCGACGCCGAGAGCCGCTACCACTCTTACGAACTTGAAACGTTGGCGGTCGTAAGAGCAATTAAGCATTTTCGGCATTACCTCTACGGCCGAAAATTCAAGGTAATAACCGACTGTAATGCTCTCAAGGCCTCCAAAAACAAAAAGGACTTGCTTCCGCGTATCCATCGGTGGTGGGCGTTTTTGCAAAACTACGAATTCGAGGTGGAGTATAGGAAAGGTGAGCGATTACAGCACGCAGACTTTTTCAGTAGAAACCCTTGCAGCGAGATGTCAGTTAACGTTATGACTCGAGATATGGAATGGCTGCAGATTGAGCAGCACCGCGATGACGTGCTACGCCCAATGATGGAGAGTATAACATCTGGCGATTCCATCGAAGGCTATGTCATGGAAGATGGCATACTTAAGAAAATGCTCACAGATCCTATACTCGGCATACATAAACCAATTGTAGTGCCTAAGTCTTTCCAGTGGAGCTTGATAAACTCCTTCCACGTGACACTGCAGCACCCAGGATGGGAGAAAACTCTGCAGAAGCTACGAGAAACGTACTGGTTTGACAAGATGAACTCGGCTGTTAGACGTTTTGTAGACCATTGTGTTATATGTAGAACCTCGAAAGGACCATCTGGTTCTATACAAGCGCAGCTCCATCCGATCCAGAAACCCACGGCAGCGTTCCAGGTCATTCATATGGACATCACAGGAAAATTGGGCACGAGAAACTCCGAAGGACAATATGAGTACGTAATTGTTGCTATTGACGCATTCACGAAATACATATTACTGAGCTATTCCAATGACAAGAGCCCGAGCAGTACACTCGCGGCACTCAAGCGTGTCATTCACCTGTTTGGCACCCCGGTACAAGTGGTGGTTGATGGCGGTCGGGAGTTTCTCGGCGAGTTCAAAGTCTACACTGACCGCATAGGGATTGACGTGCATTCAACTGCGCCAGGAACAAGCCGAGCAAACGGACAGGTCGAAAGGGTCATGGGCACATTGAAAAATGCCCTTACCATGATCAAAAACTACAGTACTCAAGACTGGCAGACAGCGCTGGAAGCTTTACAGCTCGCTTTCAACTGCACGCCACATCGAGTGACAGGAGTAGCTCCGCTCACTCTCCTGACCCGCCGACAACATTGCATACCGCCAGAGTTACTAAGGTTGGTGAATATTGAGAACGAATGCATAGATTTTGATCTGCTCGAGCAACACGTACGACAGAAAATGATTGATGCGGCGCAATACAACAAGCAACGCTTCGACAGAGGCAAGGCGAAATTGCGCCCATTTCAACGAGGCGACTACGTACTCGTCAAGAACAATACTCGTAACCAGACATCCCTCGACCTCAAATACAGTGAGCCGTATGAAGTATGCAGAATCCTGGACAACGATCGGTACATCATCAAGCGAGTGACCGGCAGAGGGCGCCCTCGTAAAGTCGCCCACGACCAGTTGCGCCGAGCGCCCCAGCCTGGCGAGCAGGACACCGTGTCGACGGACAGTGTAGAGGACCCCGAGCGACAACCGGCGAGCGTGCCGGCCGACGAGACTGAAACAGATGCCACATCTCGGCCCCAGCCCCTGCCGTCTACCTCGCGAGCTGCCCAGGCCTTGGATGCTTAA

>*Bmor18255*

ATGTCTTTCACAAAAAAAATACCACGTCAACCCTTGCAGCCGATCGAGTCGTGGAAGAAACCGGACGTACCCTTTGACACGGTACATAGTGATGTCTTGGGTCCTCTTCCCGAATCGAACGGTTACAAATTTGTTGTCTTGATAATTGACGCTTATAGTAAGTTCTGTCTACTAAATCCTATGCGTAAGCAGGATGCAACAGAGTTAAAATTAGTATTTGAAAACGCTGTATCTTTATTTGGTGCACCTAGGCAGATTGTAACCGATAGGGGTAGAATGTACGAAAATAGATCATTTAGACAATGGGTATCAGACGTAGGTAGTAATTTATTCTTTATAACGCCAGAGATGCACCAGAGTAACGGTCAGGCTGAGAGATATTGCAGAACAGTTCTTAACATGATTCGTGTGGAGGTAAATTTCAGAAACGAAAATTGGTCAGAAGTTTTGTGGAAACTGCAGCTCACCCTGAACGTTACGAAACAAAAGTCCACTCAATATTCACCTTTATATTTGTTAATTGGTAGCGATGCTGTGACGCCCGTCATTCGTTCCGTTGTACGCGATGTGGCTCTAGAAGGAACAAGTGCCAACAGAGAAACCTTACGCGAGCTTGCCCGACAAAGAGCTTCTCAACTGCTGGACAAAAACCGAAAACAGCAGGACACTCGAGTAAATGAACGGCGCAAACCCCCGCGTACTTTCCATGAAAACGACGTGGTCTTCGTGCATAAGAATAGCCAATCTGTTGGAAAGCTGGATTCTGGTATGCGTGGCCCGTACAAAATAGTGCGTGCGCTGCCACATGGACGTTATGAACTGAAGCTTCTTTCTGGCTCACTTGGGAAGACCACGGAAGCTGCTGCCGAATTTATAAGTGCGTGGCACGGAGAGTGGACGCCGGATGTGTGCACCGCATTTTTCGAGTGTGATGAGAATAGCTCTGAGGAAGGTATTGTCGCCGGTCCATCACACCACCATGACGACATAGTACCTCTAGACGAACCTATTAATGCGGGCGAGGACGCTCCGCCGTCAGGAGAGGCCGTGTTAGAAGATGATGATGACATTGGAGGCGTGGCCTAA

>*Bmor18256*

ATGAATGTACTGCAAAGTAGTTTGTCAGACTTGTCGGAGCACTTTTACAAGAAGATGGCTGATTTTGAGGCAAAGCTTCATCCACAGTCAGTATCATCTCCCTCAACATCTTCTCCGGACCTGGCTGCTGAGTTTGCAGTGTTTCGAGCATTTATCACTGAGGCTGTGTCGGGTCTACAAAAACAGATTGAGGTTCTGTCTCACAAACTTGATCACATGGAGATGCATGGCCGCAGGAAGATGTTGCTTTTCCACGGGGTGGTTGAACAGAAACAAGAAAATACATTCGAAATTGTGGCCGACATATTGAAAAAACATTTTTCATTAAATACTGTTTCAGTTCACGATATTAAATGGTGTAACAGAATGAGACAGCCTTCTGCCAGGGAAAGGGCACGACCGATCTTGGTGAAGTTCCATGATTTTAGTTTCCGCAATAAGATCTGGTTTAGTAAGTCTCAATTAAAAAGGACTGGGATAACGGTTTCCGAATTTCTTACGAAACAACGGCACGATGTTTTCATGGCAGCACGGGACAAGTTTGGAGTAACGAACTCGTGGACTAAAGAAGGCACAGTTTATGTTCTCGGCTCAGATGGCAAAAGACATCGGGTGTACTGCCGTAGACAACTGATTGAAATTGATAGGCACACCGTGGAGACTGTTCCTGTACAAACTGCCGCAAAACCAAACACGGCGTCTGTTGCACCAAAGCTTAAGCGTGCGGCCGCCCGTAAATGA

>*Bmor18257*

ATGAAGAATTGTAACCTGTGCAAGATCCAATTCAGTGATGGTGTTCAATGCGGTACGTGCAAGAAGCATTTTGACTTCGGATGCGCTAATATTACAGAAGCTGGATATAGAAAACTGGGCCCCGAAAGAAGGGCGGCATGGAAGTGTTCTGCTTGCAGAAACCCTTCGCAAGCGGTTGCTTCACAGGCCAGCCCAGTCTCTCCAGTTCAGGGCTTAGTGACGCTAGAAATGATCCACGCTGAATTATGCGGCGTCAAAACGAAACTAAATAGTTTATCTCTGCTGCATCAGGAAGTAATAAATATACGTGAAGAACTGTCATTGCTGCGTGAGTCCGTCACTCAAAACATAGAAAAGGTATCTGAGTGTGACGCTAGGCTTATTGAGATTGAAAGCAAGCTCCCAGTGATTGAGCGTACTCAGTCGTCTGTTGAATGTCTCGAATCAGCGATGCGGAAAATGCAGGGAAGGATCGATGCTTTGGAGCAGAGAGCCAGGCTTAATAATGTCGAGATCAAGGGCGTTCCTGTAAGGAAGAACGAAAACCTTTTTACAATCGTCGAGAACATCTCCCAGAGGCTGGGCGTGTCCTTTTCCAGGGCAGACATCAACCATGTTACGAGGGTGCCTACATTTGGATCGGCTGACAAACTCATCATACTAAACTTCCACAATAGGTACACAAAGGAAGATTTTGTCGCCGCTGCTCGTGCACTAAAATTTCTTAATGCCCAGGACATTGGTTTTACTGTTTCTAATCGTATTTTCGTGAATGACCACCTGACTCGTGATACGAAACAACTTCTCACAAAAGTGAAAACATGGGCCAAAAACAAGAATTTTAAGTACATTTGGGTTAAGTTCTGTAAGATCCATGTGCGAAGATCTGACTCGTCTCCGGTTATTATAGTACATACAGAGGCAGATTTAAACAAATTGACTTAG

>*Bmor18258*

ATGACAGAAGAGGAGCTTTTGGACAACTTAGGGAAAATGTTGTTCAAACTGATTGATTCAGACACACTCGAGTGTTTCGGTTGGCCCGACGAGCCAGTTGATGTGGTGTTAGAGAAAGTGATCGAGCACTGCGGAGCTAAACCAGCGGATCGCACGATGTGGACGCGCATGCAACGCCTCCGAGAGAACACGAAGCATCTCTTCCTGTACGTGATAGACGACAAGAACATCGCCCGCATGCTGGACACGCACACCATAGACCAGATCGTCAAACATATACTCGCCCAAGTCAGCGACACCGACAAATGA

>*Bmor18259*

ATGAAAAAATACTTAAAATTCAAGTTCGGGGAGGAGAGTCACTCGCCCCAATATCATGGAGTAGCAGAAATCCCAGAAGCAGAAGCGATGAAAAGAAAGAAACATGAGCAGACAGAGAAGGTTCATCCACCATTACCTAACCGCCCACCGCTGCCGCCGCCGGAACCACTCCTTGCAAATTTCAATAAGGAAATCAATGAAGACGAGGAGACATCTTCAATCACCATAAATATCAAGAAAGATCCAACTGAGAATTATGAAAAGCCTAATGACGTCGAAGAGACCATGTGTCGTAATTTTGTGCGTAACACTTGCAATCGTGGTGCGTCATGTAGATATCTCCACAAAATAATACATTCCCAACTTAAAGAAGTTTACAGATTCTGTATTGATTTTGAAAATAAAAAGTGCACACGTGCAGAATGTTCATATGCTCACGCCACAGTGCACGAGAAAGAACATTTCTTCAGAACGGGCTATTTACCACCACATACATTGAGCCATATTAAGAAAAAAACAGTCCAACCTCCAGCTAAAACCAAAGAAACATCATCCGAGCTGTCGGCGAATTATACCGGCACTACATACCCTCATAGCACCACTTCTGTTCCGATGTACACAAATGCTTCGAATATAAGCATGATGCAAAATCCGTACGAGAAGCAATGGCCGGAAATGGAAGACAAATCGTCGCCCGAGATGGCATATTATAGTGGGCATCCATCTTATCCTCACAGTACGAGCACAGGTTACCCAAACGCTCCGCCCAATACGCCTATAGTGCAAAATCCATACGCCACTGTGATGTCTCCTACGAAGAGGCAGTGGACAGAAATGGAAGAACCCGTGAGTCAACCCACGCACGAGTACGCGGAATACGCTGAAACGGCTGGATCCAAGAAATGCAGAAACTGTGATGTCAACGAGTTCAGATTCCAGCACAACAAAAACAAAATCATGAAAATGATAAAAGACACAGACGACTTGAATCATCGCGTGGGACAAATAACTAAAAAGAACACTAAATTGAATGAAGTACTTGTGTTGCTCGTCGAAGTTGTGATAAGACGCACGTATCGCTAG

>*Bmor18260*

ATGCGCAGTCAGGTTTGGTTTTCTAAAACAAAACTGAAGGGCACCGGCTTTACTGTTTCGGAGTTTCTGACGAAGGCGCGCCACGGCATTTTCATGGCTGCACGCAGTAGATTCGGCATGAAAAAGGTCTGGACCAAGGATGGCTGCGTCTACATTTTGGGAGAGGATGGCATCAAACATCGAGTCTTCACGCAGCGAGATCTTGATCGTCTGCAAAAGAACACTTCGGAAGCAATCCCAGAGGTTGATGCTGCTCCTAAACGCGCTGTTACAAAGCCGGCTGTAGTAAAGGTTAAACGCGCTGCTGCTAGAAAGTAA

>*Bmor18261*

ATGACTGGGAAAGGAAGGGAGCTAGTAGATGTGTTAAAGAGGAGAAGAATAAATATAGCATGTCTGCAAGAGACTAGGTGGAAAGGAGCGAAAGCTAGAGAAATTGGTGAAGGATATAAGCTGTATTATAGTGGAAGTGATGGAAGAAGGAATGGCGTGGGAGTAGTGTTAGATAAGGATTTGAAAGATTGTGTGGCGCATGTCATAAGAACGAGTGATAGGATAATAACCGTAAAAATAGTTTGTGAAAGTGTTATTTTGAATGTTATAAGTGTGTACGCGCCTCAGTCCGGGTGCAAGGAGGACGTGAAAGAGAAATTTTGGCAGGATTTTGATAGTGTTATGATTCGCATTCCAGAGAGTGAAGAGATTTGTATAGGAGGTGATTTTAATGGACATTTAGGAGCTACAAATGAGGGCTATGAAAGGGTGCATGGAGGGTGGGGGTATGGCAATCGTAATGATGATGGAGACTAG

>*Bmor18262*

ATGCGTAAGCAGGATGCAACAGAGTTAAAATTAGTATTTGAAAACGCTGTATCTTTATTTGGTGCACCTAGGCAGATTGTAACCGATAGGGGTAGAATGTACGAAAATAGATCATTTAGACAATGGGTATCAGACGTAGGTAGTAATTTATTCTTTATAACGCCAGAGATGCACCAGAGTAACGGTCAGGCTGAGAGATATTGCAGAACAGTTCTTAACATGATTCGTGTGGAGGTAAATTTCAGAAACGAAAATTGGTCAGAAGTTTTGTGGAAACTGCAGCTCACCCTGAACGTTACGAAACAAAAGTCCACTCAATATTCACCTTTATATTTGTTAATTGGTAGCGATGCTGTGACGCCCGTCATTCGTTCCGTTGTACGCGATGTGGCTCTAGAAGGAACAAGTGCCAACAGAGAAACCTTACGCGAGCTTGCCCGACAAAGAGCTTCTCAACTGCTGGACAAAAACCGAAAACAGCAGGACACTCGAGTAAATGAACGGCGCAAACCCCCGCGTACTTTCCATGAAAACGACGTGGTCTTCGTGCATAAGAATAGCCAATCTGTTGGAAAGCTGGATTCTGGTATGCGTGGCCCGTACAAAATAGTGCGTGCGCTGCCACATGGACGTTATGAACTGAAGCTTCTTTCTGGCTCACTTGGGAAGACCACGGAAGCTGCTGCCGAATTTATAAGTGCGTGGCACGGAGAGTGGACGCCGGATGTGTGCACCGCATTTTTCGAGTGTGATGAGAATAGCTCTGAGGAAGGTATTGTCGCCGATTCATCACACCACCATGACGACATAGTACCTCTAGACGAACCTATTAATGCGGGCGAGGACGCTCCGCCGTCAGGAGAGGCCGTGTTAGAAGATGATGATGACATTGGAGGCGTGGCCTAA

>*Bmor18263*

ATGGCGGAGAGCAAAAACGACGATAGCAAGAATTTATTTTACACGATGGACGCTGTGCCAAAGTTCACGGGTGATGACATCACGTACTCTTCTGCGAAGTGGACACAAGATCTCGACGATAATGCCGATATATTTGGGTGGAGCCAGCAGCAGAAACTCATTATAGCGCGACGATGTTTAGCTGGAACAGCAGAATTATGGCTAAAAAGCGAAAAAACTTTCAGAAGTTACGAAGAACTAAAAGTGGCTTTGATGAAGGAATTCCCTGAGGTGATAAACAGTAAACAAATGCATGAGACAATGAGCAGACGGAAAAAACAACCCAATGAGTCGTTTTATCAATATATGCTCGTAATGAAAGAACTCGGAAAAAGAGCTAAGTTTCCGGATTACGTCGCGATTCAGTATATAATCGACGGAATATCTGACTATGAATCTAACAAGTTGTTATTTTACGGGGTAACGTCGTATTCTGTGCTCAAGGAGAAATTAGTATTATATGAAAGTTTCAAAGAGAAGTCCAAAAAAAGTGTTGATACCATGAGACGAGGTCAGCGAGACACGGCGGTAATGCCTAGACAGGTTCATCGATGTTACAATTGTGGTGAAAGAGGGCACCTATCTAGTTCCTGCCGCAGAGGTATCAAATGCTTCAAGTGCAACGGGTTCGGGCACAAGGGTACGGAGTGCCGCGCATCACAAGGTATGGAAGCTAAAAAAGCATCAAATAATTATTATGGAGGAGGTGATGAACAAGATCGGCGATCTATGTTTGTTCGACAGCAGGAGGACGACGAGAAGAACGAGAACGAACTATACGAACGTGAGCAATGCGATTTAAAAGAGCAAGTGAGTGATATGCCAAATTTTAGTTTTTATTTTTTAGTTGTCAACAACTAA

>*Bmor18264*

ATGGTGAATACTTGTATTGTAAGTGGTTGTAAGCGAGAATCCTATTCAGGTTGTGGAATATCCTTCCATAGAATTCCAGCTAATGAAGAACGGAGGGCTTTGTGGATTACTTCCATGAATCTTGACAATAACGTGTCAAAATTTGCATGCGTTTGTAGTTTGCATTTTAATCAAGATGACTATTTAACAGAAGCTAAAAAAAAGTTAAAACCACAGGCAGTCCCTAAGGTATTTCCAAGAAGCAAGAAAATTACAGTTTTGAGTATTCAAAATATGGAACTAGAATCTTCTATACAGCCATCAACTTCGAGCGATATCACACAAGCAGGAACTTCTTGCGAACGAGGAGAATCTTCTAATAAATATGATGTTGGAATTTCTGGAGAACTGTCCTATATAAAGGTCAAACAAGAGGAATCAAGTACTCAAACGCCTGAAATAAAAAATACCCCAAGAAAAGCAAAATTACGACGAGATGTTAAGGTTTTGAAACAAAAACTTAAAAAACGCGAAATAAAGATTGTAAATTTAAAGTCGATTTTAGATTTGTTAAAGAAAAAATGCGAGAATTTTACAGAATTAGAAACTGTCTTAAACAATAGCTTTTTAAATATTCATAAACATTTTGTAATTAATACTAAATCAGGTAAAGGAGTAAGATATAATGAAGAGATTAAGTCATTTGCTTTGACAGTTTGTTTTTATTCGAAACGAGCTTACTCCTATTTAAGAAATTTTATGTGTCTTCCACACCCTTCGACACTTCGAAAACTTTTGTCAACTTATAACTGTAATGTTGGATTTATGACTGAGGTATTTAATTTTTTAAAAAGTTTGGCAACCAAAACTCCGAGCTTAAAAAATGTTGCTCTCATTTTTGACTCTATGTCTATTAGGACATAA

>*Bmor18265*

ATGTCAGAAGACTACACACAGGGAGTGGAAAGGTTGAAAGAGGTGCTTAACGTTGCCAGTGAATATGGGCTAGAAATTAATTGGAAAAAATGTGAATTGTTGACTAAGAGGGTAGATTATTTAGGTCATAAAATTGAAAATGGCACTGTGAGTCCAATATCTGATAAAGTCGACGCAGTCATGAACTTTCCTGTACCTAAAACATTGAAACAATTACATTCTTTTATAGGACTTACTTCTTATTTCAGGAAGTACATCAAAGACTATGCGCTTATCGCTACACCATTAACAAACCTACTAAAGAAGAACCAAACATTTCAGTTCACTGAAGTTGAAAGAGGAGCATTTAACATTTTGAAACAAAGACTTTGTGAAAAACCAGTATTAAAGATTTATCATCCAGACTTACTCACGGAGTTACACACGGATGCATCAAAACATGCCTATTCTGCTATTCTCATGCAAAGAAGCCCTGATGATGGAGAGTTACATCCAGTTTACTACTCAAGTTGCAAGACTACTGAAAGTGAGAAAAAGTATTCCAGTTATGAGCTGGAGGCTCTAGCAGTTATTGAAGGTGTGAAGAAGTTTCGAAAATATTTATTGGGCATTCCATTTAAGATTGTCACAGACTGTGCTGCATTTGAGATGACTCTACGCAAGAAAGATTTAGTCACAAGAGTTGCTCGATGGGCTTTACTTCTACAAGAGTATGACTATACAGTAGAGCACAAGTCAGGATCCAGAATGAGGCACGTAGATGCCTTAAGCCGAAACCCACCTTATGTAGGAACGGTCACAAGACAAATGCATGACCAGATAAGAATGGCCCAAGAAAATGATGAAGGACTGAGGGCTATAATCGAGGTACTTAAGAGTAGAACATACTCAGACTATTTCTATGAAAATGGATTATTGTATAAAACGTCACTAATGCTACTAGTTGTTCCCAAGGCAATGCAAAACGAAGTGATAAGAAAAGTACATGGCATTGGTCATTTTGGTAAAAAGAAAATGATGGATTTAATAAATAAAGATTATTATATCAAAGATTTAGCAAAGTGTATTGAAGACTTTATTGTAACTTGCATACCATGCATTTTAGCTTCAAGGAAAGAGGGAAAGCAAGAAGGATTTTTACACCCGATAGAGAAAGAAGACTCACCTCTACATACCATTCATATAGATCACGTGGGACCTATGACGGAAACAAAGAAGCAGTATAATTACCTGTTAACTATTGTTGATGCATTTTCTAAGTTTGTCTGGATTTTCCCTACAAAGAGCACCACAAGCAAGGAGGTATTGGACAAACTAAGGATACACCAGCAACATTTTGGAAATCCATGTAGAATTATCAGTGATCGTGGTACAGCATTTACTAGTCATGAATTTAAACAATACTGTGAGGAAGAAGGTATACAACATGTTAAGATCACCACAGGCATCCCAAGAGGAAATGGTCAAGTTGAAAGGATTCACAGAATTATAATATCTGTTCTCACAAAGTTGTGTATTGAACAACCTGATCTATGGTATCGTCATGTGAGTAGAGTTCAACAATGTCTTAATAGTACATTCCAAAGAAGTGTAAACTGTACACCTTTCGAGCTGATGGTAGGAAAAAGAATGAATATTAAAGAAAATGTAGAGATTCACAACCTACTTATGCAAGAAGAGAGAGATTTATTCATAGAAGGAATAGAGCATTTACGCCAAAAGGCCAAGGAGCAAATTCAACTCATACAGGAGGAAAATAGAAGAAGTTACAACAGGAAGAGAAAAGACAGCACTAAGTACAGCATAGGAGATTTGGTGGCTATAAGGAGGACGCAGTATGGCACAGCCATGAAGCTAAAGCCAAAGTTTTTAGGACCTTATCGTGTCACAGGTGTCAAGGGCAATGACCGCTATGAAGTACAGAAGGTGAGTGCTAATACTGAGGGACCTAATAATACTTTGTCTGCTGCAGATTATATGAAACAGTGGCCTGTACTTACCTGA

>*Bmor18266*

ATGACTTCTTATAATTTTAATGATGACATCCAAAACCAACTCATGCGTTTTTGGCAGCTTGAAGAAGTCTGTCCTCAGTCAAATCATTATTTCCCTGAAGAGAAGTTATGTGAAGAACATTTTGTTAAAAATACAACTCGCCCCGCAGACGGACGGTTTTGCGTAAGAATCCCATTGAAACAAGCGCCTGACGTTTTGGGAAGTTCTTATCAGCGAGCTAAACATTGCTTATTATCTTTGGAGCGTCGACTCAAAGGTGATGATTCATTTAGTAAAATGTACAAGGAATTTATGCGCGAGTATAAAAATCTTGGTCACATGTCAGAGTGCAAACCGAATATTGAGTTGCCTCATTACTTCATCCCACATCATGGTGTCCTTCGTGAATGTAGTACGACCACTAAGTTGCGCGTCGTATTTAATGCGAGCAGCCCTACTTCCTCAAGGGTTTCATTTAATGACATTCAAATGATAGGACCCACGGTGCAAGACGATCTTCTGTCAATTCTTTTAAGTTTTCGGCAGCACAAGTATGTTCTTGTAGCTGACGTGGAGAAAATGTATAGGCAGATCGTCGTGCATGAAGCGGACAGACCGTTACAACAGATTATTTGGCGCGAAAACGAGTCAGATTCACTCAAATCATATGCTTTAAACAGAGTCAGTTATGGGACTGCTAGCGCACCCTATCTCGCTAGTAGATGTTCGAAACAAATAGGTATAGAATGTAAGGACCCCACGATAGCGGACATCATTATCCATGATTTTTATGTCGACGATTTGTTGACTGGAAGTGACTTCATCAGCGAAGCATTGCGCATCCGTGAGCAAGTTTCTGCTGAATTAGCATCAGCTTGCATGCCTCTGAGAAAATGGAAGTCCAATGAACCTCAACTGTTATCACAAGCCAGTCATCAACCATCCCTTGAATTAAAACTCGGAGATGCTGAACCTAATAAGACTCTTGGCCTCGGATGGGATTCAGAGTCAGACCAATTTCATTTTCCTGTCAATCCTGTCATTGTTAAGGGAAAAACAAAGCGTGACATCCTCTCTGTCATAGCTCAAATATTTGACCCACTTGGGATCTTATCACCTTTCATCATTTCGATGAAATTAATTTTACAAAGGTTGTGGCTTCAACGCTTATCTTGGGATGAGGAGTTGCCTTCAGAGATATACCAGGCGTGGATCGAAACTGTCGACAGCCTGCCTCATTTGAGCAGCTTGCGCATACCGCGACGTGTCATTTGTGATTCTCACTCCCAGTTGGAGTTACACATTTTTACTGACGCGTCTGAGCGTGCATATGGTGCTTGTCTTTATGTGCGTAGTATTGACTACGACGATGCCGTCTCAGTTCAGCTACTTACCGCTAAGAGCCGGGTTGCTCCCATAAAGCCAACGACTATACCCCGACTAGAACTCTGCGGCGCGTTGATTGGTGCTCGAATTTATGAGAAATGTGTAACTGCATTGCGTGCTAAAATCAACCGCGTTATATTTTGGTCAGATTCTACCATTGTTTTAGGCTGGCTAAAAATGTTGCCATCTAAATTACAGCCATTCGTGCGTAATCGTATTGCAGAGATTTTAGATAAAACTGGAAGCTGCACTTGGAGACATGTCCCGACTGACGAGAATCCCGCTGATTTGATATCGCGTGGTGTCAGTATTAATTCTCTTCAGTTTCACAAGTTATGGTGGCATGGTCCAGATTATTTAAAACGTAGTGACTCGCATTGGCCCACTAACCCATGTGCCAGTGACAAGTTACCTGAGATTAGAAACCAAGTTTCATTACTAACATTATCCAACGCCCCTCGCGAACTTATAGATTTTAGTCGCTTTTCAAGGTTTTCAAGGCTTTTGCGCACCGTTGCGTACGTGTTGCGATTTATAAACAATTTAAAAAAAGGGTCAAAACGTAATGATAAATATTTAAGTGCTGACGAATTGCAGAATGCTAAGGTTTTGATTATGAGACATTCGCAAATGAAATCGTTTCCTGAGATGATTTATTGA

>*Bmor18267*

ATGGATCTGAAACTTATAAATAGTCTAGAAAGCCTCGAGAAGCTTTTCCAAACACGTATGAACGACTATGACCTGAAGCTGCAGAAGGCATCTACCGGAGCTGTTCCAGCACATGTCGATATTTCAACTTTATCGCGTGAGTTTAATGATTTTAAAGCTTTTGTGTGGCAAACACTTTCTCAGATGAAGACCCAGATTGAGCTTCTCAGCCTCGGGTTGGATAGGCATGAGACCATGATGAGAAGAAAAGTTCTACTTCTACATGGTATTCCTGAAAATAAAAATGAAAAATTGCACAAATCTATTGTTGAAGTTTTCACTAGAAACATGAAACTCCCTGAAATTTGCATAGAACATATCAACAATTGTCATCGGCTCGGCAATTCTCAGGGCAAAGTCAGACCTGTCCTAGTGCGTTTCTGCAGCATGGAGCATCGCAATGTTGTATGGGATAATAAAACCAATCTGAAAGGCTCTGGAATCACTGTCTCTGAATTTCTCACCAAAACGAGACACAAGATCTTCACTGCTGCCCGACAACATTTTAGCGTCGGTAACTGCTGGTCAGTGGAAGGAAAAATTGTCATTCTAACACCTGACAAAACTCGTCATAAAATTGAGTGTAAGTCTGAACTGGACGCCTTAACATCCCGATACCCTGCTGCTGCGGCCATCCATAATTCTCCACGGACCAATGATGACCGCGAGACTGCCAAATCTCAGCCAAAAGTGACCAAATCAAGTCGGCGCCGCGTTTAG

>*Bmor18268*

ATGTCTACTGGTATAGTTCCGTGGAACCGTTGTTTAGAACATAATTATACTCGTGAGATCGTGAGATTAATGACCACTTTACCGGTACCGTCTAATCGCAATACGCTGTCGCACTCTTGTACCGAACGCCACGCGCAGTTATTTTTGCCACCAGCAGCAACGATTTCCGTCGAAGGGGGTGACGCATGTGCACCTGAGTACCCTGGAACAGCGTCATGCAAATCGGCGGGTGTGTGTAGCAGTGCTGGTCAGCAGATGGACGGCGTGACGAACAGGATTATTGGAGCATTGCAGGTGCTATCTAAACTTTGA

>Bmor18269

ATGTCTAGAGGATTTAAAGGGAAATCCCTTCCATACATAGACAGTGATCCCGATTATGAACTACCGAAAGCTAAGAAACAGCGAACCAATAAACCGAATAAACCAGACATGGATTATGCTGTCATAGAAAATCCACAAAATGATGATGAACATTTTGACAATATTTATCGCACAAGTACAATCGTTGGCGGTAAATATCCACATCTTATGAACACCAATAAAGAAATAGCACTAGAAAATACCAAAAACAACGACGCCGGCTTACCAAAATTTTGCATACCTTATGAACAACCCAAATTTAAACTGCCATATAATCCAGTAGTAGCACTGAGAGAAAAATACAAAATAAATCCAAATGTTAAAATCACGCAAACTAAATTTGAAGACCCGAAAAAGTCTAACGAACTGCCTTTAAATTGTACTGTTTTACAAAATTATGCCATTAATTTATCGGCAGGGAAAAGTGCTAATGAAAATTTAAGTGTTAACGCACTTAATTTAACGAAAACCAAAGATGCTGAAACCATCACCGATGACAATATTATTGTAAATGATGAAGAGACAAACTTTATCGCATCTCTAAGGGCCAGCAATATAACGATATCAAGTTTATCTTCGTTATTGGCCAACAAAGATAGTGATGTCAATAATAACTCTAAAATTCAAAGAAACTATGAAGATACTTCAGAAACTATTGATTTACTGGACGATGATGATGACGGCGGTACACCTAATATTGATCCTAATGTATTGAAAATCTTAGAAGATCAGAGCGAAGCAAACAAAAGCTTTATCGGGCTTTTACTGAAAATTTTAACAAAGGGTCACATTATAGTAGCGAACGAAGTAGAGAGTATGCCGACAGAAATCATTCTGGAAAAACTTAATTTAGCAGGAGTCGATGTACTGAAACTTATAGAGCTATTAAAAGTATGCCTCCAGGAGTATTTGGAGTCAAAAAATAAAACCCGTCAGCAGCCGCTTGTTGCAAATAAATCAACAGAGACTGTTCCAAATATTGAAATAAGAACAATTCTAAACCCCCCGACTTTCCAACTTACGACATCAGTAAACGATGGTGGCTATTCATCTTCTGTTAGTACGCAGACAAACGAACCTGCTCTACAAGTTCAGGTCAAGAGCGTCTCCCAGCCGCACTGTCCTTCACAAGACGTGCATTCAATAATTTGTCGACCTAACCAACAATATAATTACTCGCAGCAAACAAATCAAAACACCAATACCGTACAACAACCATCTCAGATCGTTGCACCACGTCAGACTCCTTTCGCAATGCCACATATCCAGGCAGTCATGCCGCAGACAGATTTTGCAAGTGCGATTACTAATTTCCCCGCTATTCCTGCACAAAATATTCAAATTAATTCATCGCAACCACTTCCGCAAAACCAGAACAATTTCCAGCTACCGCAGAATCAAACTGAATCGGTTAATAGGCCATATTACAACACGCAGGGTTCAATGGATTACTCTTACCAGAATTCACAACAGAAACAAGGCTCCGGTAGAAGAAGTTCACAAGAAGTTTATAACTCCAACAATAACAATTCTTCTCTCCGATATGGTGGCCATAGTCGCCCTCGTCAAAGCTTCAATCCGTGCAATAACGAAATGCCTCACATTGGTAATTACTCCGATCGTTATAATCAATACAATAATGTTACGACGGTGACAGAATCATCGTATTCACACATGTTAAGACACCAAAACTCAGTCGATCCGAGGTTTAATTCGTCACAAAATGCAATGGGTTTCTATGATCAGATGGATAATTATGCTTGCAATAATACAAGCAATGTACAATAG

>*Bmor18270*

ATGATTACATTGGATCGTGAATTCTGCAATGTTGTGCATAATCCAGATGATCTAAATAACTTCGTCTATAGCGAACTGCTAACTAATATGAGGAATAGAGACTGGTTATGTGAAAGAGCAATTTTAGCGCCGACGAATGAAATGGTGGGACAGATAAACGAGCAAATTATGTCACGCGTGGAAGGTGATATTGTTGAGTTTCTCTCTGTAGACACTGTAATGGATACTGAACAAGTAACATCATACCCTGTAGAATTTCTTAATTCTTTAGAACTGTCGGGAGTACCTTCACACAAACTGAGGCTGAAAGTAGGCGTTCCTGTCCTTTTAATGCGGAATTTAGATGCACCAAGACTGTGTAATGGCACACGGTTGCAAGTGACACACCTCGGTCGCAATATTGTAAAAGCAATCATTATGACTGGAATGGCAAAAGGAGAGAACGTTTTAATCCCCCGTATACCCATAATTCCGACAGATTTGCCATTCCAGTTTAAGAGATTACAGTTTCCGCTGAAAATCGCATTCGCTATGACGATAAATAAAGCTCAGGGGCAAACATTAAAAGTAGCCGGCATTAATTTAGAAAAGAGTTGTTTTTCTCACGGACAATTATACGTGGCTTGCTCACGTGTTTCAAGTCCACAGAATCTCCATGTGTTGGCCAGAGAGGGAAAAACAAAGAACATAGTTTACAAAAATGTACTACAGTAA

>*Bmor18271*

ATGGGTGTCGTGAGACAAATTCCTGTAGAGTGGTCTCTTGAGGACCTAGTCTCATGGATTGAGTGTCGGTCTGTTTCAACCACCGTCATTAAAGCCAGAAGAATGAATAAGAAAAAGAAAATAGATGGAAAAACAGTATGGGAACCGACGGGTACAGTTGTCCTCTCTTTTCTTGGGCAAGTTCTTCCTAAACATGTATATTGTTGCAGCGTTTCCCTCCCTGTAAACATATATACTCTTCCCACTATTCAGTGTTTAAAGTGTTGTCGTTTTGGGCATATTCGTGATCAATGTCGATCGCAAGCCCGCTGCTCTCGATGCGCAGGCCCCCATGAAGGTAATTCTTGTAGTGTTTCAGAAAATAACATAACCTGCTTGTTCTGCTCTGGTTGTCATCCCGCCTCTGACCCGAAATGCCCCGAACATGCCCGTCAAAGATCAATTAAGATGGTTATGTCTGAAGAAAATATTAGTTATATTGAGGCAGCTCGCAGATTTCCTTCAGTCCGCACCTCCTATGCCGATGTTGCCAATTCTTCTCTTCCCCAAAAACCTATCTTCCTGAGCCGATCAGCTCCATCTTCGAATACCCTCCCGTCTTCCTCCCCCTCATCTTCTCGTCATACTCCGTCAACTTCATACAAAAAAACTGTTTTTATTGAAAAATCGTCTAAACCAATACTTTCCAAGTCTTATGATCGTCATGCTCACAACGATATTATTAGCCCTCCTAACTCTTGTTTACCCAATGGTTGTGCTCTTAGCACTCCCACCCCCTGTGCCCCTGCTACAAATGATAATCTCGCGGAATTAATGTCAATGCTGCTTCTAAATATTTTATCAAAATTCAATGATATACTACCGAACAACGTCTTGATTCAAATTCACGCTTTAATCTCTAGTCTCGTCAACTCTCAACCAAAGCATAATGACGAAGGTCCTACAATGGAACTGTCGTAG

>*Bmor18272*

ATGAAAAAATACTTAAAATACAAGTTCGGGGAGGAGAGCCACTCGCCCCAATATCATGGAGTAGCAGAAATCCCAGAAGCAGAAGCGATGAAAAGAAAGAAACATGAGCAGACAGAGAAGGTTCATCCACCATTACCTAACCGCCCACCGCTGCCGCCGCCGGAACCACTCCTTGCAAATTTCAATAAGGAAATCAACGAAGACGAGGAGACACCTTCAATCACCATAAATATCAAGAAAGATCCAACTGAGAATTATGAAAAGCCTAATGACGTCGAAGAGACCATGTGTCGTAATTTTGTGCGTAACACTTGCAATCGTGGTGCGTCATGTAGATATCTCCACAAAATAATACATTCCCAACTTAAAGGAGTTTACAGATTCTGTATTGATTTTGAAAATAAAAAGTGCACACGTGCAGAATGTTCATATGCTCACGCCACAGTGCACGAGAAAGAACATTTCTTCAGAACGGGCTATTTACCATCACATACATTGAGCCATATTAAGAAAAAAACAGTCCAACCTCCAGCTAAAACCAAAGAAACATCATCCGAGCTGTCGGCGAATTATACCGGCACTACATACCCTCATAGCACCACTTCTGTTCCGATGTACACAAATGCTTCGAATATAAGCATGATGCAAAATCCGTACGAGAAGCAATGGCCGGAAATGGAAGACAAATCGTCGCCCGAGATGGCATATTATAGTGGGCATCCATCTTATCCTCACAGTACGAGCACAGGTTACCCAAACGCTCCGCCCAATACGCCTATAGTGCAAAATCCATACGCCACTGTGATGTCTCCTACGAAGAGGCAGTGGACAGAAATGGAAGAACCCGTGAGTCAACCCACGCACGAGTACGCGGAATACGCTGAAACGGCTGGATCCAAGAAATGCAGAAACTGTGATGTCAACGAGTTCAGATTCCAGCACAACAAAAACAAAATCATGAAAATGATAAAAGACACAGACGACTTGAATTATCGCGTGGGACAAATAACTAAAAAGAACACTAAATTGAATGAAATACTTGTGTTGCTCGTCGAAGTTTTGATAAGACGCACGTATCGCTAG

>*Bmor18273*

ATGACCCACTGCGATTGGTCGGTTGTGTCAATGGGAGCCGGCACCCACCGTGCGTGTATGTCGCCAGCATATGAGTGCGAAGTGTGCGGCAAGCGGTTCGCGGTGGAGCTGCACATGCGGCGGCATCTGTCCACGCACACACTGGAGCGGCCGCGGCGCTGCATGTACTGCGTGTCGGCGCGCGCCTACGTGCGAGGCGAGCAGCTCGTAAAGCACGTGCGCGTGCACCACCCCACCGTGTTCGGCGCGCACCTCAAGCACGTGCGTGCGGTCCTCGGTATCGAAGCGAATACGGTGAGAGTGAAGAAGTCGGAACTAGAGTGTATATTGAACATGCTGGACGCGAAGTCGGATCGGATCATGGACGGCTACGGGGGGACCGGCGTGTTGTACGGCGGCATGCAGGAACAAAAAGATGTCAACGAAAACAGTGACGAAAACATCTCTGTTTTAGAGAAAGTGATCGAGCACTGCGGAGCTAAACCAGCGGATCGCACGATGTGGACGCGCATGCAACGCCTCCGAGAGAACACGAAGCATCTCTTCCTGTACGTGATAGACGACAAGAACATCGCCCGCATGCTGGACACGCACACCATAGACCAGATCGTCAAACATATACTCGCCCAAGTCAGCGACACCGACAAATGA

>*Bmor18274*

ATGTGCGGCTATGCTACACACACGGTAGAAGTAATGCGGCGTCACGTCTTAACTCACGTTACGGATAAACCTTACAAATGCGAGCATTGTAACAGATCGTACATAAAGAAATCACAGCTCCAGCGACATTTAGACAATCATGATATGCAGAAAATGTGCCTTAAATGTGGAGAAACTTTCTCTACAACATGCCAATTATTAGACCTCAAAAACGTACACAAAGGAAATAGCAAATTCGCTTGTCATTACCAAGATTGCCGTTTCTCTGAGAAACCATTTTTAAGTGAGGCTCAATTGCAGAAACACGTAAAAGTTCACCTCGAAGATAGACCATATGAGTGCGAAGTGTGCGGCAAGCGGTTCGCGGTGGAGCTGCACATGCGGCGGCATCTGTCCACGCACACACTGGAGCGGCCGCGGCGCTGCATGTACTGCGTGTCGGCGCGCGCCTACGTGCGAGGCGAGCAGCTCGTAAAGCACGTGCGCGTGCACCACCCCACCGTGTTCGGCGCGCACCTCAAGCACGTGCGTGCGGTCCTCGGTATCGAAGCGAATACGGTGAGAGTGAAGAAGTCGGAACTAGAGTGTATATTGAACATGCTGGACGCGAAGTCGGATCGGATCATGGACGGCTACGGGGGGACCGGCGTGTTGTACGGCGGCATGCAGGAACAAAAAGATGTCAACGAAAACAGTGACGAAAACATCTCTACGCAAAACGATAAAGGACCTTTGATGACAGAAGAGGAGCTTTTGGACAACTTAGGGAAAATGTGTCAAACTGATTGA

>*Bmor18275*

ATGCAAATTTCAGAAACAGGCAATACAAAGTTCCCGAGTATAGGGGATGAAGATTCTGTCAATTTTCGGCCATCGCCGAACTTCAGACTTATGAAAAAATACTTAAAATACAAGTTCGGGGAGGAGAGCCACTCGCCCCAATATCATGGAGTAGCAGAAATCCCAGAAGCAGAAGCGATGAAAAGAAAGAAACATGAGCAGACAGAGAAGGTTCATCCACCATTACCTAACCGCCCACCGCTGCCGCCGCCGGAACCACTCCTTGCAAATTTCAATAAGGAAATCAACGAAGACGAGGAGACACCTTCAATCACCATAAATATCAAGAAAGATCCAACTGAGAATTATGAAAAGCCTAATGACGTCGAAGAGACCATGTGTCGTAATTTTGTGCGTAACACTTGCAATCGTGGTGCGTCATGTAGATATCTCCACAAAATAATACATTCCCAACTTAAAGGAGTTTACAGATTCTGTATTGATTTTGAAAATAAAAAGTGCACACGTGCAGAATGTTCATATGCTCACGCCACAGTGCACGAGAAAGAACATTTCTTCAGAACGGGCTATTTACCATCACATACATTGAGCCATATTAAGAAAAAAACAGTCCAACCTCCAGCTAAAACCAAAGAAACATCATCCGAGCTGTCGGCGAATTATACCGGCACTACATACCCTCATAGCACCACTTCTGTTCCGATGTACACAAATGCTTCGAATATAAGCATGATGCAAAATCCGTACGAGAAGCAATGGCCGGAAATGGAAGACAAATCGTCGCCCGAGATGGCATATTATAGTGGGCATCCATCTTATCCTCACAGTACGAGCACAGGTTACCCAAACGCTCCGCCCAATACGCCTATAGTGCAAAATCCATACGCCACTGTGATGTCTCCTACGAAGAGGCAGTGGACAGAAATGGAAGAACCCGTGAGTCAACCCACGCACGAGTACGCGGAATACGCTGAAACGGCTGGATCCAAGAAATGCAGAAACTGTGATGTCAACGAGTTCAGATTCCAGCACAACAAAAACAAAATCATGAAAATGATAAAAGACACAGACGACTTGAATTATCGCGTGGGACAAATAACTAAAAAGAACACTAAATTGAATGAAATACTTGTGTTGCTCGTCGAAGTTTTGATAAGACGCACGTATCGCTAG

>*Bmor18276*

ATGACGGGTCACTACTTTCGGTATAAAATATTTGCTATATTTAAGGAGCTACAACAAATAGAAAGTACCAACCAAAAACGTCAAAATACTGCAAGGAGACTTTTTGCCAGCTTGGAAGATGTTTCAATCGCTGAAACAAGCCAGAACATCCCACAGCAAGTGCTTCAAGTACCGAACTTGTCACCTCCCCCTCCTACTCTTTCACATACGCCAAGCCCCCTGTCTATGCCCATTGCTTCCCCCTCTCGTCGCGGCACTACCACCACCGCCGGTACCAGCTCTCGCCCCAGCGTAGTAATTCGTCCTCGTCGAAATGATGCACATCCATCAACGGGTCAAGAACGGCCTATTTTGCGGTCATCCTCCCCCATTTCAACGCAAAGAACTCCTTATCGCCGTGGAACTACAGGTAGCTCCAGCCGCAGTAGAGCTCGCATTAGTCGAGAGATTGGATCGTCTTCATCCCGACACAGGCGACATGCTATTTCTCCACCTTCACAACGACGGGCAGAGCTTACTCGTATTACCGAGAGGTTTTTGCGGGTGGAGGAACAGCAGGTGGAGATTCAGCAGGTTTATACGCGTGTTGTTCAAGGCCTTTTGGAACACAGTATCTCTACTCAGTCTCTCATATCTACTGGACATGAGAGACTCGTAGAAATTGTCACGACCATCGGGGAGCGGATAGTCGACCGGCTGTACGAGAGAGAAAGACTATTTGCGGAAGCAGCGTTGTCAATTGGCCAGGGTTTAAAAGCATTGGCAGAAGCTGCTAACGCTTTTAAACCACCACAATAA

>*Bmor18277*

ATGGACTTTAATACATCTAACATTTCCCGGTGTCACAGAATGGGTAAAACATTTACTCCAGAAAGATCTAGGCCCATTCTTTTGAAGCTATGCGACATGAACCTGCGTTCTAAGGTTTGGGCTGCTAAGACTTCGCTTAAGGGCTCAGGAATAACACTCTCCGAGTTCTTGACTAAAAGACGTCATGATATTTTTATGTCTGCACGTCAACGCTTCGGAGTAACCAGGTGCTGGACTCGGGACGGCTGCGTTTACGTTCAGGGATCGGATGGGGTGCGACATTGTGTCGAAGTGATGTCTGAGCTGGATGCTCTTGCCAAGACGACTATGCCAGAAGATGCTGCTCAAAAGAGGGCACAAACCGGCAAGGACACAAAGGAGTCTGCAGCTGCAACCAGACCAAAACGTGTGGCTGCGAACAAGAAGTAG

>*Bmor18278*

ATGTCAAAGTCAAGTACAGGTAGAGGTAAAAATAATAAAACACGTGAACGTTCTCCGAATCCTGGACTGTACCAACAACTTCAAGCGATTGTATCTCGCCTGAATGCGTTGGAAGACAATTCAAGGTCTGGTATAGCGACTGTTGTTGAGAGGCAGCCTGCGGTGGAGTCATCGGGATCGGCGGGCAGTGCTGTGGTGCCGTGCGTGGTTCTGGCTTCTGAGCAAAGCACGACACCGCCATTGGTGCCTACGCCGGTTCCACGAGTTTCCGCTGACGATTGCGTCGCTGCCGAGGTAGCCGGTAAAATTGTAAGCGCAATTAATGAATTGAATCTGGTAAGGCCGAACCATATTTATATATCAAACTTTGATCCCGATTTAAATGATATTAATGTTTGGTGTGATGAAGTTGATCATGCACGTCATTTATAG

>*Bmor18279*

ATGACCAGAGGTACCAAAGCTCGTCAAGTTGATGACCAACTTCAAGTGGCTCTTCAGCAACTAAAAACATCACGGGAACAGTGTCAACAACTACTAAAAGAGAGAGAAGACAACGAAGTCGAAACACTCCAGGTAATAAAGAAAAATACCATGTTGAAAGGACAGTTGTCTCAGCTCTTCATTGAATACAATGAAGTTCTTGAGACTAACAAGAAATTACAAATTGTTGTAGACGGCTTCGATCAGTGCAGCGGTGAGTATGTTGAGTCGCTCCAACTCTCAGCTGAACTGAAAGTCAAACTTTCTGAGGCACATGATTGTATCTCTAATTTACAGTTAGAGATAATAAATCTAAAAGCCCAAAAAACACAGAGCCTGTACTCGGAACTGATCGAGAGTGAACCAGCTTCGGGAACAGTGATCACCACTTCTCAGTGTGACATACCTACAATAGATTTGACTGGTGGTGACTCCGATAGTACACATTCCTCTCGATTAGTTCTTAGTAAGAAAAAGTGTAAGAGATATTCTAAACTAAATAAGTTTATAAATAAAACGGAGAAACTATTAAAACCTAGAAAATCTATTAATTATATAAAAACTATATGCAAAGACCGAAATGCACTGCGCACAAAACTAAATACCTCACTTGAAGAATTACAACTTGTTCATCACAATCATGAGTTAGAAATAGAAAAGTTAACCTCACAAATAAATTCATTGAAAACCGATTTGGACAGTGTCACTAAAAACTATGAATATGCTCAAAAAGACATATGTGAACATATATCGGTGATGGATAACCTGCTTGATTTATGTAATAATAATAAAGAGCGATTTGAATCTTTGACATTACAATATGCTAATTGTAAATGCCACTGCTCTATAGCGGGTTCTCCAGTACCAGACTGTTCCAATAGTCAAAATCAATTTTCTGGTTCTACCTCTAAGCCCGTTGTAAACAACATAATGAAACCTGTGCTTACTAAAAACATTATTAAAATATTCAGTGATGAACTAGGAAAAAATATGGGTGTAGTGCTGAATGATCTGTGTTTGGGACAATCTGTCATAAATTATTGTATGCCTGGGGCTTCATGTACCCACATACTTAATAAAATTTTAAATGTGACTCATTGCCCTAACACAACAATAATTATAATGATTGGGAGAAGGGGAAATGCAGATATAAGAGATATAAAATATCAAATAAATCAATTATTAACACTACAGGTGAAAAAGATCATCATGTTTGCATTTCCATTTATTCAGAGTATGCCAGCAGAAAATAAAATAAGACATAGCATTAATGATTTCATGCAGATAATTTCTAATGTTAATAATTATTATGTCATGACACCATGCAAATCAGAAAATACATTCTATACTATTGACATTAATTGCATGATAAAAAATAAATGTATTATTATGACAAAACCTAACAAATACTATTTAGGTAGCTACAATTTTAGACAAGTAGCAAACTCGCTACATTATTTTTTACATATTTATCTAGCCATGAATTTGGCTAATCAACCTACTGCTTCTTTTGAGCAGGAAAAAAATATGACATTTATTAATTTGGAATCCGTTCCAAGTCATTTAAATTAA

**Dataset 2.** Multi-fasta amino acid sequences of annotated genes on T2T_Dazao W.

>*Bmor18254*

MSEPCDGVGGVSLSDVTFSKNSPEVGAPSSEVGSPPTSARPSGSVNKNMNTSVDGVPADFFTQMLQMMKHVSDRMTSQSDISKVRINDVFLPSYDPDANVGVREWCQHVTIAMETYNLSDYEVRMKAGSLLKGRARLWVDNWLVSTTTWQELRDVLISTFEPENRYSRDVVRFREHTYDNSKDIAQFLSQAWVLWRRITKDKLSNDDAVEAVIGCIGDERLRIELLNARATSVPELISVASSIKSVKRPHPNASIPHQSVAKRPRFDKTPGFCQICKRSGHDTRDCRYRDKPEGPQPRQQDGKTTPPGVGRPTCTFCSRLGHTYQTCYKRERAVVSNVNCVGTSKLNAMPVVVGGFKINAIFDSGAECSVIRESVAAKLPGKRVDRVSYLKALGQFTVVSLSTLTTVCIIDNLRVELEFHVVSDIEVSSDILIGINLIENTNLSVVVNSRGAMLVHRPIIYHMRSQSPKFGKLDCDLTNDNQISELKILLNKFEHLFIHGYPHTRVNTGELEIRLKDESKTVERRPYRLSPVEREKVRDIVNELLEHKIIRESKSPFASPIIIVKKKNGKDRMCVDYRELNRNTVRDHYPLPIISDQIDQLAGGIYFTTIDMAAGFHQIPISEGSIEKTAFVTPDGLYEYLTMPFGLCNAVSVYQRCINRALAHLTSSPDQVCQVYVDDVLSKCKDFDQGLSHIERILIALQEAGFSINVDKTAFFKRSIEYLGNIIADGQVSPSPRKVEALTKAPIPKTVKQVRQFNGLAGYFRRFIPNFSRVMAPLYELTKNDTTWEWNERHDEARDYIVKHLSTAPTLTLFQEDAPIELYTDASSIGYGAVLVQTFAGRQHPVAYMSQRTTDAESRYHSYELETLAVVRAIKHFRHYLYGRKFKVITDCNALKASKNKKDLLPRIHRWWAFLQNYEFEVEYRKGERLQHADFFSRNPCSEMSVNVMTRDMEWLQIEQHRDDVLRPMMESITSGDSIEGYVMEDGILKKMLTDPILGIHKPIVVPKSFQWSLINSFHVTLQHPGWEKTLQKLRETYWFDKMNSAVRRFVDHCVICRTSKGPSGSIQAQLHPIQKPTAAFQVIHMDITGKLGTRNSEGQYEYVIVAIDAFTKYILLSYSNDKSPSSTLAALKRVIHLFGTPVQVVVDGGREFLGEFKVYTDRIGIDVHSTAPGTSRANGQVERVMGTLKNALTMIKNYSTQDWQTALEALQLAFNCTPHRVTGVAPLTLLTRRQHCIPPELLRLVNIENECIDFDLLEQHVRQKMIDAAQYNKQRFDRGKAKLRPFQRGDYVLVKNNTRNQTSLDLKYSEPYEVCRILDNDRYIIKRVTGRGRPRKVAHDQLRRAPQPGEQDTVSTDSVEDPERQPASVPADETETDATSRPQPLPSTSRAAQALDA

>*Bmor18255*

MSFTKKIPRQPLQPIESWKKPDVPFDTVHSDVLGPLPESNGYKFVVLIIDAYSKFCLLNPMRKQDATELKLVFENAVSLFGAPRQIVTDRGRMYENRSFRQWVSDVGSNLFFITPEMHQSNGQAERYCRTVLNMIRVEVNFRNENWSEVLWKLQLTLNVTKQKSTQYSPLYLLIGSDAVTPVIRSVVRDVALEGTSANRETLRELARQRASQLLDKNRKQQDTRVNERRKPPRTFHENDVVFVHKNSQSVGKLDSGMRGPYKIVRALPHGRYELKLLSGSLGKTTEAAAEFISAWHGEWTPDVCTAFFECDENSSEEGIVAGPSHHHDDIVPLDEPINAGEDAPPSGEAVLEDDDDIGGVA

>*Bmor18256*

MNVLQSSLSDLSEHFYKKMADFEAKLHPQSVSSPSTSSPDLAAEFAVFRAFITEAVSGLQKQIEVLSHKLDHMEMHGRRKMLLFHGVVEQKQENTFEIVADILKKHFSLNTVSVHDIKWCNRMRQPSARERARPILVKFHDFSFRNKIWFSKSQLKRTGITVSEFLTKQRHDVFMAARDKFGVTNSWTKEGTVYVLGSDGKRHRVYCRRQLIEIDRHTVETVPVQTAAKPNTASVAPKLKRAAARK

>*Bmor18257*

MKNCNLCKIQFSDGVQCGTCKKHFDFGCANITEAGYRKLGPERRAAWKCSACRNPSQAVASQASPVSPVQGLVTLEMIHAELCGVKTKLNSLSLLHQEVINIREELSLLRESVTQNIEKVSECDARLIEIESKLPVIERTQSSVECLESAMRKMQGRIDALEQRARLNNVEIKGVPVRKNENLFTIVENISQRLGVSFSRADINHVTRVPTFGSADKLIILNFHNRYTKEDFVAAARALKFLNAQDIGFTVSNRIFVNDHLTRDTKQLLTKVKTWAKNKNFKYIWVKFCKIHVRRSDSSPVIIVHTEADLNKLT

>*Bmor18258*

MTEEELLDNLGKMLFKLIDSDTLECFGWPDEPVDVVLEKVIEHCGAKPADRTMWTRMQRLRENTKHLFLYVIDDKNIARMLDTHTIDQIVKHILAQVSDTDK

>*Bmor18259*

MKKYLKFKFGEESHSPQYHGVAEIPEAEAMKRKKHEQTEKVHPPLPNRPPLPPPEPLLANFNKEINEDEETSSITINIKKDPTENYEKPNDVEETMCRNFVRNTCNRGASCRYLHKIIHSQLKEVYRFCIDFENKKCTRAECSYAHATVHEKEHFFRTGYLPPHTLSHIKKKTVQPPAKTKETSSELSANYTGTTYPHSTTSVPMYTNASNISMMQNPYEKQWPEMEDKSSPEMAYYSGHPSYPHSTSTGYPNAPPNTPIVQNPYATVMSPTKRQWTEMEEPVSQPTHEYAEYAETAGSKKCRNCDVNEFRFQHNKNKIMKMIKDTDDLNHRVGQITKKNTKLNEVLVLLVEVVIRRTYR

>*Bmor18260*

MRSQVWFSKTKLKGTGFTVSEFLTKARHGIFMAARSRFGMKKVWTKDGCVYILGEDGIKHRVFTQRDLDRLQKNTSEAIPEVDAAPKRAVTKPAVVKVKRAAARK

>*Bmor18261*

MTGKGRELVDVLKRRRINIACLQETRWKGAKAREIGEGYKLYYSGSDGRRNGVGVVLDKDLKDCVAHVIRTSDRIITVKIVCESVILNVISVYAPQSGCKEDVKEKFWQDFDSVMIRIPESEEICIGGDFNGHLGATNEGYERVHGGWGYGNRNDDGD

>*Bmor18262*

MRKQDATELKLVFENAVSLFGAPRQIVTDRGRMYENRSFRQWVSDVGSNLFFITPEMHQSNGQAERYCRTVLNMIRVEVNFRNENWSEVLWKLQLTLNVTKQKSTQYSPLYLLIGSDAVTPVIRSVVRDVALEGTSANRETLRELARQRASQLLDKNRKQQDTRVNERRKPPRTFHENDVVFVHKNSQSVGKLDSGMRGPYKIVRALPHGRYELKLLSGSLGKTTEAAAEFISAWHGEWTPDVCTAFFECDENSSEEGIVADSSHHHDDIVPLDEPINAGEDAPPSGEAVLEDDDDIGGVA

>*Bmor18263*

MAESKNDDSKNLFYTMDAVPKFTGDDITYSSAKWTQDLDDNADIFGWSQQQKLIIARRCLAGTAELWLKSEKTFRSYEELKVALMKEFPEVINSKQMHETMSRRKKQPNESFYQYMLVMKELGKRAKFPDYVAIQYIIDGISDYESNKLLFYGVTSYSVLKEKLVLYESFKEKSKKSVDTMRRGQRDTAVMPRQVHRCYNCGERGHLSSSCRRGIKCFKCNGFGHKGTECRASQGMEAKKASNNYYGGGDEQDRRSMFVRQQEDDEKNENELYEREQCDLKEQVSDMPNFSFYFLVVNN

>*Bmor18264*

MVNTCIVSGCKRESYSGCGISFHRIPANEERRALWITSMNLDNNVSKFACVCSLHFNQDDYLTEAKKKLKPQAVPKVFPRSKKITVLSIQNMELESSIQPSTSSDITQAGTSCERGESSNKYDVGISGELSYIKVKQEESSTQTPEIKNTPRKAKLRRDVKVLKQKLKKREIKIVNLKSILDLLKKKCENFTELETVLNNSFLNIHKHFVINTKSGKGVRYNEEIKSFALTVCFYSKRAYSYLRNFMCLPHPSTLRKLLSTYNCNVGFMTEVFNFLKSLATKTPSLKNVALIFDSMSIRT

>*Bmor18265*

MSEDYTQGVERLKEVLNVASEYGLEINWKKCELLTKRVDYLGHKIENGTVSPISDKVDAVMNFPVPKTLKQLHSFIGLTSYFRKYIKDYALIATPLTNLLKKNQTFQFTEVERGAFNILKQRLCEKPVLKIYHPDLLTELHTDASKHAYSAILMQRSPDDGELHPVYYSSCKTTESEKKYSSYELEALAVIEGVKKFRKYLLGIPFKIVTDCAAFEMTLRKKDLVTRVARWALLLQEYDYTVEHKSGSRMRHVDALSRNPPYVGTVTRQMHDQIRMAQENDEGLRAIIEVLKSRTYSDYFYENGLLYKTSLMLLVVPKAMQNEVIRKVHGIGHFGKKKMMDLINKDYYIKDLAKCIEDFIVTCIPCILASRKEGKQEGFLHPIEKEDSPLHTIHIDHVGPMTETKKQYNYLLTIVDAFSKFVWIFPTKSTTSKEVLDKLRIHQQHFGNPCRIISDRGTAFTSHEFKQYCEEEGIQHVKITTGIPRGNGQVERIHRIIISVLTKLCIEQPDLWYRHVSRVQQCLNSTFQRSVNCTPFELMVGKRMNIKENVEIHNLLMQEERDLFIEGIEHLRQKAKEQIQLIQEENRRSYNRKRKDSTKYSIGDLVAIRRTQYGTAMKLKPKFLGPYRVTGVKGNDRYEVQKVSANTEGPNNTLSAADYMKQWPVLT

>*Bmor18266*

MTSYNFNDDIQNQLMRFWQLEEVCPQSNHYFPEEKLCEEHFVKNTTRPADGRFCVRIPLKQAPDVLGSSYQRAKHCLLSLERRLKGDDSFSKMYKEFMREYKNLGHMSECKPNIELPHYFIPHHGVLRECSTTTKLRVVFNASSPTSSRVSFNDIQMIGPTVQDDLLSILLSFRQHKYVLVADVEKMYRQIVVHEADRPLQQIIWRENESDSLKSYALNRVSYGTASAPYLASRCSKQIGIECKDPTIADIIIHDFYVDDLLTGSDFISEALRIREQVSAELASACMPLRKWKSNEPQLLSQASHQPSLELKLGDAEPNKTLGLGWDSESDQFHFPVNPVIVKGKTKRDILSVIAQIFDPLGILSPFIISMKLILQRLWLQRLSWDEELPSEIYQAWIETVDSLPHLSSLRIPRRVICDSHSQLELHIFTDASERAYGACLYVRSIDYDDAVSVQLLTAKSRVAPIKPTTIPRLELCGALIGARIYEKCVTALRAKINRVIFWSDSTIVLGWLKMLPSKLQPFVRNRIAEILDKTGSCTWRHVPTDENPADLISRGVSINSLQFHKLWWHGPDYLKRSDSHWPTNPCASDKLPEIRNQVSLLTLSNAPRELIDFSRFSRFSRLLRTVAYVLRFINNLKKGSKRNDKYLSADELQNAKVLIMRHSQMKSFPEMIY

>*Bmor18267*

MDLKLINSLESLEKLFQTRMNDYDLKLQKASTGAVPAHVDISTLSREFNDFKAFVWQTLSQMKTQIELLSLGLDRHETMMRRKVLLLHGIPENKNEKLHKSIVEVFTRNMKLPEICIEHINNCHRLGNSQGKVRPVLVRFCSMEHRNVVWDNKTNLKGSGITVSEFLTKTRHKIFTAARQHFSVGNCWSVEGKIVILTPDKTRHKIECKSELDALTSRYPAAAAIHNSPRTNDDRETAKSQPKVTKSSRRRV

>*Bmor18268*

MSTGIVPWNRCLEHNYTREIVRLMTTLPVPSNRNTLSHSCTERHAQLFLPPAATISVEGGDACAPEYPGTASCKSAGVCSSAGQQMDGVTNRIIGALQVLSKL

>*Bmor18269*

MSRGFKGKSLPYIDSDPDYELPKAKKQRTNKPNKPDMDYAVIENPQNDDEHFDNIYRTSTIVGGKYPHLMNTNKEIALENTKNNDAGLPKFCIPYEQPKFKLPYNPVVALREKYKINPNVKITQTKFEDPKKSNELPLNCTVLQNYAINLSAGKSANENLSVNALNLTKTKDAETITDDNIIVNDEETNFIASLRASNITISSLSSLLANKDSDVNNNSKIQRNYEDTSETIDLLDDDDDGGTPNIDPNVLKILEDQSEANKSFIGLLLKILTKGHIIVANEVESMPTEIILEKLNLAGVDVLKLIELLKVCLQEYLESKNKTRQQPLVANKSTETVPNIEIRTILNPPTFQLTTSVNDGGYSSSVSTQTNEPALQVQVKSVSQPHCPSQDVHSIICRPNQQYNYSQQTNQNTNTVQQPSQIVAPRQTPFAMPHIQAVMPQTDFASAITNFPAIPAQNIQINSSQPLPQNQNNFQLPQNQTESVNRPYYNTQGSMDYSYQNSQQKQGSGRRSSQEVYNSNNNNSSLRYGGHSRPRQSFNPCNNEMPHIGNYSDRYNQYNNVTTVTESSYSHMLRHQNSVDPRFNSSQNAMGFYDQMDNYACNNTSNVQ

>*Bmor18270*

MITLDREFCNVVHNPDDLNNFVYSELLTNMRNRDWLCERAILAPTNEMVGQINEQIMSRVEGDIVEFLSVDTVMDTEQVTSYPVEFLNSLELSGVPSHKLRLKVGVPVLLMRNLDAPRLCNGTRLQVTHLGRNIVKAIIMTGMAKGENVLIPRIPIIPTDLPFQFKRLQFPLKIAFAMTINKAQGQTLKVAGINLEKSCFSHGQLYVACSRVSSPQNLHVLAREGKTKNIVYKNVLQ

>*Bmor18271*

MGVVRQIPVEWSLEDLVSWIECRSVSTTVIKARRMNKKKKIDGKTVWEPTGTVVLSFLGQVLPKHVYCCSVSLPVNIYTLPTIQCLKCCRFGHIRDQCRSQARCSRCAGPHEGNSCSVSENNITCLFCSGCHPASDPKCPEHARQRSIKMVMSEENISYIEAARRFPSVRTSYADVANSSLPQKPIFLSRSAPSSNTLPSSSPSSSRHTPSTSYKKTVFIEKSSKPILSKSYDRHAHNDIISPPNSCLPNGCALSTPTPCAPATNDNLAELMSMLLLNILSKFNDILPNNVLIQIHALISSLVNSQPKHNDEGPTMELS

>*Bmor18272*

MKKYLKYKFGEESHSPQYHGVAEIPEAEAMKRKKHEQTEKVHPPLPNRPPLPPPEPLLANFNKEINEDEETPSITINIKKDPTENYEKPNDVEETMCRNFVRNTCNRGASCRYLHKIIHSQLKGVYRFCIDFENKKCTRAECSYAHATVHEKEHFFRTGYLPSHTLSHIKKKTVQPPAKTKETSSELSANYTGTTYPHSTTSVPMYTNASNISMMQNPYEKQWPEMEDKSSPEMAYYSGHPSYPHSTSTGYPNAPPNTPIVQNPYATVMSPTKRQWTEMEEPVSQPTHEYAEYAETAGSKKCRNCDVNEFRFQHNKNKIMKMIKDTDDLNYRVGQITKKNTKLNEILVLLVEVLIRRTYR

>*Bmor18273*

MTHCDWSVVSMGAGTHRACMSPAYECEVCGKRFAVELHMRRHLSTHTLERPRRCMYCVSARAYVRGEQLVKHVRVHHPTVFGAHLKHVRAVLGIEANTVRVKKSELECILNMLDAKSDRIMDGYGGTGVLYGGMQEQKDVNENSDENISVLEKVIEHCGAKPADRTMWTRMQRLRENTKHLFLYVIDDKNIARMLDTHTIDQIVKHILAQVSDTDK

>*Bmor18274*

MCGYATHTVEVMRRHVLTHVTDKPYKCEHCNRSYIKKSQLQRHLDNHDMQKMCLKCGETFSTTCQLLDLKNVHKGNSKFACHYQDCRFSEKPFLSEAQLQKHVKVHLEDRPYECEVCGKRFAVELHMRRHLSTHTLERPRRCMYCVSARAYVRGEQLVKHVRVHHPTVFGAHLKHVRAVLGIEANTVRVKKSELECILNMLDAKSDRIMDGYGGTGVLYGGMQEQKDVNENSDENISTQNDKGPLMTEEELLDNLGKMCQTD

>*Bmor18275*

MQISETGNTKFPSIGDEDSVNFRPSPNFRLMKKYLKYKFGEESHSPQYHGVAEIPEAEAMKRKKHEQTEKVHPPLPNRPPLPPPEPLLANFNKEINEDEETPSITINIKKDPTENYEKPNDVEETMCRNFVRNTCNRGASCRYLHKIIHSQLKGVYRFCIDFENKKCTRAECSYAHATVHEKEHFFRTGYLPSHTLSHIKKKTVQPPAKTKETSSELSANYTGTTYPHSTTSVPMYTNASNISMMQNPYEKQWPEMEDKSSPEMAYYSGHPSYPHSTSTGYPNAPPNTPIVQNPYATVMSPTKRQWTEMEEPVSQPTHEYAEYAETAGSKKCRNCDVNEFRFQHNKNKIMKMIKDTDDLNYRVGQITKKNTKLNEILVLLVEVLIRRTYR

>*Bmor18276*

MTGHYFRYKIFAIFKELQQIESTNQKRQNTARRLFASLEDVSIAETSQNIPQQVLQVPNLSPPPPTLSHTPSPLSMPIASPSRRGTTTTAGTSSRPSVVIRPRRNDAHPSTGQERPILRSSSPISTQRTPYRRGTTGSSSRSRARISREIGSSSSRHRRHAISPPSQRRAELTRITERFLRVEEQQVEIQQVYTRVVQGLLEHSISTQSLISTGHERLVEIVTTIGERIVDRLYERERLFAEAALSIGQGLKALAEAANAFKPPQ

>*Bmor18277*

MDFNTSNISRCHRMGKTFTPERSRPILLKLCDMNLRSKVWAAKTSLKGSGITLSEFLTKRRHDIFMSARQRFGVTRCWTRDGCVYVQGSDGVRHCVEVMSELDALAKTTMPEDAAQKRAQTGKDTKESAAATRPKRVAANKK

>*Bmor18278*

MSKSSTGRGKNNKTRERSPNPGLYQQLQAIVSRLNALEDNSRSGIATVVERQPAVESSGSAGSAVVPCVVLASEQSTTPPLVPTPVPRVSADDCVAAEVAGKIVSAINELNLVRPNHIYISNFDPDLNDINVWCDEVDHARHL

>*Bmor18279*

MTRGTKARQVDDQLQVALQQLKTSREQCQQLLKEREDNEVETLQVIKKNTMLKGQLSQLFIEYNEVLETNKKLQIVVDGFDQCSGEYVESLQLSAELKVKLSEAHDCISNLQLEIINLKAQKTQSLYSELIESEPASGTVITTSQCDIPTIDLTGGDSDSTHSSRLVLSKKKCKRYSKLNKFINKTEKLLKPRKSINYIKTICKDRNALRTKLNTSLEELQLVHHNHELEIEKLTSQINSLKTDLDSVTKNYEYAQKDICEHISVMDNLLDLCNNNKERFESLTLQYANCKCHCSIAGSPVPDCSNSQNQFSGSTSKPVVNNIMKPVLTKNIIKIFSDELGKNMGVVLNDLCLGQSVINYCMPGASCTHILNKILNVTHCPNTTIIIMIGRRGNADIRDIKYQINQLLTLQVKKIIMFAFPFIQSMPAENKIRHSINDFMQIISNVNNYYVMTPCKSENTFYTIDINCMIKNKCIIMTKPNKYYLGSYNFRQVANSLHYFLHIYLAMNLANQPTASFEQEKNMTFINLESVPSHLN

>*z20-S*

MWTRMQRLRENTKHLFLYVIDDKNIARMLDTHTIDQIVKHILAQVSDTDK
